# Acute Cytokine Responses to High-Intensity Intermittent Exercise in Humans: A Systematic Review

**DOI:** 10.3390/ijms27114950

**Published:** 2026-05-29

**Authors:** Robert Trybulski, Dusko Bjelica, Robert Çitozi, Aleksandra Kisilewicz, Małgorzata Smoter, Joanna Urban

**Affiliations:** 1 Provita Żory Medical Center, 44-240 Żory, Poland; 2 Faculty of Medicine, Katowice Business University, 40-659 Katowice, Poland; 3 Faculty of Sport and Physical Education, University of Montenegro, 81400 Nikšić, Montenegro; dbjelica@ucg.ac.me; 4 Montenegrian Sports Academy, 81400 Nikšić, Montenegro; 5 Department of Physical Activity, Recreation and Tourism, Faculty of Physical Activity and Recreation, Sports University of Tirana, 1001 Tirana, Albania; rcitozi@ust.edu.al; 6 Faculty of Medicine, Wrocław University of Science and Technology, 50-370 Wrocław, Poland; aleksandra.kisielewicz@pwr.edu.pl; 7 Department of Basic Physiotherapy, Gdańsk University of Physical Education and Sport, 80-336 Gdańsk, Poland; malgorzata.smoter@awf.gda.pl; 8 Department of Biological Sciences, Faculty of Science, Thompson Rivers University, Kamloops, BC V2C 0C8, Canada; jurban@tru.ca

**Keywords:** high-intensity intermittent exercise, cytokines, interleukin 6, acute inflammation, time windows

## Abstract

High-intensity intermittent exercise can acutely alter circulating cytokines, but findings are heterogeneous. The aim was to systematically synthesize acute blood cytokine responses after a single high-intensity intermittent exercise session in humans. PubMed, Scopus, and Web of Science Core Collection, plus reference screening. Eligibility criteria included original human studies measuring serum or plasma cytokines pre-exercise and at least one post-exercise time point after high-intensity intermittent exercise. Sampling was mapped to prespecified recovery windows. Risk of bias was assessed using RoB 2 (randomized trials) and the Joanna Briggs Institute quasi-experimental tool. Narrative synthesis was used. From 2077 records, 45 studies were included. Most protocols used cycling or treadmill modalities, and sampling clustered in the immediate and early recovery windows. Interleukin 6 most consistently increased after exercise, whereas tumor necrosis factor alpha, interleukin 10, and other mediators showed mixed or context-dependent changes. Risk of bias was commonly rated as some concerns, with frequent limitations in pre-analytical control and reporting. Across included studies, high-intensity intermittent exercise tended to elicit a short-lived myokine-dominant inflammatory signal, characterized primarily by an increase in circulating interleukin 6, most often detected in the immediate and early recovery windows. Conflicting findings for tumor necrosis factor alpha, interleukin 10, redox-related outcomes, and less frequently measured mediators were best explained by a small set of dominant moderators: post-exercise sampling window, exercise dose/internal load, participant metabolic and training phenotype, and pre-analytical or assay-related heterogeneity. Registration: Open Science Framework (osf.io/wspr6; 17 February 2026).

## 1. Introduction

Regular physical activity and exercise training are associated with lower basal inflammatory tone and improved immunometabolic health, supporting exercise as a non-pharmacological strategy to counter chronic inflammation [1,2]. In contrast to these chronic adaptations, a single bout of exercise acutely perturbs immune homeostasis, inducing rapid leukocyte trafficking and transient changes in circulating inflammatory mediators such as interleukin 6 (IL-6) and C-reactive protein (CRP) [3,4]. These acute perturbations are typically short-lived and are increasingly conceptualized as part of the signaling milieu that initiates tissue remodeling and training adaptation [5,6]. At the cellular level, these transient blood-level mediator changes should be interpreted as downstream outputs of a broader stress-signaling network in which mechanical loading, calcium flux, energetic stress, glycogen availability, lactate accumulation, reactive oxygen/nitrogen species, and neuroendocrine activation converge on AMP-activated protein kinase (AMPK), Calcium/calmodulin-dependent protein kinase II (CaMKII), p38 mitogen-activated protein kinase p38 MAPK/JNK (c-Jun N-terminal kinase), Nuclear factor kappa-light-chain-enhancer of activated B cells (NF-κB), Activator protein 1 (AP-1), and Janus kinase/signal transducer and activator of transcription (JAK/STAT) pathways [7,8]. This framework is particularly relevant for high-intensity intermittent exercise because repeated intense work bouts may generate rapid oscillations in metabolic and redox stress, thereby producing cytokine responses that are nonlinear and potentially hormetic rather than simply proportional to exercise intensity or duration [9,10]. The magnitude and temporal profile of post-exercise cytokine responses depend on exercise dose (e.g., intensity, duration, muscle mass engaged), substrate availability, and host factors such as training status and adiposity [11,12,13,14].

Contracting skeletal muscle functions as a secretory/endocrine organ and releases myokines that coordinate local and systemic crosstalk during and after exercise [15,16]. IL-6 is a prototypical exercise-induced myokine whose production in working muscle can account for the rise in plasma IL-6 during exercise, with intramuscular glycogen availability acting as an important regulatory signal [12,17,18]. In humans, IL-6 elevations at concentrations observed during strenuous exercise can increase anti-inflammatory mediators (e.g., Interleukin-1 receptor antagonist [IL-1] and Interleukin-10 [IL-10]) and attenuate endotoxin-stimulated tumor necrosis factor alpha (TNF-α) production, supporting a basis for an exercise-induced anti-inflammatory cascade [19,20]. Beyond IL-6, acute exercise can modulate chemokines (e.g., Interleukin-8 [IL-8]) and soluble cytokine receptors, while catecholamine signaling contributes to rapid immune-cell mobilization that may shape cytokine kinetics [21,22,23].

High-intensity intermittent exercise (HIIT; including HIIT- and sprint interval-type protocols) is characterized by repeated vigorous or supramaximal work bouts interspersed with recovery and is widely promoted as a time-efficient approach to elicit robust cardiometabolic adaptations [5,24]. However, substantial methodological diversity in interval prescriptions (e.g., work-bout duration, intensity, work-to-rest ratio, and total work) complicates between-study comparisons and likely contributes to heterogeneity in reported inflammatory outcomes [25,26]. Acute HIIT studies commonly report increases in IL-6 with variable accompanying changes in IL-10, IL-8, TNF-α, and monocyte chemoattractant protein-1 (MCP-1), with responses influenced by protocol volume and participant characteristics such as adiposity and fitness [14,22,27]. Comparative studies indicate that intermittent high-intensity exercise can elicit cytokine and acute-phase responses that are sometimes comparable to moderate-intensity continuous exercise, but protocol-specific differences emerge depending on the cytokine measured and the post-exercise sampling window [28]. Across the literature, inconsistent cytokine panels, biological matrices, and sampling schedules constrain inference regarding peak timing and integrated exposure for relevant cytokines after high-intensity intermittent exercise [26,29].

Narrative reviews have consolidated core concepts of exercise-induced cytokine biology and emphasized IL-6 as a central mediator linking acute exercise to anti-inflammatory effects, but they typically integrate diverse exercise modes rather than focusing specifically on high-intensity intermittent protocols [11,18,30]. Systematic reviews and meta-analyses that evaluate HIIT and inflammatory biomarkers have predominantly focused on chronic training effects, often in clinical or at-risk populations, emphasizing resting CRP, IL-6, and TNF-α rather than acute post-session cytokine kinetics [31,32]. A broader systematic review/meta-analysis of interval training and immune outcomes supports that acute interval exercise can alter immune and inflammatory parameters, yet cytokine outcomes are frequently pooled across heterogeneous interval modalities and time points, limiting resolution of protocol- and sampling-dependent determinants [25,33]. Therefore, an up-to-date systematic synthesis focused specifically on acute cytokine responses to high-intensity intermittent exercise, capturing time course features and evaluating moderators such as interval design, training status, sex, adiposity, and assay/sampling methodology, is needed to strengthen interpretation and inform evidence-based exercise prescription [26,27]. Therefore, this systematic review aimed to (i) synthesize acute changes in circulating cytokines and related inflammatory mediators following a single session of high-intensity intermittent exercise in humans and (ii) examine how participant characteristics and exercise protocol variables moderate these responses.

## 2. Materials and Methods

This systematic review was conducted and reported in accordance with the PRISMA 2020 statement and checklist [34]. An a priori review protocol specifying eligibility criteria, outcome definitions, time windows, and planned synthesis approaches was developed and published before study selection began on the Open Science Framework platform (osf.io/wspr6; 17 February 2026).

### 2.1. Eligibility Criteria

Eligibility criteria were defined using a PICOS (Population, Intervention, Comparator, Outcome, Study design) framework. Participants were human adults or children/adolescents of any sex and health status. Interventions were restricted to a single session of high-intensity intermittent exercise, operationalized as repeated high-intensity work bouts interspersed with recovery, including high-intensity interval exercise/training (HIIT/HIIE; typically prescribed relative to physiological or performance indicators such as ≥85–90% HRmax/VO_2_max, vVO_2_max, peak/critical power, or comparable intensity thresholds) and sprint interval-type protocols (SIT; supramaximal or “all-out” efforts with recovery). Comparators were not required. Eligible designs included within-subject pre–post studies, randomized or non-randomized controlled trials, and crossover studies comparing high-intensity intermittent exercise with rest or other exercise prescriptions (e.g., moderate-intensity continuous exercise). Outcomes were circulating cytokines and closely related inflammatory mediators measured in blood-derived matrices (serum or plasma) at baseline (pre-exercise) and at ≥1 post-exercise time point, including (but not limited to) IL-6, IL-10, TNF-α, Interleukin-1 beta (IL-1β), Interleukin-1 receptor antagonist (IL-1ra), Interleukin-8/C-X-C motif chemokine ligand 8 (IL-8/CXCL8), Monocyte chemoattractant protein-1/C-C motif chemokine ligand 2 (MCP-1/CCL2), and interferons. Studies were excluded if they (i) were not original human research (e.g., reviews, editorials), (ii) investigated only chronic training adaptations without acute post-session data, (iii) combined interval exercise with another acute intervention likely to confound cytokine responses (e.g., pharmacological immune challenges) without separable data, or (iv) reported outcomes exclusively in non-blood matrices (e.g., saliva, seminal plasma) unless blood cytokine data were also available.

### 2.2. Information Sources

The following databases were searched from inception to 19 February 2026: PubMed, Scopus, and Web of Science Core Collection. The final included set was supplemented by manual screening of reference lists from included studies and relevant reviews, and by forward citation tracking of key included articles where database tools permitted.

### 2.3. Search Strategy

A general search strategy (adapted to each database’s field tags, controlled vocabulary, and syntax) combined three concept blocks: (1) high-intensity intermittent exercise, (2) cytokines/inflammation, and (3) acute/time course. The example template was as follows:

[Title/abstract] (“high-intensity interval” OR “high intensity interval” OR HIIT OR HIIE OR “aerobic interval training” OR “interval exercise” OR “interval training” OR “high-intensity intermittent” OR “sprint interval” OR SIT OR “repeated sprint*” OR Tabata OR Wingate OR supramaximal OR “all-out”)

AND

[Title/abstract] (cytokine* OR interleukin* OR myokine* OR chemokine* OR “inflammatory marker*” OR inflammation OR “tumor necrosis factor*” OR TNF* OR IL-6 OR IL6 OR IL-10 OR IL10 OR IL-1* OR IL-8 OR CXCL8 OR MCP-1 OR CCL2 OR interferon* OR IFN* OR “C-reactive protein” OR CRP OR hsCRP OR “acute phase”)

AND

[Title/abstract] (acute OR “single bout” OR “one session” OR postexercise OR post-exercise OR recovery OR kinetics OR “time course” OR “time-course”).

No publication-year limits were applied. Language limits were not applied at the search stage. When potentially eligible full texts were not in English, translation was sought where feasible. Database-specific filters were minimized to avoid inadvertent exclusions.

### 2.4. Selection Process

All records were exported to a reference manager (EndNote Online) for de-duplication. Titles and abstracts were screened independently by two authors against the prespecified criteria, followed by independent full-text assessment of all potentially eligible reports. Disagreements at any stage were resolved by consensus discussion, and a third author adjudicated. Reasons for exclusion at the full-text stage were recorded in sufficient detail to populate the PRISMA flow diagram.

### 2.5. Data Collection Process

Data extraction was performed independently by two authors using a pilot-tested extraction form. Extracted entries were cross-checked, and discrepancies were resolved by consensus with re-review by a third author. When outcomes were presented graphically without extractable numerical values, values were digitized using standardized procedures (Webplotdigitalizer), with duplicate digitization to reduce transcription error.

### 2.6. Data Items

For each eligible study, data were sought for all reported circulating cytokines/inflammatory mediators measured pre-exercise and at each reported post-exercise time point that was compatible with the outcome domain. Post-exercise measurements were mapped to prespecified time windows to enable time course syntheses (e.g., immediate ≤30 min; early 31–120 min; intermediate 2–6 h; late 6–24 h; very late >24 h) while retaining exact sampling times for descriptive reporting. Outcomes were circulating cytokines and closely related inflammatory mediators measured in blood-derived matrices (serum or plasma) at baseline (pre-exercise) and at ≥1 post-exercise time point, including (but not limited to) IL-6, IL-10, TNF-α, IL-1β, IL-1ra, IL-8/CXCL8, MCP-1/CCL2, and interferons.

The following variables were extracted: study design (parallel/crossover; controlled/uncontrolled), sample size, participant characteristics (age, sex distribution, health status, adiposity/BMI, training status/fitness when reported, medication status), exercise protocol descriptors (modality, work-bout duration and intensity prescription/verification, recovery duration and type, number of bouts, work-to-rest ratio, total high-intensity work and session duration, warm-up/cool-down), pre-analytical controls (fasting status, time of day, diet and recent exercise standardization, menstrual-cycle considerations when reported), and biospecimen/assay details (serum vs. plasma, collection/processing, storage, assay platform and detection limits).

### 2.7. Study Risk-of-Bias Assessment

Risk of bias was assessed independently by two authors at the full-text stage for all included evidence units. Discrepancies were resolved by discussion and, when needed, adjudication by a third author. Prior to formal appraisal, authors completed a calibration exercise on a purposive sample of studies representing the main design types anticipated in this field (randomized parallel trials, randomized crossover trials, non-randomized controlled studies, and single-group pre–post studies) to harmonize decision rules and interpretation of signaling questions.

Because the included literature comprised heterogeneous study designs, RoB tools were selected a priori based on design. Randomized parallel-group trials were assessed using the Cochrane Risk of Bias tool for randomized trials (RoB 2). Randomized crossover trials were assessed using the RoB 2 crossover variant, with explicit consideration of crossover-specific issues (randomization of condition order, adequacy of washout, and potential period/carryover effects) in addition to standard RoB 2 domains. Non-randomized studies contributing acute-exercise evidence without random allocation (including non-randomized controlled designs and single-group pre–post designs) were assessed using the Joanna Briggs Institute (JBI) critical appraisal checklist for quasi-experimental studies, which applies item-level judgments across domains related to temporal precedence, participant selection/control, comparability/confounding, outcome measurement, retention, and statistical analysis.

For RoB 2 assessments, domain-level judgments were combined using the tool’s algorithm to derive an overall judgment (low risk of bias, some concerns, or high risk of bias). For JBI assessments, item-level judgments were recorded for all checklist items and summarized into a review-defined overall RoB category (low, moderate, or high) using prespecified rules: studies were rated High RoB when one or more major design/analysis limitations were likely to materially distort the acute cytokine response (e.g., outcome-dependent exclusions, substantial unexplained missing outcome data, or non-interpretable exposure/outcome timing); Moderate RoB when there was no single critical flaw but one important limitation or multiple minor limitations were present (e.g., absence of a non-exercise control in a pre–post design, incomplete reporting of pre-analytical controls, or unclear biomarker matrix/assay handling); and Low RoB when limitations were minimal and unlikely to meaningfully influence inference.

Given the physiology of acute cytokine responses, RoB judgments were made with attention to review-relevant sources of bias, including (but not limited to) pre-analytical standardization (time of day, fasting/meal control, recent exercise restriction, illness/medication screening), fidelity of the high-intensity intermittent protocol (achieved intensity, recovery structure, and, when applicable, crossover order and washout), outcome measurement and reporting (biological matrix, assay methods, handling of values below detection, and completeness of reporting across cytokines and post-exercise time points), and missing outcome data (e.g., venipuncture failure, hemolysis, or attrition from the exercise protocol). Where a study included additional acute co-interventions with separable data (e.g., environmental exposure, sleep manipulation, hyperoxia, supplementation), RoB was assessed for the exercise-only evidence unit retained for synthesis; studies without a separable exercise-only condition were excluded at full text.

### 2.8. Synthesis Methods

Studies were considered eligible for a given quantitative synthesis if they reported the same cytokine outcome in a compatible matrix (serum/plasma) at comparable pre- and post-exercise time points that could be assigned to the prespecified time windows, and if interval modality and population grouping were sufficiently homogeneous to support a meaningful pooled estimate. When multiple arms within a study met eligibility (e.g., different interval prescriptions), arms were retained with appropriate handling of shared comparators.

Individual study results were summarized in structured tables and figures. Direction and magnitude of change were described consistently (increase/decrease/no clear change) with corresponding uncertainty.

### 2.9. Certainty of Evidence

The certainty of evidence was assessed using a GRADE-informed approach at the level of each main mediator outcome and recovery window. Outcomes were grouped according to the outcomes, with particular emphasis on interleukin 6, tumor necrosis factor alpha, interleukin 10, and other repeatedly reported inflammatory mediators. Because the review used narrative and evidence-mapping synthesis rather than meta-analysis, certainty judgments were based on the consistency, transparency, and interpretability of the extracted study-level evidence rather than pooled effect estimates.

For each outcome, certainty was classified as high, moderate, low, or very low after considering five domains: risk of bias, inconsistency, indirectness, imprecision, and publication bias. Risk of bias judgments were informed by the Cochrane Risk of Bias 2 tool for randomized trials and the Joanna Briggs Institute checklist for quasi-experimental studies. Inconsistency was judged from the direction, timing, and reproducibility of mediator responses across studies, protocols, populations, and biological matrices. Indirectness considered whether the available evidence matched the review question in terms of high-intensity intermittent exercise exposure, blood-derived cytokine outcomes, and acute post-exercise recovery windows. Imprecision was judged from the number and size of contributing studies, completeness of numerical reporting, reliance on figure-digitized values, and uncertainty around time-point-specific estimates. Publication bias was considered qualitatively, particularly where evidence for a mediator was sparse or concentrated in small single-center studies.

Evidence was rated down when there were important methodological limitations, inconsistent response patterns, sparse data within a recovery window, unclear assay or matrix reporting, incomplete pre-analytical standardization, or dependence on non-tabulated graphical data. Certainty was not upgraded unless there was a clear, reproducible, biologically plausible response pattern across multiple studies and recovery windows. Final certainty ratings were therefore interpreted as confidence in the direction and robustness of the narrative finding, not as certainty around a pooled quantitative effect.

## 3. Results and Discussion

### 3.1. Study Selection

**Figure 1:**
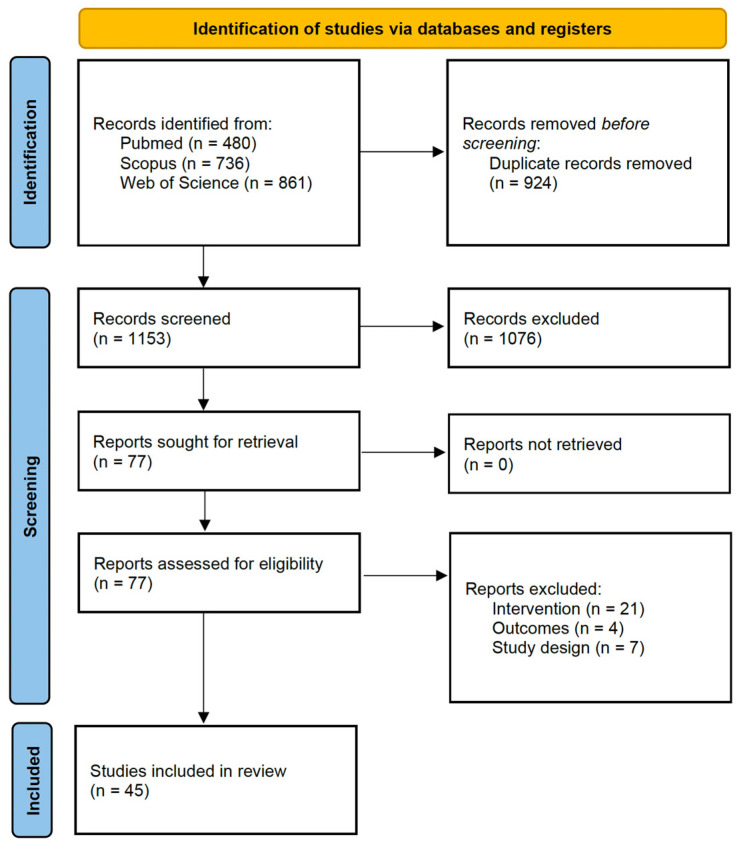
PRISMA flowchart.

A total of 2077 records were identified through database searching, including 480 records from PubMed, 736 from Scopus, and 861 from Web of Science. After removal of 924 duplicates, 1153 unique records remained for screening. Based on title and abstract screening, 1076 records were excluded, and 77 articles were assessed for full-text eligibility. Following full-text review, 32 articles were excluded, resulting in 45 studies being included in the final review. The duplicate rate was 44.5% of all retrieved records, 93.3% of unique records were excluded during title/abstract screening, and 58.4% of full-text articles were ultimately included in the review (Figure 1).

### 3.2. Study Characteristics

Table 1 summarizes the main characteristics of the 45 studies included in this review. Overall, the studies comprised healthy active and sedentary individuals, athletes, adolescents, older adults, and several clinical groups, and most used acute high-intensity interval exercise protocols performed on a cycle ergometer or treadmill, with comparisons against moderate-intensity continuous exercise, alternative interval formats, or control conditions. It also shows substantial heterogeneity in blood collection procedures, although sampling was concentrated predominantly in the immediate post-exercise period and within the early recovery window, with fewer studies extending follow-up to later time points such as 24 or 48 h.

**Figure 2:**
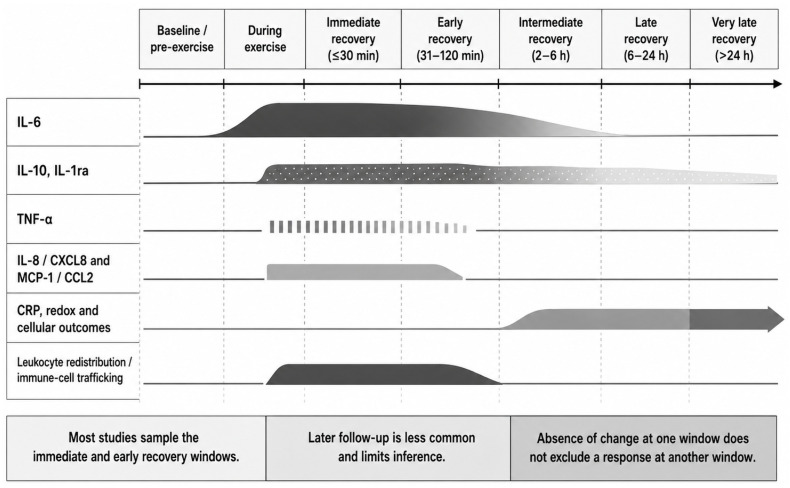
Recovery window timeline for interpreting acute cytokine responses after high-intensity intermittent exercise. The schematic summarizes the prespecified temporal windows used to classify post-exercise blood sampling and illustrates the approximate expected prominence of major cytokine and related outcome groups. Abbreviations: CRP, C-reactive protein; CXCL8, C-X-C motif chemokine ligand 8; IL, interleukin; IL-1ra, interleukin-1 receptor antagonist; MCP-1/CCL2, monocyte chemoattractant protein-1/C-C motif chemokine ligand 2; TNF-α, tumor necrosis factor alpha.

To support interpretation of the acute time course, Figure 2 summarizes the prespecified recovery windows used in this review and maps the approximate temporal prominence of the main cytokine and related outcome groups. This timeline emphasizes that the apparent presence or absence of a cytokine response depends strongly on when blood sampling occurs. Accordingly, null findings at a single post-exercise time point should not be interpreted as evidence that no cytokine response occurred across the full recovery period.

Table 2 summarizes the characteristics of the exercise protocols and comparator conditions across the included studies, detailing the intervention arm, exercise mode, interval structure, recovery periods, and overall dose or training schedule. Overall, most protocols involving HIIT were performed on a cycle ergometer or treadmill, although some studies used running tracks, swimming, resistance-circuit formats, soccer-simulation exercise, elastic-band exercise, or Wingate-based repeated sprint protocols. Considerable heterogeneity was observed in interval prescription, with work bouts ranging from 5 s to 5 min, recovery intervals ranging from passive rest to low-intensity active recovery, and total exposure varying from single acute laboratory sessions to multi-week training programs.

### 3.3. Risk-of-Bias Assessment

Table 3 presents the risk-of-bias assessment for randomized parallel-group trials evaluated with RoB 2. In interpreting these studies, emphasis was placed on the randomization process, deviations from intended interventions, completeness of outcome data, reliability and consistency of cytokine measurement, and selective reporting across biomarkers and post-exercise time points.

Table 4 summarizes the risk-of-bias assessment for randomized crossover trials evaluated with the RoB 2 crossover variant. In addition to the standard RoB 2 domains, this appraisal considered crossover-specific issues such as randomization of condition order, adequacy of washout, and the possibility of period or carryover effects.

Table 5 summarizes the risk-of-bias assessment for evidence units appraised with the JBI quasi-experimental tool. Because many studies in the literature used non-randomized or single-group pre–post designs, particular attention was paid to temporal alignment between the exercise bout and blood sampling, adequacy of participant characterization, control of important pre-analytical and physiological confounders, clarity of outcome measurement procedures, completeness of outcome data, and appropriateness of the statistical analysis.

### 3.4. Results of Individual Studies

Table 6 summarizes the main statistical results and main findings from studies examining acute responses to a single bout of interval exercise (or an acute comparison condition), including crossover comparisons and single-session pre–post designs. Table 7 summarizes the main statistical results and main findings from studies evaluating multi-week training interventions, including parallel-group and single-group designs, and studies that additionally measured acute responses durig the first and/or last training session.

This systematic review synthesized evidence on inflammatory and related systemic responses to interval-based exercise, spanning acute single-session experiments and multi-week interventions. Across acute studies, the most reproducible signal was a transient increase in circulating interleukin 6, typically appearing in the immediate window and often persisting into the early window, with substantial between-study variability in magnitude and in whether responses differed from comparator conditions. The apparent conflicts across studies were not random; they were most plausibly organized by four dominant moderator domains. First, sampling window strongly shaped interpretation, because immediate sampling preferentially captured rapid IL-6 responses, whereas delayed inflammatory, redox, acute-phase, or cellular-functional responses required later sampling. Second, exercise dose and internal load modified the cytokine pattern, with higher interval volume, greater recruited muscle mass, larger metabolic stress, and incomplete recovery tending to produce clearer IL-6 or broader stress-related responses. Third, participant phenotype (especially training status, adiposity, insulin sensitivity, diabetes or inflammatory disease status, sex/hormonal context, and baseline inflammatory burden) limited direct comparability across cohorts. Fourth, pre-analytical and analytical factors, including fasting/fed state, clock time, recent exercise, sleep/circadian context, biological matrix, assay platform, detection limits, and plasma-volume correction, affected whether small and transient cytokine changes could be detected reliably. Responses for tumor necrosis factor alpha, interleukin 10, and other cytokines were therefore less consistent than IL-6, not because they are biologically irrelevant, but because they appear more dependent on timing, dose, phenotype, and measurement context.

### 3.5. Synthesis of Response Patterns and Moderators

Table 8 summarizes the main moderator patterns and the gaps that prevent stronger causal inference. For exercise dose, the evidence should not be interpreted only through a linear more-exercise-produces-more-inflammation model. Instead, the included studies are more consistent with a nonlinear dose–response framework in which the inflammatory profile depends on the interaction between external dose, internal load, participant phenotype, and recovery context. External dose includes interval intensity, number and duration of work bouts, work-to-rest ratio, modality, total high-intensity work, and total session duration, whereas internal load includes metabolic stress, lactate accumulation, glycogen availability, sympathetic activation, redox perturbation, perceived exertion, and fitness-adjusted physiological strain. Several studies suggested that larger interval volume, higher intensity, or greater total work tended to produce clearer IL-6 responses and, in some cases, stronger IL-10-related anti-inflammatory signaling. However, the available studies were too heterogeneous in protocol structure, sampling schedule, assay platform, and reported effect metrics to support formal nonlinear meta-regression or threshold modeling.

Metabolic state should also be interpreted as a biological moderator of acute cytokine responses rather than only as a pre-analytical control variable. Across the included studies, fasting status, meal timing, carbohydrate availability, adiposity, diabetes status, glucose tolerance, insulin, glucose, lactate, non-esterified fatty acids, and related metabolic outcomes were reported inconsistently, and direct measures of muscle glycogen were rarely available. This limitation is important because exercise-induced IL-6 is closely linked to energetic stress in working skeletal muscle, including ATP turnover, AMPK-related signaling, substrate use, and intramuscular glycogen availability.

The evidence base also supports treating circadian, sleep, and time-of-day factors as biological moderators, not only as methodological sources of variability. Some studies controlled time of day, fasting, recent exercise, caffeine, or alcohol exposure, whereas others reported these elements incompletely. In the revised extraction table, testing time was frequently reported but unevenly standardized: many studies used morning or same-time testing, whereas a substantial subset did not clearly report clock time, and only a small number included sleep deprivation, shift-work context, or broader circadian-relevant conditions. This matters biologically because circulating IL-6 shows diurnal variation, cortisol follows a strong sleep–wake/circadian rhythm, and immune-cell trafficking is regulated by circadian, sympathetic, and glucocorticoid signals [79,80]. Therefore, baseline cytokine concentrations, leukocyte availability, cortisol-mediated anti-inflammatory tone, and the magnitude of the post-exercise cytokine response may differ between early-morning, daytime, and evening testing, even when the same interval protocol is used [81,82]. Because most included studies were not designed to compare morning versus afternoon/evening exercise and did not consistently report sleep timing, chronotype, light exposure, prior shift work, or circadian phase markers, formal subgroup analysis by circadian phase was not appropriate. Nevertheless, circadian context should be interpreted as a plausible contributor to between-study heterogeneity, especially for IL-6, cortisol-linked anti-inflammatory signaling, and leukocyte-dependent cytokine outcomes.

Direct measures of intracellular signaling pathways, such as mitogen-activated protein kinase, c-Jun N-terminal kinase, nuclear factor kappa-light-chain-enhancer of activated B cells, activator protein 1, or signal transducer and activator of transcription signaling, were generally absent from the included blood-derived cytokine evidence base.

**Figure 3:**
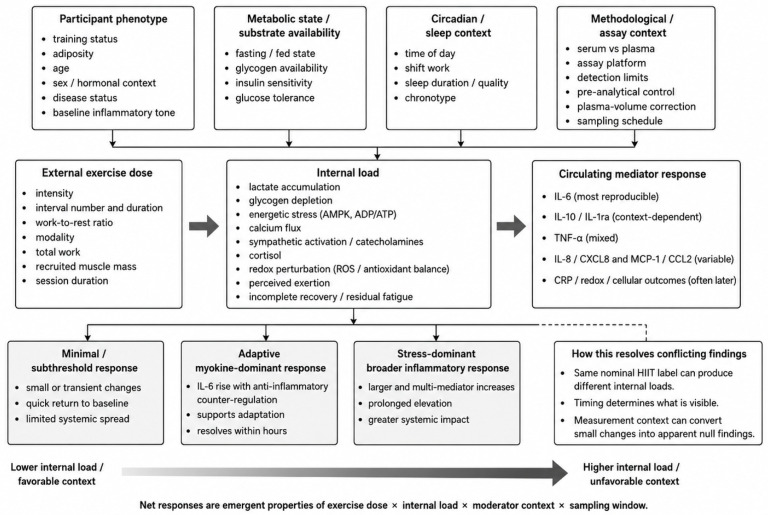
Conceptual model of dominant moderators explaining conflicting cytokine findings. Abbreviations: ADP, adenosine diphosphate; AMPK, AMP-activated protein kinase; ATP, adenosine triphosphate; CRP, C-reactive protein; CXCL8, C-X-C motif chemokine ligand 8; HIIT, high-intensity interval training; IL, interleukin; IL-1ra, interleukin-1 receptor antagonist; MCP-1/CCL2, monocyte chemoattractant protein-1/C-C motif chemokine ligand 2; ROS, reactive oxygen species; TNF-α, tumor necrosis factor alpha.

Because included studies differed substantially in exercise prescription, participant phenotype, sampling schedule, and analytical procedures, conflicting cytokine findings should be interpreted through a moderator-based framework rather than as simple disagreement between studies. Figure 3 summarizes the dominant moderators that can alter the relationship between the externally prescribed exercise dose and the measured circulating cytokine response. This model distinguishes external exercise dose from internal physiological load and highlights why the same nominal high-intensity interval protocol may produce different cytokine patterns across populations and experimental contexts.

### 3.6. Certainty of Evidence

The certainty of evidence was summarized at the outcome level using the Grading of Recommendations Assessment, Development and Evaluation domains (Table 9). Because the review did not include a meta-analysis, certainty ratings apply to the directional narrative conclusion for each outcome rather than to a pooled numerical effect.

### 3.7. Mechanistic Framework: Cellular-Stress Signaling, IL-6, and Hormetic Dose Response

The cytokine patterns summarized in this review can be interpreted within a cellular-stress-response framework in which high-intensity intermittent exercise acts first as a local mechanical, energetic, and redox challenge and only secondarily as a systemic inflammatory stimulus [7,8]. Repeated high-intensity contractions increase ATP turnover, calcium cycling, AMP/ADP accumulation, lactate production, glycogen use, and reactive oxygen/nitrogen species generation, all of which can activate stress-sensitive signaling pathways such as AMPK, CaMKII, p38 MAPK, JNK, NF-κB, and AP-1 [7,8,83].

Human interval exercise studies support the relevance of this framework, since brief intense cycling can activate AMPK and p38 MAPK signaling and increase PGC-1α expression in skeletal muscle, whereas matched high-intensity and continuous running protocols can induce overlapping phosphorylation responses in AMPK, p38, p53, and related transcriptional regulators [8,84].

Within this network, IL-6 should be viewed as a downstream myokine output of contraction-sensitive transcriptional regulation rather than as an isolated circulating inflammatory marker [17,18]. Exercise activates IL-6 gene transcription in working skeletal muscle, and this response is amplified when muscle glycogen availability is low, supporting a mechanistic link between energetic stress and the systemic IL-6 response [85,86]. Experimental evidence also indicates that contraction-induced IL-6 transcription is regulated by JNK/AP-1 signaling, providing a direct bridge between upstream stress-activated kinase pathways and IL-6 expression [87]. Once released, IL-6 may signal through classical and trans-signaling routes and downstream JAK/STAT mechanisms, but circulating IL-6 concentrations alone cannot distinguish which IL-6 signaling mode predominates in a given exercise context. More broadly, circulating cytokine concentrations should be interpreted as extracellular summaries of a distributed cellular network rather than as direct measurements of intracellular signaling. A post-exercise increase in IL-6, IL-8, TNF-α, IL-10, or MCP-1 reflects the net balance between cellular production, release, receptor binding, compartmental redistribution, plasma-volume shifts, and clearance; it does not identify the cellular source, the target-cell population, or the downstream signaling state activated in each tissue. In this sense, cytokine dynamics encode information through amplitude, timing, duration, and combination with other mediators, whereas skeletal muscle cells, leukocyte subsets, endothelial cells, adipose tissue, and hepatic cells decode these extracellular signals according to receptor expression, prior activation state, and intracellular pathway responsiveness. For IL-6 specifically, the same circulating concentration may imply a different biology depending on the relative availability of membrane IL-6 receptor, soluble IL-6 receptor, gp130, and soluble gp130, and on downstream STAT3, MAPK/ERK, PI3K/Akt, and feedback-inhibitory signaling. Therefore, the IL-6 response observed after high-intensity intermittent exercise is best interpreted as a systemic readout of contraction-sensitive and immune–metabolic signaling rather than as direct evidence for a single cellular mechanism. This distinction is important because exercise-induced IL-6 may participate in anti-inflammatory counter-regulation by increasing IL-1 receptor antagonist, IL-10, and cortisol and by suppressing endotoxin-stimulated TNF-α production, while also contributing to substrate mobilization and glucose–lipid metabolism [19].

Moreover, once released, IL-6 can influence substrate metabolism by stimulating lipolysis, increasing fat oxidation, and enhancing insulin-stimulated glucose disposal, thereby linking cytokine signaling to whole-body energy regulation. Consequently, differences in fasting state, recent carbohydrate intake, glycogen availability, insulin sensitivity, adiposity, diabetes status, and glucose tolerance may alter both the magnitude and interpretation of post-exercise IL-6 responses. For this reason, the present synthesis interprets IL-6 as an immunometabolic mediator whose response depends on the interaction between interval dose, internal energetic load, and host metabolic phenotype.

A hormetic interpretation further helps contextualize the heterogeneous responses observed across studies since an insufficient stimulus may fail to activate measurable systemic cytokine release, an appropriate transient stimulus may promote adaptive signaling, and excessive or poorly recovered stress may shift the response toward unresolved inflammation, oxidative damage, or impaired adaptation [9]. A nonlinear dose–response interpretation further helps contextualize the heterogeneous cytokine patterns observed across studies. In this context, exercise dose is not defined only by the external prescription but by the interaction between external workload and internal physiological strain. A short or insufficient stimulus may remain below the threshold needed to produce a measurable systemic cytokine response, especially in trained or highly adapted participants. A moderate-to-high but transient stimulus may produce an adaptive myokine-dominant pattern characterized by short-lived IL-6 release, downstream anti-inflammatory mediators, substrate mobilization, and subsequent resolution. By contrast, an excessive, prolonged, unfamiliar, or poorly recovered stimulus may shift the response toward a stress-dominant regime characterized by larger or more persistent inflammatory, oxidative, neuroendocrine, or muscle-damage signals. Accordingly, the frequent observation of short-lived IL-6 increases after high-intensity intermittent exercise is compatible with an adaptive stress-response model, but the absence of direct measurements of kinase phosphorylation, transcription-factor activation, muscle glycogen, and IL-6 receptor biology in most included studies prevents causal pathway attribution.

**Figure 4:**
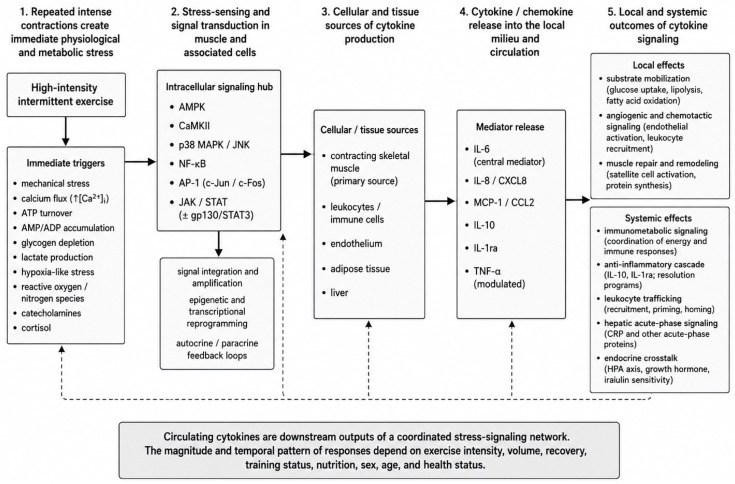
Mechanistic pathway linking high-intensity intermittent exercise to cytokine release. Abbreviations: ADP, adenosine diphosphate; AMPK, AMP-activated protein kinase; AP-1, activator protein-1; ATP, adenosine triphosphate; CaMKII, calcium/calmodulin-dependent protein kinase II; CRP, C-reactive protein; CXCL8, C-X-C motif chemokine ligand 8; HPA, hypothalamic–pituitary–adrenal; IL, interleukin; IL-1ra, interleukin-1 receptor antagonist; JAK, Janus kinase; JNK, c-Jun N-terminal kinase; MCP-1/CCL2, monocyte chemoattractant protein-1/C-C motif chemokine ligand 2; NF-κB, nuclear factor kappa-light-chain-enhancer of activated B cells; STAT, signal transducer and activator of transcription; TNF-α, tumor necrosis factor alpha. Solid line = main direct flow; Intermittent/dashed line = indirect or feedback influence.

To integrate the biological interpretation of the findings, Figure 4 provides a mechanistic pathway linking high-intensity intermittent exercise to cytokine release. The figure should be interpreted as a conceptual synthesis rather than as direct evidence that all pathways were measured in the included studies. It clarifies that circulating cytokine concentrations are downstream outputs of coordinated mechanical, metabolic, redox, neuroendocrine, cellular, and tissue-level processes.

### 3.8. Metabolic State as an Immunometabolic Moderator

Metabolic context may explain part of the between-study heterogeneity in cytokine responses to high-intensity intermittent exercise. Some included studies measured glucose, insulin, lactate, non-esterified fatty acids, appetite-related outcomes, adipokines, or metabolic-disease phenotypes, but these variables were not consistently incorporated into cytokine interpretation across the evidence base. This is important because the same external exercise dose may represent different internal metabolic stress depending on glycogen availability, fasting or fed state, carbohydrate intake, insulin sensitivity, adiposity, disease status, and training adaptation.

The clearest example is IL-6. Exercise-induced IL-6 is produced by contracting skeletal muscle and is amplified under conditions of low glycogen availability, supporting its interpretation as a myokine that integrates inflammatory, energetic, and substrate-regulatory signals [12,17]. IL-6 also has downstream metabolic effects, including stimulation of lipolysis and fat oxidation and enhancement of insulin-stimulated glucose disposal, suggesting that an acute IL-6 increase after interval exercise may reflect adaptive substrate mobilization rather than a purely pro-inflammatory response [88,89]. Accordingly, studies involving overweight or obese participants, diabetes, impaired glucose tolerance, different meal timings, or variable fasting status should not be interpreted as directly interchangeable with studies in lean, insulin-sensitive, fasted, or highly trained cohorts.

### 3.9. Acute Inflammatory Cytokine Responses and the Role of Time Windows

Across heterogeneous interval protocols, interleukin 6 increased acutely in many cohorts, including healthy men and women and several clinical groups. In trained triathletes, interleukin 6 rose immediately after exercise across modalities and intensities, with larger responses after high-intensity interval running compared with low-intensity continuous running [72]. Similar immediate increases were observed after walking protocols irrespective of condition [38], after sprint interval running and cycling in adolescent girls [63], after treadmill interval protocols in adolescents [62] and in phase-comparison sessions in women [61], and after resistance-circuit interval exercise in sedentary young women [48]. In contrast, disease status sometimes appeared to blunt or qualitatively alter the interleukin 6 response: healthy controls increased interleukin 6 one hour after exercise, whereas adults with axial spondyloarthritis did not [37], and adults with type 1 diabetes showed altered patterns across markers in response to interval exercise compared with controls [51].

Mechanistically, the predominance of IL-6 is biologically plausible because contracting skeletal muscle can account for a substantial proportion of the exercise-induced increase in circulating IL-6, supporting its interpretation as a myokine rather than only as a systemic pro-inflammatory cytokine [17,18]. The magnitude of this response is likely shaped by exercise dose, recruited muscle mass, and energetic stress, particularly intramuscular glycogen availability, because low glycogen availability enhances exercise-induced IL-6 transcription and release from working muscle [12,85]. This mechanism may help explain why larger-volume or more metabolically demanding interval protocols tended to produce clearer IL-6 responses, whereas shorter, lower-volume, or highly adapted conditions sometimes produced smaller or less persistent changes [22,27]. Importantly, circulating IL-6 alone cannot determine whether the dominant biological pathway is classical IL-6 signaling or IL-6 trans-signaling, and most included studies did not measure soluble IL-6 receptor, soluble gp130, muscle glycogen, or downstream intracellular signaling; therefore, mechanistic inference should remain plausible but not deterministic. Therefore, the IL-6 response observed in this review should be interpreted as a systemic marker of an upstream cellular-stress-response network, not as direct proof of any single intracellular pathway.

Evidence for acute interleukin 10 responses was mixed and often depended on protocol volume or context. This inconsistency should be interpreted as a timing- and dose-dependent network effect rather than as a simple absence of anti-inflammatory signaling. IL-6 can precede and stimulate anti-inflammatory mediators, including IL-1 receptor antagonist and IL-10, and physiological IL-6 infusion in humans increases IL-1ra, IL-10, and cortisol [19]. In volume-manipulated treadmill interval sessions, the longer session elicited stronger anti-inflammatory signaling, including interleukin 10 changes, than the shorter session [41]. In adolescent girls, interleukin 10 rose in both running and cycling sessions and the tumor necrosis factor alpha to interleukin 10 ratio decreased at 60 min, consistent with a shift toward an anti-inflammatory profile [63]. Conversely, several acute comparisons reported minimal or no change in interleukin 10 [38,75]. These null or inconsistent IL-10 findings do not necessarily contradict an IL-6-linked anti-inflammatory cascade, because IL-10 may require sufficient stimulus volume, may occur after the IL-6 rise, may be compartment-specific, or may be missed when sampling is restricted to the immediate post-exercise period [27]. Tumor necrosis factor alpha responses were likewise inconsistent, rising in some sprint-type protocols or specific subgroups [57,63] but showing no clear effect in others [38,50]. This pattern is biologically plausible because exercise and physiological IL-6 infusion can suppress endotoxin-stimulated TNF-α production in humans; therefore, circulating TNF-α after interval exercise may reflect the balance between inflammatory activation and simultaneous IL-6-linked counter-regulation [20]. Importantly, time window selection likely contributed materially to between-study differences: interleukin 6 and tumor necrosis factor alpha changes were often most evident in the immediate and early windows, whereas downstream markers such as C-reactive protein were more likely to appear in late windows [60].

Several acute studies indicate that context can redirect the inflammatory response. Exposure to traffic-related air pollution shifted the post-exercise pattern away from an interleukin 10-dominant signal observed under filtered air, and attenuated the expected post-exercise reduction in systolic blood pressure [44]. Manipulating oxygen fraction altered oxidative stress responses after all-out interval cycling, with hyperoxic exercise showing smaller increases in oxidative damage markers than normoxic exercise, while cytokine responses were broadly similar across oxygen conditions [58]. These studies underscore that acute inflammatory interpretation should integrate environmental and physiological context, not only the exercise prescription [44,58].

### 3.10. Oxidative Stress and Redox Biology

Redox outcomes were reported using diverse biomarkers, which likely explains why findings were less consistent than for interleukin 6. Some studies reported minimal systemic oxidative stress change [38], whereas others showed distinct exercise-dose effects on redox-related proteins and inflammatory responses. For example, high-intensity interval cycling produced larger interleukin 6 increases and differential regulation of antioxidant-related proteins compared with moderate-intensity continuous cycling [76]. In sedentary women, reactive oxygen species and interleukin 6 increased immediately after resistance-circuit interval exercise following a seated rest period, with partial recovery within 15 min [48]. Hyperoxia reduced the magnitude of oxidative stress marker increases after all-out intervals [58]. At a cellular level, neutrophil redox capacity and antioxidant enzyme activity changed at 24 h after interval exercise in sedentary men, aligning with the possibility that clinically relevant oxidative adaptations may be delayed and compartment-specific rather than captured by immediate systemic markers alone [64].

### 3.11. Cellular and Functional Immune Outcomes

A subset of studies evaluated cellular immune phenotypes and functional readouts. Interval exercise decreased toll-like receptor 2 expression on monocyte subsets and reduced lipopolysaccharide-stimulated tumor necrosis factor alpha release one hour after exercise, with effects observed in adults with type 2 diabetes and healthy controls [46]. In earlier work, adding multiple supramaximal bouts produced larger neutrophil and monocyte responses than a single bout and elicited delayed C-reactive protein elevation at 24 h, again emphasizing that immune perturbations can peak outside the immediate window and may differ markedly by session structure [60]. Together, these findings suggest that relying solely on circulating cytokines may miss meaningful immune modulation detectable in cellular signaling or stimulated functional assays [46,60,64]. These findings suggest that circulating cytokines provide only one layer of the exercise-induced immune response. Cellular assays can reveal whether the same extracellular cytokine pattern is accompanied by altered receptor expression, leukocyte subset redistribution, oxidative burst capacity, phagocytosis, or stimulus-induced cytokine production. This distinction is mechanistically important because a stable circulating TNF-α concentration, for example, may coexist with reduced monocyte Toll-like receptor expression or reduced LPS-stimulated TNF-α release, indicating altered cellular responsiveness despite minimal change in basal plasma cytokine concentration. Similarly, delayed neutrophil phagocytic or redox priming may occur outside the immediate cytokine peak, showing that immune-cell activation state and extracellular cytokine kinetics are temporally related but not interchangeable. Therefore, future interpretations of HIIT-induced immune regulation should distinguish between mediator abundance, receptor-level responsiveness, intracellular pathway activation, and effector-cell function.

### 3.12. Beyond Inflammation: Cardiometabolic, Vascular, Appetite, and Neurobiological Outcomes

Several studies reported outcomes that help contextualize inflammatory findings. Interval exercise influenced appetite and energy intake in overweight men, with timing relative to breakfast modulating relative energy intake and hunger and producing condition-by-time effects in insulin and interleukin 6 [65]. Vascular outcomes showed that the augmentation index improved after continuous moderate exercise but not after high-intensity interval exercise, alongside an interleukin 17 increase only after continuous moderate exercise, indicating that vascular and cytokine responses may not align neatly across protocols [71]. Neurobiological markers were responsive in some contexts: brain-derived neurotrophic factor increased during exercise in older adults with and without chronic obstructive pulmonary disease, with overall responses appearing similar between supramaximal intervals and moderate continuous cycling [55], while distinct protocols elicited different acute profiles for brain-derived neurotrophic factor and catecholamines [75]. Finally, salivary viral deoxyribonucleic acid markers changed more after interval exercise than continuous exercise, paralleling larger interleukin 6 and lactate responses, and were associated with post-exercise force changes [73]. The parallel changes in IL-6, lactate, glucose, non-esterified fatty acids, insulin, appetite-related outcomes, and oxidative stress markers across several studies suggest that IL-6 should be interpreted as part of an integrated immunometabolic response rather than as an isolated inflammatory endpoint [18]. Acute IL-6 can stimulate lipolysis and fat oxidation and can increase insulin-stimulated glucose disposal in humans, providing a plausible biological bridge between the cytokine findings and the metabolic outcomes reported in interval exercise studies [89]. Thus, interval exercise effects are multisystem, and inflammatory markers should be interpreted alongside metabolic, vascular, and neuroendocrine contexts.

### 3.13. Training Interventions

Across multi-week interventions, evidence for improvements in resting inflammation was mixed. In sedentary postmenopausal obese women, four weeks of cycle-based interval training reduced resting interleukin 6 and increased resting interleukin 10 and interleukin 1 receptor antagonist, suggesting a shift toward a less inflammatory baseline state [53]. In overweight women, sixteen weeks of interval training increased resting adiponectin and mitigated declines in bone-related outcomes observed in controls, while acute interleukin 6 rose immediately after exercise on the final training day (Sasimontonkul et al., 2024 [70]). By contrast, six weeks of interval training versus moderate continuous training in obese men produced limited change in fasting inflammatory markers, though acute interleukin 6 and interleukin 10 changed over post-exercise time and interleukin 10 area under the curve differed by training arm (Gerosa-Neto et al., 2020 [49]). In adolescents, both interval and continuous training improved maximum oxygen consumption without clear evidence of a change in the acute interleukin 6 response [54]. These findings collectively suggest that baseline inflammatory adaptation may be more detectable in cohorts with higher baseline inflammatory burden and in studies with sensitive and well-timed measurement schedules, but the direction and magnitude depend on population and intervention structure [49,53,54,70].

An important observation is that training did not always reshape the acute cytokine response. In a short two-week interval training program, the acute inflammatory response profile across sessions remained largely unchanged even while peak power improved [78]. Conversely, some training studies suggested that training status can modify acute recovery kinetics for anti-inflammatory cytokines [59] or attenuate specific inflammatory responses when combined with adjunctive supplementation [36]. Possibly, performance and metabolic adaptations can occur without parallel large shifts in systemic cytokine responses, particularly when training duration is short or baseline inflammation is low [36,59,78].

### 3.14. Circadian and Temporal Biological Regulation of Cytokine Responses

Circadian and sleep–wake biology may contribute meaningfully to heterogeneity in acute cytokine responses to high-intensity intermittent exercise. Circulating IL-6 is not temporally static; human studies indicate diurnal variation in plasma or serum IL-6, with variation across studies in the estimated phase and amplitude of this rhythm [80]. Consequently, the same absolute post-exercise IL-6 concentration or pre-to-post change may have a different interpretation depending on whether the exercise bout occurred in the early morning, daytime, or evening.

Cortisol is also highly relevant because it follows a robust sleep–wake/circadian rhythm and can shape inflammatory tone, leukocyte redistribution, and cytokine production [90]. Human evidence further indicates that cortisol and epinephrine exert opposing regulatory effects on circadian variation in circulating T-cell subsets, supporting a direct link between time of day, neuroendocrine status, immune-cell availability, and cytokine responsiveness [79]. This is important for exercise studies because high-intensity intervals also acutely increase sympathetic activation and stress-hormone signaling, which may interact with pre-existing circadian endocrine state.

Time of day may also modify the inflammatory response to exercise itself. A study [82] directly examining morning-to-evening differences reported that the acute IL-6 response to intense exercise can differ according to the time of day, and another study [81] comparing morning versus evening endurance exercise found time-dependent differences in inflammatory cytokine responses. Although these studies are not all specific to high-intensity intermittent exercise, they support the biological plausibility that clock time and circadian phase could contribute to the inconsistent IL-6, IL-10, and TNF-α patterns observed across the present evidence base.

**Figure 5:**
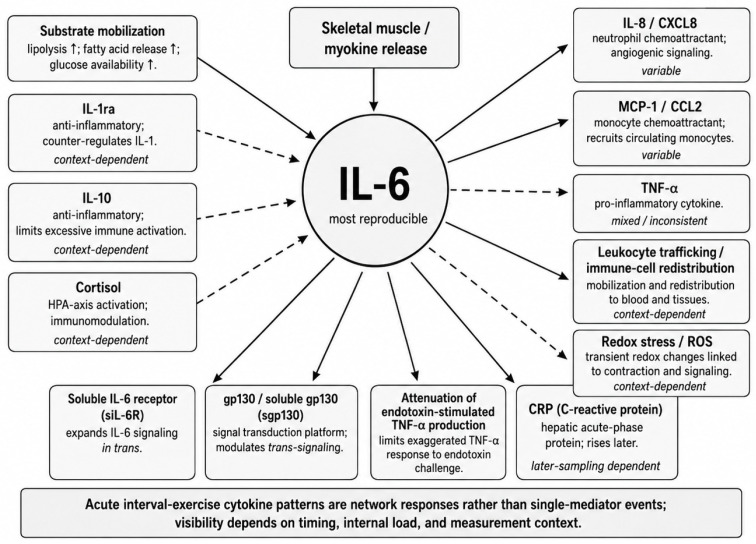
Cytokine-network diagram for acute interval exercise responses. Abbreviations: CRP, C-reactive protein; CXCL8, C-X-C motif chemokine ligand 8; gp130, glycoprotein 130; HPA, hypothalamic–pituitary–adrenal; IL, interleukin; IL-1ra, interleukin-1 receptor antagonist; MCP-1/CCL2, monocyte chemoattractant protein-1/C-C motif chemokine ligand 2; ROS, reactive oxygen species; sgp130, soluble glycoprotein 130; sIL-6R, soluble interleukin-6 receptor; TNF-α, tumor necrosis factor alpha.

Figure 5 extends the mechanistic interpretation by presenting acute exercise-induced cytokine changes as a network response rather than as isolated single-mediator events. This is particularly important for interpreting IL-6, which may function as a myokine, metabolic signal, and upstream component of anti-inflammatory counter-regulation. The network also illustrates why secondary mediators such as IL-10, IL-1ra, TNF-α, chemokines, CRP, redox-related outcomes, and immune-cell trafficking may appear inconsistent when timing, internal load, phenotype, and measurement context differ between studies.

### 3.15. Limitations of the Evidence Base and Implications for Practice and Research

A central practical implication from this body of evidence is that sampling design is not a minor methodological detail since it strongly determines inference. Studies that sampled only immediately after exercise could detect rapid interleukin 6 changes but might miss delayed responses in C-reactive protein, cell function, or oxidative adaptations that appear in late windows [60,64]. Conversely, studies focused on one-hour sampling could miss immediate peaks or biphasic patterns [37,61]. Between-study comparability was further limited by heterogeneity in assay matrices, analytical handling of non-detects, and the frequent inclusion of multiple outcomes without consistent prioritization of primary endpoints [43,52]. For future work, the field would benefit from harmonized minimum sampling schedules aligned to prespecified post-exercise windows, transparent reporting of analyzed samples per biomarker, and clearer distinction between exploratory panels and prespecified primary outcomes.

The evidence base is constrained by small samples in many acute trials, frequent reliance on male-only cohorts, and substantial heterogeneity in participant characteristics and training status, limiting the ability to draw strong population-specific conclusions. Several studies included clinical groups or subgroup analyses, but these were often underpowered for interaction testing [37,46,51]. Protocol heterogeneity also matters since sprint interval cycling, longer interval cycling, interval running, and resistance-circuit interval exercise may not be interchangeable with respect to inflammatory and redox kinetics [41,48,57,60]. Despite these limitations, the collective evidence suggests that interval exercise commonly elicits a short-lived interleukin 6 response, while other cytokines and redox markers show greater context dependence and may require targeted time windows and more specific immune-functional readouts to interpret the findings.

Future studies should pair circulating cytokine panels with mechanistic readouts that can distinguish upstream cellular-stress signals from downstream systemic-mediator responses, including muscle glycogen or carbohydrate availability, lactate and substrate kinetics, catecholamines, cortisol, soluble IL-6 receptor/sGP130, IL-1ra, stimulated TNF-α production, and intracellular signaling markers such as STAT3, AMPK, calcium/calmodulin-dependent protein kinase II (CaMKII), p38 MAPK, c-Jun N-terminal kinase (JNK), NF-κB, and AP-1. Such designs would allow future studies to test whether IL-6 responses reflect energetic stress, glycogen depletion, redox-sensitive signaling, anti-inflammatory counter-regulation, metabolic substrate mobilization, or disease-specific immune dysregulation. They would also permit formal testing of hormetic dose–response models, in which low or insufficient stress produces little systemic signal, moderate transient stress promotes adaptive signaling, and excessive or poorly recovered stress may produce maladaptive inflammatory or oxidative responses. Future work should also distinguish cytokine abundance from cytokine signaling competence. This requires paired assessment of circulating mediators, cytokine receptors and soluble receptor systems, leukocyte subset composition, cell-specific receptor expression, intracellular phosphorylation events, and functional immune responses. Particularly informative approaches would include phospho-flow cytometry for signal transducer and activator of transcription 3 (STAT3), p38 mitogen-activated protein kinase (MAPK), c-Jun N-terminal kinase (JNK), extracellular signal-regulated kinase (ERK), nuclear factor kappa-light-chain-enhancer of activated B cells (NF-κB p65), and protein kinase B (Akt); ex vivo stimulation assays for lipopolysaccharide-induced TNF-α, IL-1β, IL-6, and IL-10 production; monocyte and neutrophil phenotyping for toll-like receptor 2 (TLR2), toll-like receptor 4 (TLR4), cluster of differentiation 11b (CD11b), cluster of differentiation 16 (CD16), human leukocyte antigen-DR isotype (HLA-DR), C-X-C motif chemokine receptor 2 (CXCR2), and C-C motif chemokine receptor 2 (CCR2); and, where feasible, muscle biopsy or single-cell transcriptomic/proteomic profiling to localize cytokine production and downstream pathway activation. Such designs would help determine whether a circulating-IL-6 increase reflects myofiber stress signaling, immune-cell activation, endothelial or adipose contribution, receptor-mediated STAT3 activation in target cells, or a coordinated multi-tissue adaptive response. Future studies should also standardize or explicitly stratify the biological timing of exercise and blood sampling, including clock time, habitual sleep–wake schedule, chronotype, prior shift work, light exposure, meal timing, and sleep duration, because these variables may alter baseline IL-6, cortisol, leukocyte trafficking, and the magnitude of post-exercise cytokine responses.

Future studies should be designed to test nonlinear dose–response relationships directly. This will require within-study comparisons of multiple interval doses using harmonized definitions of external dose and internal load, including relative intensity, total high-intensity work, work-to-rest ratio, session duration, lactate, heart-rate load, perceived exertion, substrate availability, and recovery status. Repeated post-exercise sampling should be sufficient to distinguish transient adaptive responses from persistent or delayed inflammatory responses. Where sample size permits, future analyses should test linear, threshold, U-shaped, and inverted-U models rather than assuming monotonic dose–response relationships. Finally, future studies should explicitly test metabolic state as a moderator of cytokine responses. At minimum, studies should report fasting/fed state, meal timing, carbohydrate intake, glucose, insulin, lactate, non-esterified fatty acids, adiposity, diabetes status, medication use, and indices of insulin sensitivity or glucose tolerance. Where feasible, trials should include direct or indirect measures of muscle glycogen, substrate oxidation, metabolic flexibility, and substrate kinetics, because these measures would help distinguish whether IL-6 responses primarily reflect energetic stress, glycogen depletion, substrate mobilization, immune activation, or disease-specific metabolic inflammation.

## 4. Conclusions

Across acute studies, interval-based exercise most consistently elicited a transient increase in circulating interleukin 6, typically within the immediate and early post-exercise windows. Conflicting findings for tumor necrosis factor alpha, interleukin 10, redox-related markers, and less frequently measured mediators were most plausibly explained by dominant moderators rather than by a single uniform inflammatory effect of high-intensity intermittent exercise. The most important moderators were sampling window, exercise dose and internal load, participant metabolic and training phenotype, circadian/pre-analytical context, and blood matrix or assay methodology. Multi-week interventions showed variable effects on resting inflammation, with some evidence of improved baseline profiles in higher-risk cohorts, but many programs produced limited change in fasting cytokines despite clear fitness or performance gains. Overall, the evidence supports interval exercise as a meaningful acute physiological stimulus, but the inflammatory and related responses are not uniform. The revised synthesis therefore supports a moderator-based interpretation: high-intensity intermittent exercise most often produces a short-lived IL-6-dominant immunometabolic signal, whereas other cytokine and redox responses depend more strongly on timing, dose, phenotype, and measurement context. Future studies should prioritize harmonized sampling schedules aligned to prespecified time windows, transparent reporting of analyzed samples per biomarker, and greater use of cellular or functional immune measures to improve mechanistic interpretability and comparability across trials.

## Figures and Tables

**Table 1:** Methodological characteristics of the included studies.

Study	Participants	Design and Comparison	Cytokine/Mediator Scope and Prioritization	Pre-Analytical Control	Blood Matrix and Assay Reporting	Sampling Schedule and Recovery Window Coverage
Abedelmalek et al., [35]	13 male football players; age 21.1 years (19–24); body mass index 22.6 kg per square meter (18.47–24.46)	Within-participant crossover; baseline sleep vs. partial sleep deprivation; morning session at 08:00	Targeted outcomes: Interleukin 6; tumor necrosis factor alpha	Control level: Stronger standardization Feeding: Before morning testing, only one glass of water was allowed; no food or stimulant intake was allowed after early awakening in the partial sleep deprivation condition Time: Testing performed at 08:00 Recent/stimulants: Participants were nonsmokers and did not consume caffeine or alcoholic beverages; standard eating times were required before the study Sleep/circadian: Participants slept in the laboratory for four consecutive nights before assessment; sleep was monitored by wrist actigraphy	Matrix ambiguity; Detection/CV incomplete Matrix: Matrix reporting is inconsistent: cytokine results are described as plasma concentrations, while the methods describe serum tubes; Assay: Commercial enzyme-linked immunosorbent assay kits for interleukin 6 and tumor necrosis factor alpha Detection/precision: Intra-assay coefficients of variation: interleukin 6, 1.6 to 6.8 percent; tumor necrosis factor alpha, 1.6 to 10 percent. Inter-assay coefficients of variation: interleukin 6, 7. Plasma-volume correction: Corrected using the method of Costill and Fink	Coverage: Immediate/early only Schedule: Before exercise after 15 min of rest; after the first run; after the fourth run; 60 min after exercise Mapped windows: During exercise; immediate recovery; early recovery
Afzalpour et al. [36]	24 sedentary overweight women; age 20–30 years; body mass index greater than 25 kg per square meter; 3 groups (8 per group)	Randomized placebo-controlled parallel groups; acute exercise tested before training and 72 h after last training session	Targeted outcomes: Intercellular adhesion molecule 1; monocyte chemoattractant protein 1; interleukin 10	Control level: Limited/unclear standardization Feeding: Participants arrived after 10 to 12 h of fasting Time: Training sessions were performed at 08:00; acute challenge timing was not explicitly stated Recent/stimulants: Exclusion criteria included smoking, medication use, supplementation with ginger, antioxidant, or multivitamin products; Sex/hormonal: Samples were collected while participants were in the follicular phase of the menstrual cycle	Matrix specified; Detection/CV incomplete Matrix: Serum Assay: Commercial assay kits; the article does not explicitly name the analytical platform Detection/precision: Intra-assay coefficients of variation: intercellular adhesion molecule 1, less than 8 percent; monocyte chemoattractant protein 1, less than 8 percent; interleukin 10, equal to 3. Plasma-volume correction: Corrected using the method of Dill and Costill	Coverage: Immediate only Schedule: Immediately before and immediately after the acute exercise challenge; repeated before the intervention and 72 h after the final training session Mapped windows: Immediate recovery
Andersson et al. [37]	21 adults (10 with axial spondyloarthritis, 11 healthy controls); 10 women; age 18–50 years, mean 40 years; most patients used anti-inflammatory medication	Controlled pre–post pilot study; axial spondyloarthritis versus age- and sex-matched healthy controls; single acute session	Broad/exploratory panel: Interleukin 6; interleukin 17; interleukin 18; tumor necrosis factor alpha; C-X-C motif chemokine ligand 10; vascular endothelial growth factor A; C-reactive protein	Control level: Partial standardization Feeding: Participants were asked to avoid food and drinks for two hours before arrival Time: Time of day was not explicitly reported Recent/stimulants: Participants were asked to avoid tobacco for 30 min before arrival and high-intensity exercise the evening before testing Sex/hormonal: Not reported	Matrix specified; Detection/CV reported Matrix: Serum Assay: Luminex MAGPIX for cytokine, chemokine, myokine, and bone-panel analytes; Detection/precision: Lowest detection limits reported for all analytes: interleukin 6, 0.18 picograms per milliliter; interleukin 17, 0.89 picograms per milliliter; interleukin 18, 0. Plasma-volume correction: Not reported	Coverage: Early only Schedule: Before exercise and one hour after the high-intensity interval bout ended Mapped windows: Early recovery
Brown et al. [38]	17 healthy recreationally active men; age 22.6 ± 4.6 years; maximal oxygen uptake 53.7 ± 7.1 milliliters per kilogram per minute; nonsmokers; no medication	Randomized crossover; high-intensity intermittent walking versus continuous moderate walking; 7-day washout	Targeted outcomes: Interleukin 6; tumor necrosis factor alpha	Control level: Partial standardization Feeding: Participants completed testing after a standard ten-hour overnight fast and replicated dietary intake before the second trial Time: Time of day was not explicitly reported Recent/stimulants: Participants refrained from exercise and alcohol consumption for 24 h before each trial	Matrix specified; Detection/CV incomplete Matrix: Plasma for interleukin 6 and tumor necrosis factor alpha; serum for endothelin 1, lipid hydroperoxides, and hydrogen peroxide; plasma for ascorbyl radical and lipid-soluble antioxidants Assay: Enzyme-linked immunosorbent assay for interleukin 6, tumor necrosis factor alpha, and endothelin 1; ferrous iron xylenol orange assay for lipid hydroperoxides Detection/precision: Assay detection limits and coefficients of variation were not clearly reported for the inflammatory mediators in the extracted text Plasma-volume correction: Not reported	Coverage: Includes very late (>24 h) Schedule: Before exercise; immediately after exercise; 2 h, 4 h, 24 h, and 48 h after exercise Mapped windows: Immediate recovery; early recovery; intermediate recovery; late recovery; very late recovery
Brzezinska et al. [39]	28 healthy untrained young men analyzed; mean age 20.3 years; randomized to ischemic preconditioning (15) or sham (13)	Single-blind randomized controlled parallel groups; ischemic preconditioning versus sham; acute exercise tested before and after 14-day intervention	Broad/exploratory panel: Interleukin 6; interleukin 10; interleukin 15; leukemia inhibitory factor; growth differentiation factor 15; follistatin-like 1	Control level: Partial standardization Feeding: Participants followed a standardized eating pattern before the study; intervention sessions were performed before any meal Time: Testing was described as early morning; cuff intervention sessions were performed between 08:00 and 10:00 Recent/stimulants: Participants abstained for one month from alcohol and substances that could influence performance, including caffeine, guarana, theine, and chocolate	Matrix specified; Detection/CV incomplete. Matrix: Serum Assay: MAGPIX fluorescence-based detection system with Luminex assay kits for inflammatory and neurotrophic markers Detection/precision: Assay detection limits and coefficients of variation were not clearly reported for the inflammatory and neurotrophic markers in the extracted text Plasma-volume correction: Not reported	Coverage: Immediate/early only Schedule: Immediately before, directly after within 5 min, and 2 h after the double Wingate anaerobic test; repeated before and after the fourteen-day intervention Mapped windows: Immediate recovery; early recovery
Cabral-Santos et al. [40]	8 physically active men; age 24.6 years; body mass index 24.3 kg per square meter; peak oxygen uptake 59.9 milliliters per kilogram per minute	Randomized crossover; high-intensity intermittent exercise versus continuous moderate exercise; sessions separated by at least 72 h	Focused multi-mediator set: Interleukin 6; interleukin 10; tumor necrosis factor alpha; interleukin 10 to tumor necrosis factor alpha ratio	Control level: Stronger standardization Feeding: Participants were asked to abstain from eating or drinking for two hours before testing and to maintain their nutritional and hydration routines Time: All tests took place at the same time of day for each participant Recent/stimulants: Participants were instructed to abstain from strenuous exercise for at least 24 h before each testing session	Mixed matrices; Detection/CV incomplete Matrix: Plasma and serum were collected; cytokine matrix was not explicitly specified Assay: Enzyme-linked immunosorbent assay commercial kits for interleukin 6, interleukin 10, and tumor necrosis factor alpha Detection/precision: Assay precision and detection limits were not clearly reported Plasma-volume correction: Not reported	Coverage: Immediate/early only Schedule: At rest; immediately after exercise; 30 min after exercise; 60 min after exercise Mapped windows: Immediate recovery; early recovery
Cabral-Santos et al. [41]	10 physically active men; age 25.22 ± 1.74 years; body mass index 24.85 kg per square meter; peak oxygen uptake 59.94 ± 9.38 milliliters per kilogram per minute	Randomized crossover; two high-intensity intermittent exercise volumes (1.25 km vs. 2.5 km); sessions separated by at least 72 h	Targeted outcomes: Interleukin 6; interleukin 10; monocyte chemoattractant protein 1	Control level: Stronger standardization Feeding: Diet was not standardized, but participants were required to eat three hours before testing Time: Tests took place at the same time of day for each participant, between 10:00 and 12:00 Recent/stimulants: Participants were instructed to abstain from strenuous exercise for at least 24 h and avoid stimulants or alcoholic beverages before testing	Mixed matrices; Detection/CV incomplete Matrix: Serum in abstract; plasma and serum were collected and cytokine-specific matrix was not fully explicit Assay: Commercial enzyme-linked immunosorbent assay kits from R and D Systems for interleukin 6, interleukin 10, brain-derived neurotrophic factor, and monocyte chemo… Detection/precision: Assay precision and detection limits were not clearly reported in the extracted text Plasma-volume correction: Not reported	Coverage: Immediate/early only Schedule: At rest, immediately after exercise, and 60 min after exercise Mapped windows: Immediate recovery; early recovery
Casuso et al. [42]	18 trained adults involved in swimming and running for at least 2 years; age 23 ± 4.2 years; maximal running oxygen uptake 67 ± 8.2 milliliters per kilogram per minute	Randomized crossover; sprint interval swimming versus sprint interval running; sessions separated by 7 to 14 days	Broad/exploratory panel: Interleukin 6; interleukin 10; tumor necrosis factor alpha	Control level: Stronger standardization. Feeding: Participants consumed a standardized breakfast at least one hour before arriving; no food was allowed during the two-hour recovery period Time: All procedures were performed in the morning Recent/stimulants: No strenuous exercise was permitted for 72 h before each trial Sex/hormonal: Not reported	Matrix specified; Detection/CV incomplete Matrix: Serum Assay: Milliplex MAP Human Cytokine/Chemokine Magnetic Bead Panel analyzed on a Luminex 200 system; all samples and standards analyzed in duplicate Detection/precision: Intra-assay coefficients of variation: interleukin 6, 5.5 percent; interleukin 10, 7.2 percent; tumor necrosis factor alpha, 6.1 percent. Plasma-volume correction: Not reported	Coverage: Immediate/early only Schedule: Before exercise after ten minutes of rest; approximately three minutes after exercise; two hours after exercise Mapped windows: Immediate recovery; early recovery
Collins et al. [43]	26 sedentary male rotational shift workers; age 38 ± 8 years; body mass index 32.2 ± 6.0 kg per square meter; cytokine analyses *n* = 15	Randomized parallel groups; high-intensity interval exercise versus continuous moderate exercise; morning trial after day off or day shift	Targeted outcomes: Interleukin 1 receptor antagonist; interleukin 6; tumor necrosis factor alpha	Control level: Stronger standardization Feeding: Fasted venous blood sampling was performed; participants reported for laboratory testing between 06:00 and 09:00 Time: Testing performed between 06:00 and 09:00 after a day off or day shift Recent/stimulants: Participants were sedentary, nonsmokers, and free from known cardiometabolic or sleep disorders; recent-exercise restrictions were not detailed in the extracted text Sleep/circadian: Participants wore actigraphy for approximately seven days before testing	Matrix specified; Detection/CV reported Matrix: Plasma for interleukin 6 and interleukin 1 receptor antagonist; serum for tumor necrosis factor alpha Assay: Commercial enzyme-linked immunosorbent assays Detection/precision: Minimum detectable cytokine levels: interleukin 6 less than 5 picograms per milliliter; tumor necrosis factor alpha 1.7 picograms per milliliter Plasma-volume correction: Not reported	Coverage: Immediate/early only Schedule: Before exercise; immediately after exercise; 30 min after exercise; 60 min after exercise Mapped windows: Immediate recovery; early recovery
Cullen et al. [27]	10 healthy active adults; 5 men and 5 women; age 24 ± 4 years; maximal oxygen uptake 49 ± 5 milliliters per kilogram per minute	Counterbalanced within-participant repeated-measures study; LOW versus MOD versus HIGH; 3 sessions within 2 weeks, minimum 3 days apart	Targeted outcomes: Interleukin 6; interleukin 10; soluble interleukin 6 receptor	Control level: Stronger standardization Feeding: Participants refrained from eating or drinking other than water for two hours before testing Time: Participants arrived at the same time of day before each test Recent/stimulants: Participants maintained similar diet and activity; refrained from alcohol, caffeine, and strenuous exercise during the previous twenty-four hours Sleep/circadian: Not specifically a sleep or circadian intervention Sex/hormonal: Not reported beyond inclusion of both sexes; no menstrual-cycle control reported	Matrix specified; Detection/CV reporte. Matrix: Plasma for interleukin 6, interleukin 10, and soluble interleukin 6 receptor; whole blood for messenger ribonucleic acid expression Assay: High-sensitivity enzyme-linked immunosorbent assays for interleukin 6 and interleukin 10 Detection/precision: Interleukin 6 detection limit 0.039 picograms per milliliter; interleukin 6 intra-assay coefficient of variation 3.8 percent plus or minus 2.9 percent Plasma-volume correction: Not reported	Coverage: Immediate only Schedule: Immediately before and immediately after exercise Mapped windows: Baseline; immediate recovery
Cruz et al. [44]	15 healthy physically active men; age 28.0 ± 6.0 years; body mass index 23.1 ± 1.7 kg per square meter; cytokine analyses *n* = 13	Randomized single-blind repeated measures crossover; filtered air versus traffic-related air pollution; counterbalanced order	Broad/exploratory panel: Interleukin 6; interleukin 10; tumor necrosis factor alpha; vascular endothelial growth factor; interleukin 10 to interleukin 6 ratio; interleukin 10 to tumor necrosis factor alpha ratio	Control level: Stronger standardization Feeding: Participants replicated dietary intake for the 24 h before each trial and fasted for two hours before each session Time: Each participant performed the high-intensity interval exercise at the same time and day of week, separated by one week Recent/stimulants: Participants were asked to refrain from vigorous physical activity, caffeine, and alcohol for 48 h before every visit	Matrix specified; Detection/CV reported Matrix: Serum Assay: Custom 13-cytokine Milliplex MAP Human Cytokine/Chemokine Magnetic Bead Panel analyzed with Magpix xMAP technology Detection/precision: Standard-curve fit used for mean fluorescence intensity versus picograms per milliliter; coefficients of variation and detection limits were not specified in the extracted text Plasma-volume correction: Not reported	Coverage: Immediate/early only Schedule: Baseline; 10 min after exercise; 1 h after exercise Mapped windows: Immediate recovery; early recovery
Dorneles et al. [45]	22 untrained healthy men; 10 lean and 12 overweight–obese; age 20–40 years; nonsmokers; no exercise training in prior 6 months	Randomized crossover; high-intensity interval exercise versus moderate-intensity interval exercise; analyzed by lean and overweight–obese subgroup; sessions at least 1 week apart	Broad/exploratory panel: Interleukin 1 receptor antagonist; interleukin 6; interleukin 8; interleukin 10; interleukin 17A; monocyte chemoattractant protein 1	Control level: Stronger standardization Feeding: Participants arrived after at least one hour of fasting Time: All sessions performed between 09:30 and 10:30 Recent/stimulants: Participants refrained from alcohol, coffee, and vigorous physical activity for twenty-four hours; diet was recorded and repeated across exercise trials Sleep/circadian: Not specifically a sleep or circadian intervention	Matrix specified; Detection/CV incomplete Matrix: Serum for cytokines and muscle damage markers; whole blood for leukocyte counts Assay: Commercial enzyme-linked immunosorbent assays for interleukin 1 receptor antagonist, interleukin 6, interleukin 8, interleukin 10, interleukin 17A, and monocyte Detection/precision: Intra-assay coefficient of variation was less than 7.5 percent for cytokine assays; detection limits were not reported Plasma-volume correction: Adjusted using the Dill and Costill approach from hematocrit and hemoglobin	Coverage: Immediate only Schedule: Before exercise; immediately after exercise; thirty minutes after exercise Mapped windows: Baseline; immediate recovery
Durrer et al. [46]	10 adults with type 2 diabetes (5 men, 5 women) and 9 age-matched healthy controls (4 men, 5 women)	Two-group time-series study; type 2 diabetes versus healthy controls; single acute high-intensity interval exercise session	Broad/exploratory panel: Tumor necrosis factor alpha; lipopolysaccharide-stimulated tumor necrosis factor alpha; toll-like receptor 2; toll-like receptor 4	Control level: Stronger standardization Feeding: Exercise was performed four hours postprandial; water was provided ad libitum Time: Exercise began at either 11:00 or 16:00 Recent/stimulants: Participants refrained from exercise for forty-eight hours before the acute exercise trial; type 2 diabetes participants maintained their usual medication schedule Sleep/circadian: No sleep intervention; time of testing was reported Sex/hormonal: Mixed-sex study; no menstrual-cycle control reported	Matrix specified; Detection/CV incomplete Matrix: Plasma for circulating tumor necrosis factor alpha; diluted whole blood culture for lipopolysaccharide-stimulated tumor necrosis factor alpha; whole blood for flow cytometry Assay: MagPIX assays for plasma tumor necrosis factor alpha and whole-blood culture supernatant tumor necrosis factor alpha Detection/precision: Assay panels were specified; coefficients of variation and detection limits were not reported in the extracted text Plasma-volume correction: Not reported	Coverage: Immediate/early only Schedule: Before exercise; immediately after exercise; one hour after exercise Mapped windows: Baseline; immediate recovery; early recovery
Dzik et al. [47]	26 trained adolescent boys (soccer players); age 13.8 ± 0.7 years; randomized to maximal oxygen uptake test (*n* = 12) or repeated Wingate test (*n* = 14)	Randomized parallel groups; maximal oxygen uptake test until exhaustion versus repeated Wingate anaerobic test	Targeted outcomes: Interleukin 6	Control level: Stronger standardization Feeding: Participants performed testing at least three hours after a light breakfast Time: Tests performed at similar times in the morning Recent/stimulants: Participants withdrew from high-intensity workouts for at least forty-eight hours before testing Sleep/circadian: No sleep intervention; morning testing reduced time-of-day variability	Matrix specified; Detection/CV incomplete Matrix: Plasma for interleukin 6, parathyroid hormone, non-esterified fatty acids, and glycerol; serum for 25-hydroxyvitamin D3 Assay: Commercial enzyme-linked immunosorbent assays for 25-hydroxyvitamin D3, interleukin 6, and parathyroid hormone Detection/precision: Interleukin 6 measured with high-sensitivity R&D Systems kit HS600; values reported as mean plus or minus standard error of the mean in Table 2 Plasma-volume correction: Not reported	Coverage: Immediate/early only Schedule: Before exercise; 15 min after exercise; one hour after exercise; additional lactate samples after the first and second Wingate tests Mapped windows: Baseline; immediate recovery; early recovery
Gassner et al. [48]	30 healthy sedentary young women; age 25.0 ± 4.0 years; body mass index 21.4 ± 1.8 kg per square meter; no regular resistance training in prior 6 months	Descriptive repeated-measures pilot study; uncontrolled single-arm; 2 h seated rest before acute exercise	Targeted outcomes: Reactive oxygen species; interleukin 6	Control level: Stronger standardization Feeding: Participants completed 12 h overnight fasting; consumed 0.5 L tap water one hour before first blood analysis and again immediately after first blood analysis Time: Procedures started at 08:30 Recent/stimulants: Participants abstained from physical activity and alcohol for 48 h before the experimental day Sleep/circadian: No sleep intervention; participants fasted overnight; hormonal contraceptive users were excluded after confounder analysis Sex/hormonal: Female-only sample; hormonal contraceptive users were excluded from final analysis	Matrix specified; Detection/CV reported Matrix: Capillary blood for reactive oxygen species; plasma for interleukin 6 Assay: Electron paramagnetic resonance spectroscopy for reactive oxygen species; high-sensitivity enzyme-linked immunosorbent assay for interleukin 6 Detection/precision: Reactive oxygen species calibration and limit-of-detection procedures reported; interleukin 6 kit detectable mean reported as 6.4 picograms per milliliter Plasma-volume correction: Not reported	Coverage: Immediate only Schedule: Baseline after overnight fasting; after two-hour seated rest immediately before exercise; immediately after exercise; 15 min after exercise Mapped windows: Immediate recovery
Gerosa-Neto et al. [49]	22 inactive obese men; 11 per group; mean age about 29 years; body mass index about 34 kg per square meter	Randomized parallel groups; high-intensity interval training versus moderate-intensity continuous training; acute responses assessed in first and last training sessions across 6 weeks	Broad/exploratory panel: Macrophage inflammatory protein 1 alpha; interleukin 6; interleukin 10; tumor necrosis factor alpha; cytokine ratios; lipopolysaccharide-stimulated interleukin 10 and tumor necrosis factor alpha	Control level: Partial standardization Feeding: Fasting sample after 12 h; pre-exercise sample collected at rest 90 min after standardized breakfast Time: Not clearly specified for acute sessions Recent/stimulants: Habitual dietary habits and daily physical activity were maintained during the training period Sleep/circadian: No sleep or circadian manipulation reported	Matrix specified; Detection/CV not specifie. Matrix: Serum for peripheral inflammatory mediators; plasma from lipopolysaccharide-stimulated whole blood culture for ex vivo cytokine release Assay: Peripheral cytokines measured using Quantikine enzyme-linked immunosorbent assay kits Detection/precision: Peripheral assay sensitivities: tumor necrosis factor alpha, 15.6 to 1000 picograms per milliliter; interleukin 6, 3.13 to 300 picograms per milliliter; interleukin 10, 7 Plasma-volume correction: Not reported	Coverage: Immediate/early only Schedule: Fasting; pre-exercise; immediately post-exercise; 30 min post-exercise; 60 min post-exercise during first and last training sessions Mapped windows: Immediate recovery; early recovery
Gokbel et al. [50]	14 healthy nonsmoking sedentary men; age 19.9 ± 0.9 years	Single-arm repeated-measures time series; repeated supramaximal cycling bouts	Targeted outcomes: Adiponectin; interleukin 6; tumor necrosis factor alpha	Control level: Stronger standardization Feeding: Two hours after carbohydrate-rich light breakfast Time: 09:00 to 10:00 Recent/stimulants: No vitamins, minerals, or medications affecting adiponectin or cytokines for at least three months Sleep/circadian: No sleep control reported	Matrix specified; Detection/CV incomplete Matrix: Plasma for adiponectin, interleukin 6, and tumor necrosis factor alpha; serum for myoglobin Assay: Enzyme-linked immunosorbent assay for adiponectin, interleukin 6, and tumor necrosis factor alpha; chemiluminescence for myoglobin Detection/precision: Detection limits and coefficients of variation not reported in extracted text Plasma-volume correction: Not corrected	Coverage: Immediate/early only Schedule: Preexercise; immediately after within one minute; 15 min; 60 min Mapped windows: Baseline; immediate recovery; early recovery
Hall et al. [51]	9 adults with type 1 diabetes (2 women; duration about 12 years) and 9 healthy controls (3 women)	Exploratory case study with control group; type 1 diabetes versus healthy controls; single acute high-intensity interval exercise; follow-up 24 h	Targeted outcomes: Hypoxia-inducible factor 1 alpha; tumor necrosis factor alpha; vascular endothelial growth factor	Control level: Partial standardization Feeding: At least two hours after breakfast; diet, glycemia, and insulin dosage monitored Time: Exact time of day not reported in extracted article section Recent/stimulants: No exercise, alcohol, or caffeinated drinks for 24 h Sleep/circadian: No sleep control reported Sex/hormonal: Not reported; mixed-sex sample	Matrix specified; Detection/CV reported Matrix: Serum for hypoxia-inducible factor 1 alpha, tumor necrosis factor alpha, and vascular endothelial growth factor; capillary blood for blood glucose Assay: Immunoassay for tumor necrosis factor alpha; enzyme-linked immunosorbent assay for hypoxia-inducible factor 1 alpha and vascular endothelial growth factor Detection/precision: Tumor necrosis factor alpha coefficients of variation 6.3 and 3.3 percent; hypoxia-inducible factor 1 alpha and vascular endothelial growth factor coefficients of variation Plasma-volume correction: Not reported	Coverage: Extends to late (6–24 h); no very late Schedule: Rest; immediately after; 24 h after exercise Mapped windows: Baseline; immediate recovery; late recovery
Herranz-Lopez et al. [52]	26 active men with type 1 diabetes; age 29.3 ± 6.3 years; body mass index 25.1 kg per square meter; moderate habitual activity *n* = 12, intense habitual activity *n* = 14	Randomized within-participant crossover; aerobic exercise versus high-intensity interval exercise; sessions separated by at least 72 h	Broad/exploratory panel: Interleukin 1 beta; interleukin 2; interleukin 4; interleukin 6; interleukin 7; interleukin 8; interleukin 10; interleukin 17A; interleukin 22; tumor necrosis factor alpha; interferon gamma	Control level: Partial standardization Feeding: Fasting status for cytokine sampling was not described for the acute sessions Time: Not clearly reported Recent/stimulants: Single sessions separated by at least seventy-two hours; glucose was monitored during exercise sessions	Matrix specified; Detection/CV incomplete Matrix: Plasma Assay: Procartaplex Mix and Match high-sensitivity multiplex immunoassay measured using the Luminex MAGPIX system Detection/precision: Assay precision and detection limits were not reported in the extracted table section; not-detected results were explicitly reported for some cytokines Plasma-volume correction: Not reported	Coverage: Immediate only Schedule: Twenty minutes before and twenty minutes after each exercise session; optional fasting sample after twelve weeks of training Mapped windows: Baseline; immediate recovery; training endpoint
Henke et al. [53]	10 sedentary postmenopausal obese women; age 58.3 ± 3.1 years; body mass index 31.7 ± 1.3 kg per square meter	Single-group quasi-experimental repeated measures; acute responses compared in first and eighth high-intensity interval sessions across 4 weeks of training	Focused multi-mediator set: Interleukin 1 beta; interleukin 1 receptor antagonist; monocyte chemoattractant protein 1; interleukin 6; interleukin 10	Control level: Limited/unclear standardization Feeding: Fasting status was not clearly reported Time: Not clearly reported Recent/stimulants: Participants were sedentary at baseline; training sessions supervised; no recent stimulant-control details were reported Sleep/circadian: No sleep or circadian manipulation reported Sex/hormonal: Postmenopausal status confirmed by estrone and estradiol concentrations; hormone-replacement therapy excluded	Matrix specified; Detection/CV reported Matrix: Blood-derived matrix not explicitly assigned by outcome; heparinized and non-anticoagulant tubes were collected Assay: Commercial enzyme-linked immunosorbent assay kits for cytokines; spectrofluorometric thiobarbituric acid-reactive substances; Griess-method nitrite Detection/precision: Cytokine intra-assay coefficient of variation was less than 7.5 percent Plasma-volume correction: Not reported	Coverage: Immediate only Schedule: Before and immediately after the first and eighth high-intensity interval exercise sessions Mapped windows: Baseline; immediate recovery
Intan et al. [54]	60 healthy untrained adolescents (30 male, 30 female); age 16.1 ± 0.4 years	Parallel-group pre–post training comparison; high-intensity interval training versus moderate-intensity continuous training; 6 weeks, 3 sessions weekly; acute blood sampling in first and last sessions	Targeted outcomes: Plasma interleukin 6	Control level: Stronger standardization Feeding: Exercises were performed after overnight fasting; no breakfast was consumed Time: Training sessions began at 05:00 Recent/stimulants: Participants were untrained and did not take vitamin supplements or anti-inflammatory drugs; school dormitory context standardized daily activity and diet Sleep/circadian: Dormitory schedule standardized rest and daily activities, but no formal sleep manipulation was reported Sex/hormonal: Not reported	Matrix specified; Detection/CV incomplete. Matrix: Plasma Assay: Commercial enzyme-linked immunosorbent assay kit for human interleukin 6 Detection/precision: Assay precision and detection information not reported Plasma-volume correction: Not reported	Coverage: Immediate only Schedule: Before and immediately after exercise in the first training session and at the end of week six Mapped windows: Immediate recovery
Jakobsson et al. [55]	32 older adults (16 chronic obstructive pulmonary disease, 16 healthy controls); mean age about 74 years; 50% women	Randomized crossover trial; supramaximal high-intensity interval training versus moderate-intensity continuous training; additional higher-intensity interval session; visits separated by at least 48 h and completed within 2 weeks	Broad/exploratory panel: Plasma brain-derived neurotrophic factor; clusterin; hepatocyte growth factor; interleukin 6; lactate	Control level: Stronger standardization Feeding: Participants were instructed to avoid caffeine for six hours before visits; ordinary medication routines were maintained Time: Visits two to four were scheduled at the same time of day, either 09:00 or 13:00 Recent/stimulants: Participants were instructed to refrain from vigorous physical activity for forty-eight hours before each visit and to avoid smoking for eight hours before each visit Sleep/circadian: Time of day was controlled across visits to reduce circadian fluctuation	Matrix specified; Detection/CV not specified Matrix: Plasma Assay: Singleplex or multiplex fluorescent bead-based immunoassays and enzyme-linked immunosorbent assays, depending on marker Plasma-volume correction: Changes in plasma volume were considered in selected analyses	Coverage: Immediate only Schedule: Pre-exercise baseline; iso-time during exercise; immediately post-exercise; 30 min post-exercise Mapped windows: During exercise; immediate recovery
Kaspar et al. [56]	7 healthy untrained adults; age 20.9 ± 0.9 years; 1 male, 6 female	Repeated-measures crossover study; single-bout endurance training versus high-intensity interval training; sessions in random order and at least 7 days apart	Broad/exploratory panel: C-reactive protein; interleukin 1 beta; interleukin 6; interleukin 10; monocyte chemoattractant protein 1; interleukin 6 to interleukin 10 ratio	Control level: Partial standardization Feeding: Fasting state was not clearly reported Time: Exercise sessions performed in the morning between 08:00 and 12:00 Recent/stimulants: Participants abstained from exercise for 48 h before and after the interventions; alcohol and caffeine were prohibited during the study Sex/hormonal: Not reported	Matrix specified; Detection/CV reported Matrix: Plasma for cytokines and insulin-like growth factor 1; serum for C-reactive protein Assay: Commercial sandwich enzyme-linked immunosorbent assay kits for interleukin 1 beta, interleukin 6, interleukin 10, monocyte chemoattractant protein 1, and insulin Detection/precision: Lower limit of quantification: interleukin 1 beta less than 1 picogram per milliliter Plasma-volume correction: Not reported	Coverage: Includes very late (>24 h) Schedule: Before exercise; 30 min after exercise; 2 days after exercise Mapped windows: Baseline; immediate recovery; very late recovery
Kon et al. [57]	8 healthy men; age 23.4 ± 1.1 years; body mass index 22.9 ± 0.5 kg/m^2^	Single-arm pre–post study; single bout of high-intensity interval training	Focused multi-mediator set: C1q/tumor necrosis factor-related protein 1; C1q/tumor necrosis factor-related protein 9; high-molecular-weight adiponectin; tumor necrosis factor alpha	Control level: Partial standardization Feeding: Overnight fasting; participants did not eat until the final blood sample Time: Trials conducted between 07:30 and 11:30 Recent/stimulants: Participants were nonsmokers and were not taking medications; recent exercise control was not clearly reported	Mixed matrices; Detection/CV reported Matrix: Serum and plasma Assay: Enzyme-linked immunosorbent assay kits for C1q/tumor necrosis factor-related protein 1, C1q/tumor necrosis factor-related protein 9, high-molecular-weight adiponectin Detection/precision: Reported coefficients of variability: C1q/tumor necrosis factor-related protein 1, 2.6 percent; C1q/tumor necrosis factor-related protein 9, 2.8 percent Plasma-volume correction: Not reported	Coverage: Immediate/early only Schedule: Before exercise; immediately after exercise; 15 min after exercise; 30 min after exercise; 120 min after exercise Mapped windows: Baseline; immediate recovery; early recovery
Kon et al. [58]	8 healthy men; age 23.6 ± 1.1 years; body mass index 22.9 ± 0.6 kg per square meter	Single-blind crossover; normoxic versus hyperoxic high-intensity interval exercise; trials randomized and at least 1 week apart	Broad/exploratory panel: Derivatives of reactive oxygen metabolites; lipid peroxide; heat shock protein 27; biological antioxidant potential; interleukin 6; tumor necrosis factor alpha	Control level: Stronger standardization Feeding: Overnight fasting; no food until final blood sampling Time: Trials performed between 07:30 and 12:30 Recent/stimulants: Nonsmokers; no medication use	Matrix specified; Detection/CV not specified Matrix: Serum for oxidative stress markers, heat shock protein 27, interleukin 6, and tumor necrosis factor alpha; blood lactate measured separately Assay: FREE Carrio Duo; thiobarbituric acid method; enzyme-linked immunosorbent assay; automatic lactate analyzer Detection/precision: Mean plus or minus standard error; assay precision not fully reported Plasma-volume correction: Not reported	Coverage: Immediate/early only Schedule: Before exercise; immediately after exercise; 60 min; 180 min Mapped windows: Baseline; immediate recovery; early recovery
Lira et al. [59]	30 physically active non-obese men; age 26.4 ± 4.2 years; body mass index 25 kg per square meter or lower; 10 per group	Randomized parallel groups; high-intensity intermittent training versus steady-state training versus non-exercising control; acute exercise assessed in first and last training sessions over 5 weeks	Targeted outcomes: Interleukin 6; interleukin 10; tumor necrosis factor alpha	Control level: Limited/unclear standardization Feeding: Overnight fasting followed by standardized breakfast Time: Fasting blood collection after 8 to 12 h; exact clock time not reported Recent/stimulants: Not clearly reported	Mixed matrices; Detection/CV reported Matrix: Matrix assignment not fully explicit; plasma and serum were prepared Assay: Commercial enzyme-linked immunosorbent assay kits for cytokines; commercial glucose kit; colorimetric kit for non-esterified fatty acids Detection/precision: Interleukin 6 sensitivity 0.7 picograms per milliliter; interleukin 10 sensitivity 3.9; tumor necrosis factor alpha sensitivity 5.5 Plasma-volume correction: Not reported	Coverage: Immediate/early only Schedule: Fasting; pre-exercise; immediately after; 30 min for interleukin 10; 60 min Mapped windows: Baseline; immediate recovery; early recovery
Meyer et al. [60]	12 male physical education students; age 26.9 ± 1.5 years; 6–8 h per week recreational sports; not specifically endurance trained	Randomized crossover; control day, single maximal test, and anaerobic training session performed in random order within 5–8 days	Broad/exploratory panel: Interleukin 6; interleukin 8; C-reactive protein; cortisol; neutrophils; CD16-positive monocytes	Control level: Stronger standardization Feeding: Participants arrived at 08:00 after an overnight fast Time: All study visits were morning appointments; first venous sample at 08:30 and exercise timing standardized Recent/stimulants: Participants were instructed to avoid strenuous exercise during the preceding two days Sleep/circadian: No explicit sleep manipulation; morning timing was standardized	Matrix specified; Detection/CV reported Matrix: Plasma for interleukin 6 and interleukin 8; serum for cortisol and C-reactive protein; whole blood for flow-cytometric cell counts Assay: Enzyme immunoassay for interleukin 6 and interleukin 8; enzyme immunoassay for cortisol; turbidimetric C-reactive protein assay Detection/precision: Interleukin 6 assay sensitivity 3 picograms per milliliter; interleukin 8 assay sensitivity 10 picograms per milliliter; cytokine intra-assay coefficients of variation 1.3 to 6 Plasma-volume correction: Corrected for plasma-volume changes	Coverage: Extends to late (6–24 h); no very late Schedule: Rest; fifteen minutes after exercise; two hours after exercise; twenty-four hours after exercise Mapped windows: Baseline; immediate recovery; intermediate recovery; late recovery
Minuzzi et al. [61]	14 physically active women; age 24 ± 2 years; body mass index 22.8 ± 1.9 kg per square meter; regular menstrual cycles; no hormonal contraceptive use in prior 3 months	Within-participant repeated-measures study; same session tested in follicular and luteal menstrual phases identified by self-report cycle tracking	Focused multi-mediator set: Tumor necrosis factor alpha; interleukin 6; interleukin 10; interleukin 17A; cytokine ratios	Control level: Stronger standardization Feeding: Standardized breakfast provided on the day of high-intensity intermittent exercise Time: Not fully specified in the article; testing sessions were scheduled according to menstrual-cycle phase Recent/stimulants: Participants refrained from strenuous effort and ergogenic or alcoholic drinks for at least seventy-two hours before experimental sessions Sleep/circadian: Menstrual-cycle phase was tracked for three months before the study and during the evaluation month Sex/hormonal: Female-only study; follicular and luteal phases evaluated; no hormonal contraceptive use in the prior three months	Matrix specified; Detection/CV reported Matrix: Serum Assay: Enzyme-linked immunosorbent assay for tumor necrosis factor alpha, interleukin 6, interleukin 10, and interleukin 17A Detection/precision: Tumor necrosis factor alpha assay range 15.6 to 1000 picograms per milliliter; interleukin 6 range 3.13 to 100 picograms per milliliter; interleukin 10 range 7 Plasma-volume correction: Not reported	Coverage: Immediate/early only Schedule: Before exercise; immediately after exercise; one hour after exercise Mapped windows: Baseline; immediate recovery; early recovery
Monteiro et al. [62]	19 obese and overweight adolescents (12 boys, 7 girls); age 11–17 years; sedentary	Single-group acute pilot study with sex subgroup comparison; treadmill maximal test, then high-intensity interval exercise 72 h later	Targeted outcomes: Interleukin 6; interleukin 10; tumor necrosis factor alpha	Control level: Stronger standardization Feeding: Participants were instructed to fast for three hours before exercise Time: Afternoon testing under controlled temperature Recent/stimulants: Participants were instructed not to perform physical exercise for 48 h before testing and not to undertake any diet Sex/hormonal: Not reported; mixed-sex adolescent sample	Mixed matrices; Detection/CV incomplete Matrix: Serum reported for inflammatory markers; plasma and serum were both collected Assay: Commercial kits for total cholesterol, triacylglycerol, glucose, non-esterified fatty acids, cytokines, plasminogen activator inhibitor 1, and cortisol Detection/precision: Detection limits and coefficients of variation were not reported in the article Plasma-volume correction: Not reported	Coverage: Immediate only Schedule: At rest before exercise and immediately after the exercise protocol Mapped windows: Baseline; immediate recovery
Narciso et al. [63]	11 untrained adolescent females; age 17.4 ± 0.9 years; post-peak height velocity; body mass index 24 ± 5.1	Randomized crossover study; high-intensity interval running versus high-intensity interval cycling; four laboratory visits over 2 weeks	Focused multi-mediator set: Interleukin 6; interleukin 10; tumor necrosis factor alpha; interleukin 6 to interleukin 10 ratio; tumor necrosis factor alpha to interleukin 10 ratio	Control level: Partial standardization Feeding: Standardized breakfast provided on the morning of each trial; pre-exercise sample collected 1.5 h post-prandial Time: Morning testing between 07:30 and 10:30 Recent/stimulants: Not explicitly stated in the extracted main text beyond exercise-testing scheduling Sex/hormonal: Menstrual-cycle phase was not controlled; authors report this as a limitation	Matrix specified; Detection/CV reported Matrix: Serum Assay: Automated enzyme-linked immunosorbent assay platform for interleukin 6, interleukin 10, and tumor necrosis factor alpha Detection/precision: Inter-assay coefficients of variation: interleukin 6, 9.9 percent; interleukin 10, 6.6 percent; tumor necrosis factor alpha, 8.7 percent Plasma-volume correction: Not reported	Coverage: Immediate/early only Schedule: Pre-exercise, 5 min after exercise, and 60 min after exercise Mapped windows: Baseline; immediate recovery; early recovery
Ottone et al. [64]	12 sedentary men; young adults; nonsmokers; no anti-inflammatory medications or antioxidant supplements	Single-group acute study; high-intensity interval exercise performed 48 h after a maximal cycle test	Focused multi-mediator set: Neutrophil phagocytic capacity; neutrophil reactive oxygen species generation; interleukin 8; neutrophil redox markers	Control level: Stronger standardization Feeding: Standardized breakfast containing 170 g of carbohydrates; 500 milliliters water recommended 2 h before testing Time: Exercise session performed between 07:30 and 08:30 Recent/stimulants: No strenuous physical activity or alcohol for 24 h; no caffeinated beverages, aspirin, or topical corticosteroid for 48 h Sleep/circadian: Participants were advised to sleep 8 h the night before testing	Matrix specified; Detection/CV reported Matrix: Heparinized whole blood for neutrophil isolation; plasma for cytokines and muscle-damage markers Assay: Flow cytometry for neutrophil functional assays; BD Cytometric Bead Array for plasma cytokines Detection/precision: Mean coefficient of variation for each cytokine analyte was less than 10 percent; precision tested at 80, 625, and 2500 picograms per milliliter Plasma-volume correction: Not reported	Coverage: Extends to late (6–24 h); no very late Schedule: Before exercise; 30 min after exercise; 24 h after exercise Mapped windows: Baseline; immediate recovery; late recovery
Panissa et al. [65]	14 inactive overweight men; age 30.2 ± 4.1 years; peak oxygen consumption 33.8 ± 6.6 milliliters per kilogram per minute	Randomized crossover (Williams square); 5 sessions: high-intensity intermittent exercise and steady-state exercise performed 1 h or 2.5 h after standardized breakfast, plus no-exercise control; 72 h to 1 week between sessions	Targeted outcomes: Interleukin 6; insulin; blood lactate	Control level: Stronger standardization Feeding: Participants arrived after at least 10 h of fasting and consumed a standardized breakfast; breakfast provided 20 percent of estimated daily energy needs Time: Experimental protocols began around 08:00; exercise timing was experimentally varied at 1 h or 2.5 h after breakfast Recent/stimulants: Participants maintained usual hydration and diet; same preceding food intake replicated across sessions as far as possible	Mixed matrices; Detection/CV not specified Matrix: Plasma and serum for interleukin 6 and insulin; capillary blood for lactate Assay: Commercial enzyme-linked immunosorbent assay kits for interleukin 6 and insulin; lactate analyzer for capillary blood lactate Detection/precision: All samples were analyzed in identical runs; intra-assay variance was less than 7 percent Plasma-volume correction: Not reported	Coverage: Immediate/early only Schedule: Interleukin 6 and insulin at 1.0, 1.75, 2.5, and 3.25 h after breakfast; lactate before and after exercise; ad libitum meal at 3.5 h after breakfast Mapped windows: Baseline relative to exercise condition; immediate recovery; early recovery
Ren et al. [66]	50 women; 25 newly diagnosed Graves’ hyperthyroidism and 25 healthy controls; mean age about 40 years; body mass index about 23 kg per square meter	Controlled acute study; newly diagnosed Graves’ hyperthyroidism versus healthy controls; single repeated high-intensity intermittent exercise session	Targeted outcomes: Interleukin 6; interleukin 15; tumor necrosis factor alpha	Control level: Limited/unclear standardization Feeding: Dietary restrictions: no high-sugar or high-fat diets or snacks during standardized lifestyle week; fasting not reported Time: Not reported Recent/stimulants: Avoided strenuous exercise for 24 h before the test; standardized lifestyle for one week Sleep/circadian: Sleep from 23:00 to 7:00 during standardized lifestyle week Sex/hormonal: Not reported	Matrix specified; Detection/CV not specified Matrix: Serum; capillary blood for glucose and lactate Assay: Enzyme-linked immunosorbent assay for cytokines, leptin, irisin, and creatine kinase; Accutrend Plus for lactate; ACCU-CHEK for glucose Detection/precision: Commercial kit identifiers reported for interleukin 6, interleukin 15, tumor necrosis factor alpha, leptin, irisin, and creatine kinase Plasma-volume correction: Not reported	Coverage: Immediate only Schedule: Before exercise; lactate 1 min after each Wingate test; serum and glucose immediately after third Wingate test Mapped windows: During session; immediate recovery
Rohnejad et al. [67]	22 overweight middle-aged men; 12 high-intensity intermittent training and 10 control; age 40–60 years; body mass index 25–30 kg per square meter	Randomized controlled parallel groups; high-intensity intermittent training versus no-exercise control; single acute training session	Targeted outcomes: Cortisol; interleukin 6; C-reactive protein	Control level: Partial standardization Feeding: Participants asked not to change eating style; specific fasting state not reported Time: Training sessions held from 3:00 to 4:00 p.m./matched control time points Recent/stimulants: Participants asked not to participate in additional exercise program or use supplements/medications Sleep/circadian: Not reported	Matrix specified; Detection/CV incomplete Matrix: Serum, although collection tube description creates matrix ambiguity Assay: Luminescence method for cortisol and interleukin 6; enzymatic kits for aspartate aminotransferase, alanine aminotransferase, creatine kinase, lactate dehydrogenase Detection/precision: Kit suppliers reported; detection limits not reported Plasma-volume correction: Not reported	Coverage: Includes very late (>24 h) Schedule: Before exercise; 1 h, 24 h, and 48 h after exercise/Before exercise; 1 h, 24 h, and 48 h after matched control exposure Mapped windows: Early, late, and very late recovery
Ruegsegger et al. [68]	49 adults (15 lean, 18 obese with normal glucose tolerance, 16 obese with impaired glucose tolerance); age 18–65 years; men and women; obese groups matched by age and body mass index	Two-visit acute study; single session high-intensity interval exercise with group comparisons by glucose tolerance status; fasting at both visits	Focused multi-mediator set: Interleukin 1 beta; interleukin 6; tumor necrosis factor alpha; C-reactive protein	Control level: Partial standardization Feeding: Both study visits were performed after a twelve-hour fast Time: Exercise visit time of day not explicitly reported Recent/stimulants: Exclusion criteria included smoking, structured exercise more than twice weekly, medications affecting energy metabolism or insulin sensitivity, active coronary artery disease Sleep/circadian: Sleep control not reported Sex/hormonal: Not reported; both men and women were included	Matrix specified; Detection/CV reported Matrix: Plasma for glucose and insulin; serum for interleukin 1 beta, interleukin 6, tumor necrosis factor alpha, and C-reactive protein Assay: Glucose and insulin assays as previously described by authors; commercially available enzyme-linked immunosorbent assays from R and D Systems for interleukin 1 Detection/precision: Intra-sample coefficients of variation: glucose, 3.7 percent; insulin, 4.2 percent; interleukin 6, 5.1 percent; interleukin 1 beta, 5.6 percent; tumor necrosis factor alpha, 4 Plasma-volume correction: Not reported	Coverage: Immediate/early only Schedule: Blood biomarker subset sampled 15 min before exercise, immediately after exercise, and 60 min after exercise; cognitive tests 30 min before and 60 min after exercise Mapped windows: Baseline; immediate recovery; early recovery
Rusdiawan et al. [69]	36 male soccer players; age 18–24 years; at least 4 years of competitive experience; training ≥4 sessions per week; randomized to ice compression, sports massage, or passive recovery (12 per group)	Randomized controlled parallel-group study; intermittent exercise, then 15 min halftime recovery intervention (ice compression versus sports massage versus passive recovery)	Targeted outcomes: Interleukin 6	Control level: Stronger standardization Feeding: Participants fasted the night before testing; water was allowed as needed Time: Participants attended health check at 06:00 in the morning Recent/stimulants: No injuries or anti-inflammatory drug use in the preceding month; cardiovascular or metabolic disorders were exclusion criteria Sleep/circadian: Sleep control not reported beyond overnight fasting	Matrix specified; Detection/CV incomplete Matrix: Serum for interleukin 6; capillary blood for lactate Assay: Human interleukin 6 Quantikine enzyme-linked immunosorbent assay; Roche Cobas Accutrend Plus GCTL meter for lactate Detection/precision: Assay precision not reported Plasma-volume correction: Not reported	Coverage: Immediate only Schedule: Before intermittent exercise, immediately after intermittent exercise, and after 15 min recovery intervention Mapped windows: Baseline; immediate recovery
Sasimontonkul et al. [70]	22 overweight premenopausal women; age 30–55 years; body mass index greater than 23 kg per square meter; randomized 11 exercise and 11 control	Longitudinal randomized controlled study; acute 40 min high-intensity interval training session assessed on first and last training days within 16-week training program	Focused multi-mediator set: Interleukin 6; adiponectin; leptin; high-sensitivity C-reactive protein	Control level: Stronger standardization Feeding: Overnight fasting for eight hours before blood sampling Time: Blood collected between 07:00 and 07:30 Recent/stimulants: No explicit stimulant restriction reported; participants were asked to maintain usual dietary intake Sex/hormonal: Premenopausal women with regular menstrual cycle were included; specific menstrual-cycle phase timing was not reported	Matrix specified; Detection/CV reported Matrix: Serum for adiponectin, leptin, interleukin 6, procollagen type 1 N-terminal propeptide, and C-terminal telopeptide of type 1 collagen; plasma for high-sensitivity C-reactive protein Assay: Automated electrochemiluminescence immunoassay for procollagen type 1 N-terminal propeptide and C-terminal telopeptide of type 1 collagen Detection/precision: Intra-assay coefficients of variation: C-terminal telopeptide of type 1 collagen, 1.37 percent; procollagen type 1 N-terminal propeptide, 1.20 percent; adiponectin, 4.7 percent Plasma-volume correction: Not reported	Coverage: Immediate only Schedule: Before and immediately after 40 min high-intensity interval session on day 1 and on final day of 16-week program; resting pre- and post-intervention samples also collected Mapped windows: Immediate recovery; training endpoint
Siasos et al. [71]	20 healthy men; mean age 22.6 ± 3.3 years; crossover design; fasted; avoided intense activity for 48 h	Randomized crossover acute study; continuous moderate-intensity aerobic exercise versus high-intensity interval aerobic exercise on a cycle ergometer, separated by 1 week	Targeted outcomes: Interleukin 17	Control level: Stronger standardization Feeding: Participants attended in a fasted state Time: All exercise sessions and measurements took place at 09:00 Recent/stimulants: Participants avoided intense physical activity for 48 h and caffeine and alcohol for 24 h before each session	Matrix specified; Detection/CV incomplete Matrix: Serum Assay: Commercial enzyme-linked immunosorbent assay kits for interleukin 17 from R&D Systems Detection/precision: Assay precision and detection-limit information not reported in extracted article sections Plasma-volume correction: Not reported	Coverage: Immediate only Schedule: Before and ten minutes after each exercise session Mapped windows: Immediate recovery
Sim et al. [72]	10 trained male triathletes; mean age 23 years; healthy iron status; no iron supplementation	Randomized counterbalanced crossover; four exercise trials (running and cycling at low and high-intensity), each separated by at least 7 days; laboratory-based testing	Focused multi-mediator set: Serum interleukin 6; serum hepcidin; serum iron; serum ferritin.	Control level: Stronger standardization Feeding: Food intake recorded and replicated; iron-free standardized meal provided one hour after exercise Time: Participants arrived at 07:00 Recent/stimulants: No manual labor or structured exercise during the twenty-four hours before each testing session	Matrix specified; Detection/CV reported Matrix: Serum Assay: Serum iron and ferritin by Architect analyzer; interleukin 6 by Quantikine enzyme-linked immunosorbent assay Detection/precision: Reported coefficients of variation: iron, 1.73% and 0.61%; ferritin, 4.58%, 4.46%, and 4.36%; interleukin 6, 5.46% and 8.95%; hepcidin, 2.7% intrarun and 6.5% inter-run Plasma-volume correction: Water provided to minimize hemoconcentration effects	Coverage: Immediate/early only Schedule: Baseline, immediately post-exercise, and 3 h post-exercise Mapped windows: Baseline; immediate recovery; early recovery
Uchida et al. [73]	11 healthy young men without regular exercise habits; mean age 22 years; randomized crossover with at least 3 days washout	Randomized crossover acute study; continuous cycling versus interval cycling; saliva and blood collected pre, immediately post, 30 min post, and 24 h post	Focused multi-mediator set: Salivary human herpesvirus 6 DNA; salivary human herpesvirus 7 DNA; whole-blood lactate; serum interleukin 6 change.	Control level: Stronger standardization Feeding: Participants refrained from eating after 22:00 the night before; no drinking until sixty minutes after exercise Time: Measurements conducted from 08:00 to 12:00 after 45 min seated rest Recent/stimulants: No excessive exercise or alcohol for twenty-four hours before maximum oxygen uptake testing and each exercise trial	Matrix specified; Detection/CV not specified Matrix: Saliva; serum; whole blood; not blood-derived for maximum voluntary contraction and subjective fatigue Assay: Salivary human herpesvirus 6 and human herpesvirus 7 DNA by real-time polymerase chain reaction; lactate by Lactate Pro2 Detection/precision: Primer/probe sequences and polymerase-chain-reaction cycling reported; interleukin 6 assay kit reported; assay precision not fully tabulated Plasma-volume correction: Plasma-volume correction not reported	Coverage: Extends to late (6–24 h); no very late Schedule: Before exercise, immediately after exercise, 30 min after exercise, and 24 h after exercise Mapped windows: Baseline; immediate recovery; early recovery; late recovery
Vardar et al. [74]	18 healthy men; 9 physically active and 9 physically inactive; ages 28.7 ± 6.3 and 30.2 ± 4.5 years	Parallel-group acute comparison; physically active versus physically inactive men; same high-intensity interval exercise session	Targeted outcomes: Interleukin 6 messenger RNA expression; tumor necrosis factor alpha messenger RNA expression.	Control level: Stronger standardization Feeding: Not extractable from uploaded abstract page Time: Not extractable from uploaded abstract page Recent/stimulants: Not extractable from uploaded abstract page Sleep/circadian: Not extractable from uploaded abstract page	Matrix specified; Detection/CV reported Matrix: Blood; messenger RNA expression Assay: Quantitative real-time polymerase chain reaction analysis for messenger RNA expression Detection/precision: Assay precision and detection information not extractable from uploaded abstract page Plasma-volume correction: Not extractable from uploaded abstract page	Coverage: Window coverage unclear Schedule: Before exercise, five minutes after high-intensity interval exercise, and twenty-four hours after high-intensity interval exercise Mapped windows: Baseline; 5 min after exercise; 24 h after exercise
Verbickas et al. [75]	20 healthy physically active young men; randomized 10 sprint interval cycling exercise and 10 stretch–shortening cycle exercise	Randomized parallel-group acute comparison; sprint interval cycling exercise versus stretch–shortening cycle exercise	Targeted outcomes: Interleukin 6; interleukin 10.	Control level: Stronger standardization Feeding: Participants refrained from food before baseline measurements Time: Experiment performed between 8:00 and 11:00 in the morning Recent/stimulants: Participants refrained from physical exercise, caffeine, and alcohol for at least twenty-four hours before testing Sleep/circadian: Participants were asked to sleep at least eight hours the night before the experiment	Matrix specified; Detection/CV incomplete Matrix: Serum for brain-derived neurotrophic factor, cortisol, interleukin 6, and interleukin 10; plasma for norepinephrine Assay: Gemini immunoassay enzyme-linked immunosorbent assay analyzer for norepinephrine, brain-derived neurotrophic factor, interleukin 6, and interleukin 10 Detection/precision: Reliability information reported for neuromuscular tests; assay precision values for blood markers not reported in the article text Plasma-volume correction: Not reported	Coverage: Immediate/early only Schedule: Before exercise and two minutes, one hour, twelve hours, and twenty-four hours after exercise/before exercise and two minutes, one hour, twelve hours, and twenty-four hours after exercise Mapped windows: Baseline; immediate recovery; early recovery; 12 h after exercise; 24 h after exercise
Wadley et al. [76]	9 healthy untrained participants analyzed; mean age 29 years; body mass index 24.2 kg per square meter	Randomized crossover acute trial (study 1 only from a two-study paper); moderate-intensity continuous cycling versus high-intensity interval exercise; at least 1 week washout	Broad/exploratory panel: Peroxiredoxin 2; peroxiredoxin 4; superoxide dismutase 3; thioredoxin 1; thioredoxin reductase; interleukin 6	Control level: Stronger standardization Feeding: Participants completed trials after at least a 10 h fast/Participants completed testing after at least a 10 h fast Time: Morning testing between 07:00 and 08:00 Recent/stimulants: Refrained from strenuous physical activity, alcoholic beverages, and caffeine for two days before experimental sessions	Matrix specified; Detection/CV incomplete Matrix: Plasma/Serum Assay: In-house enzyme-linked immunosorbent assays for peroxiredoxin 2, peroxiredoxin 4, thioredoxin 1, thioredoxin reductase, and superoxide dismutase 3 Detection/precision: No cross-reactivity detected in enzyme-linked immunosorbent assay validation; detailed coefficients of variation not reported for redox assays in the article Plasma-volume correction: Adjusted for plasma-volume changes/adjusted for plasma-volume changes where applicable	Coverage: Includes very late (>24 h) Schedule: Before exercise after rest; immediately after exercise; 30 min after exercise; 60 min after exercise/Before exercise after rest; immediately after exercise; 30 min after exercise; 3 h after exercise Mapped windows: Baseline; immediate recovery; early recovery/baseline; immediate recovery; early recovery; very late recovery
Windsor et al. [77]	30 healthy older adults (26 men, 4 women); age 60–86 years; 16 lower-fit and 14 higher-fit	Randomized crossover; non-exercise control, moderate-intensity continuous cycling, and high-intensity interval cycling; stratified by cardiorespiratory fitness group	Targeted outcomes: Interleukin 6; interleukin 10; tumor necrosis factor alpha	Control level: Stronger standardization Feeding: Participants were fasted for 3 h after a standardized snack Time: Testing performed at the same time of day under consistent laboratory conditions Recent/stimulants: Participants refrained from alcohol and caffeine for 12 h and from pro re nata anti-inflammatory medication for 72 h before each visit Sex/hormonal: Not clearly controlled; mixed-sex older adult sample	Matrix specified; Detection/CV reported Matrix: Plasma Assay: Commercial sandwich enzyme-linked immunosorbent assays for interleukin 6, interleukin 10, and tumor necrosis factor alpha Detection/precision: Within-assay coefficients of variation: interleukin 6, 4.7 ± 5.0 percent; interleukin 10, 4.6 ± 5.3 percent; tumor necrosis factor alpha, 4.3 ± 4.3 percent Plasma-volume correction: Not corrected for plasma-volume changes	Coverage: Immediate/early only Schedule: Before protocol; immediately after protocol; 20 min after protocol for interleukin 6 and interleukin 10; 90 min after protocol Mapped windows: Baseline; immediate recovery; early recovery
Zwetsloot et al. [78]	7 healthy recreationally active young men analyzed; 8 completed; age 22 ± 2 years; self-reported physical activity 4 ± 2 days weekly	Single-group repeated-measures training study; acute high-intensity interval training response compared between first and sixth training sessions after 2 weeks	Broad/exploratory panel: Interleukin 6; interleukin 8; interleukin 10; tumor necrosis factor alpha; monocyte chemoattractant protein 1; interferon gamma; granulocyte macrophage-colony stimulating factor; interleukin 1 beta	Control level: Stronger standardization Feeding: Participants reported being well hydrated and were instructed to consume the same diet before both exercise testing visits Time: Testing and training sessions at approximately the same time of day Recent/stimulants: Participants refrained from non-steroidal anti-inflammatory drugs, anti-inflammatory or antioxidant supplements, and other aerobic activities during the study	Matrix specified; Detection/CV reported Matrix: Serum Assay: Bead-based multiplex assay using MAGPIX and xPONENT software Detection/precision: Inter-assay coefficients of variation: granulocyte macrophage-colony stimulating factor, 13.9 percent; interferon gamma, 5.9 percent; interleukin 10, 8.7 percent Plasma-volume correction: Not reported	Coverage: Immediate/early only Schedule: Before exercise; immediately after exercise; 15 min after exercise; 30 min after exercise; 45 min after exercise Mapped windows: Baseline; immediate recovery; early recovery

**Table 2:** Methodological characteristics of the high-intensity interval training regimen.

Study	Arm or Condition	Mode and Context	Work Intervals	Recovery Between Intervals	Dose and Schedule
Abedelmalek et al., [35]	Baseline sleep	Treadmill; morning (08:00)	4 × 250 m running at 80% personal maximal speed	3 min rest between runs	Single session; blood sampling during and after exercise
Abedelmalek et al., [35]	Partial sleep deprivation	Treadmill; morning (08:00); sleep restriction before session	4 × 250 m running at 80% personal maximal speed	3 min rest between runs	Single session; identical exercise dose to baseline sleep
Afzalpour et al. [36]	High-intensity interval training plus ginger	Training: Shuttle running; morning (08:00). Acute test: cycle ergometer	Training: 30 s maximal shuttle run; 4 to 8 repeats (progressed every 2 weeks). Acute test: 4 × 30 s all-out cycling at 0.075 kg per kilogram body mass	Training: 30 s active rest. Acute test: 4 min active rest	Training: 3 sessions weekly for 10 weeks; warm-up and cool-down 5 to 10 min. Acute test performed before training and 72 h after last session
Afzalpour et al. [36]	High-intensity interval training plus placebo	Training: Shuttle running; morning (08:00). Acute test: cycle ergometer	Training: 30 s maximal shuttle run; 4 to 8 repeats (progressed every 2 weeks). Acute test: 4 × 30 s all-out cycling at 0.075 kg per kilogram body mass	Training: 30 s active rest. Acute test: 4 min active rest	Training: 3 sessions weekly for 10 weeks; warm-up and cool-down 5 to 10 min. Acute test performed before training and 72 h after last session
Andersson et al. [37]	Axial spondyloarthritis	Cycle ergometer	4 × 4 min; target above 90% estimated maximum heart rate; rating of perceived exertion above 17	3 min active rest at about 70% maximum heart rate	Single session; same protocol as healthy controls; blood sampled before and 1 h after exercise
Andersson et al. [37]	Healthy controls	Cycle ergometer	4 × 4 min; target above 90% estimated maximum heart rate; rating of perceived exertion above 17	3 min active rest at about 70% maximum heart rate	Single session; same protocol as axial spondyloarthritis group; blood sampled before and 1 h after exercise
Brown et al. [38]	High-intensity intermittent walking	Treadmill walking	3 × 5 min at 80% maximal oxygen uptake	3 × 5 min walking at 30% maximal oxygen uptake	Single session in randomized crossover design; comparator session separated by 7 days
Brzezinska et al. [39]	Ischemic preconditioning	Bilateral thigh cuffs; morning (08:00–10:00), before meal; acute test on cycle ergometer	Intervention: 4 × 5 min occlusion at 220 mm of mercury. Acute test: double Wingate Anaerobic Test; 30 s all-out at 75 g per kilogram body mass	Intervention: 5 min reperfusion between cuff inflations. Acute test: not clearly reported between Wingate bouts	Intervention daily for 14 consecutive days; participants supine; same acute test performed before and after intervention
Brzezinska et al. [39]	Sham control	Bilateral thigh cuffs; morning (08:00–10:00), before meal; acute test on cycle ergometer	Intervention: 4 × 5 min cuff inflation at 20 mm of mercury. Acute test: double Wingate Anaerobic Test; 30 s all-out at 75 g per kilogram body mass	Intervention: 5 min reperfusion between cuff inflations. Acute test: not clearly reported between Wingate bouts	Intervention daily for 14 consecutive days; participants supine; same acute test performed before and after intervention
Cabral-Santos et al. [40]	High-intensity intermittent exercise	Treadmill running	5 km run performed as repeated 1 min bouts at speed associated with peak oxygen uptake	1 min passive recovery between bouts	Single volume-matched session in randomized crossover design; sessions separated by at least 72 h; warm-up 5 min at 50% speed associated with peak oxygen uptake
Cabral-Santos et al. [41]	High-intensity intermittent exercise 1.25 km	Treadmill running	Repeated 1 min bouts at 100% speed associated with peak oxygen uptake; run until 1.25 km completed	1 min passive recovery between bouts	Single session in randomized crossover design; warm-up 5 min at 50% speed associated with peak oxygen uptake; sessions separated by at least 72 h
Cabral-Santos et al. [41]	High-intensity intermittent exercise 2.5 km	Treadmill running	Repeated 1 min bouts at 100% speed associated with peak oxygen uptake; run until 2.5 km completed	1 min passive recovery between bouts	Single session in randomized crossover design; warm-up 5 min at 50% speed associated with peak oxygen uptake; sessions separated by at least 72 h
Casuso et al. [42]	Sprint interval swimming	25 m indoor swimming pool	8 × 30 s all-out swimming	3 min 30 s rest between bouts	Single session in randomized crossover design; performed in the morning; comparator running session separated by 7 to 14 days
Casuso et al. [42]	Sprint interval running	400 m running track	8 × 30 s all-out running	3 min 30 s rest between bouts	Single session in randomized crossover design; performed in the morning; comparator swimming session separated by 7 to 14 days
Collins et al. [43]	High-intensity interval exercise	Cycle ergometer; morning (06:00–09:00); after day off or day shift	30 min as 60 s at 100% peak oxygen uptake	240 s at 50% peak oxygen uptake	Single session in randomized parallel design; workload matched across arms; blood sampled pre-session, immediately post-session, and 30 min and 60 min after
Cullen et al. [27]	Low	Cycle ergometer	35 min continuous cycling at 50% maximal oxygen uptake	Not applicable	Single comparator session within counterbalanced repeated-measures design; blood sampled pre- and immediately post-session
Cullen et al. [27]	Mod	Cycle ergometer	5 × 5 min at 50% maximal oxygen uptake interspersed with 5 × 2 min at 80% maximal oxygen uptake	Alternating low-intensity cycling blocks served as recovery	Single interval comparator session; total duration 35 min; blood sampled pre- and immediately post-session
Cullen et al. [27]	High	Cycle ergometer	5 × 4 min at 80% maximal oxygen uptake	3 min at 50% maximal oxygen uptake between intervals	Single high-intensity interval exercise session; total duration 35 min; blood sampled pre- and immediately post-session
Cruz et al. [44]	Filtered air	Cycle ergometer in environmental exposure chamber	10 × 1 min cycling at 100% maximal oxygen uptake	10 × 1 min at 40% maximal oxygen uptake	Single session after 5 min warm-up at 30 watts; approximately 95 min chamber exposure; randomized counterbalanced crossover
Cruz et al. [44]	Traffic-related air pollution	Cycle ergometer in environmental exposure chamber	10 × 1 min cycling at 100% maximal oxygen uptake	10 × 1 min at 40% maximal oxygen uptake	Single session with identical exercise dose under non-filtered chamber air; randomized counterbalanced crossover
Dorneles et al. [45]	High-intensity interval exercise	Treadmill; morning (09:30–10:30)	10 × 60 s running at 85–90% maximal power output	10 × 75 s at 50% maximal power output	Single session after 5 min warm-up at 60% and 5 min cool-down at 50%; randomized crossover with at least 1 week washout
Dorneles et al. [45]	Moderate-intensity interval exercise	Treadmill; morning (09:30–10:30)	10 × 60 s running at 70–75% maximal power output	10 × 60 s at 50% maximal power output	Single comparator session with identical warm-up and cool-down; randomized crossover with at least 1 week washout
Durrer et al. [46]	High-intensity interval exercise	Cycle ergometer; performed in adults with type 2 diabetes and healthy controls	7 × 1 min cycling at 85% peak power output	1 min cycling at 15% peak power output between intervals	Single session after 4 min warm-up at 30 watts and 3 min cool-down; blood sampled pre-, immediately post-, and 1 h post-session
Dzik et al. [47]	Repeated Wingate anaerobic test	Cycle ergometer; morning testing at least 3 h after light breakfast	3 × 30 s all-out cycling; resistance 0.075 kg per kilogram body mass	5 min rest between bouts	Single session; blood sampled pre-, 15 min post-, and 1 h post-session; lactate also sampled 3 min after first and second bout
Gassner et al. [48]	Resistance-circuit high-intensity interval training	Resistance exercise machines; laboratory; morning testing	3 circuits of rowing, chest press, leg curl, lat pull, and leg press; 40 s per exercise at 50% 1-repetition maximum	10 s to change stations; 1 min between circuits	Single session after 5-repetition maximum testing; target 20 repetitions per exercise with 1 s concentric and 1 s eccentric phases
Gerosa-Neto et al. [49]	High-intensity interval training	Treadmill running	10 × 1 min at 100% maximal aerobic velocity	1 min passive rest	Approximately 300 kilocalories per session; mean duration 19.0 min; 3 sessions weekly for 6 weeks; acute responses assessed in first and last sessions
Gokbel et al. [50]	Repeated Wingate tests	Cycle ergometer; morning testing (09:00–10:00); 2 h after light breakfast	5 × 30 s all-out cycling; resistance 75 g per kilogram body weight	2 min rest between bouts	Single session; blood sampled pre-, immediately post- (within 1 min after fifth test), and 15 min and 60 min post-session
Hall et al. [51]	High-intensity interval exercise	Cycle ergometer; laboratory; type 1 diabetes and healthy control groups	4 × 5 min cycling at 120% lactate threshold	5 min rest between intervals	Single session; capillary and venous blood sampled at rest, immediately post-exercise, and 24 h post-exercise
Herranz-Lopez et al. [52]	High-intensity interval exercise	Elastic resistance bands plus cycle ergometer warm-up	2 × 4 min of 20 s maximal elastic-band exercise with 10 s rest across 8 upper and lower limb exercises; rating of perceived exertion at least 8	10 s between bouts; 3 min rest between series	Single session in randomized crossover design; aerobic session separated by at least 72 h; blood sampled 20 min pre- and post-session
Henke et al. [53]	High-intensity interval training	Cycle ergometer	60 s at 85–90% maximal heart rate	75 s at 40% maximal heart rate between bouts	Warm-up and cool-down 5 min at 50% maximal heart rate; 10 bouts in week 1, 12 bouts in weeks 2–3, 14 bouts in week 4; 2 sessions weekly for 4 weeks; blood sampled before and after first and eighth sessions
Intan et al. [54]	High-intensity interval training	Interval running; supervised; morning fasted	Two sets of 4–6 × 30 s running at 80–95% maximum heart rate	1:3 work-to-recovery at 60–70% maximum heart rate	Warm-up and cool-down 5 min; 3 sessions weekly for 6 weeks; progression from 4 repetitions (weeks 1–2) to 5 (weeks 3–4) to 6 (weeks 5–6); blood sampled before and immediately after first and last sessions
Jakobsson et al. [55]	Supramaximal high-intensity interval training at 60% of maximum mean power output for 6 s	Cycle ergometer; warm-up and cool-down at 30% maximum aerobic power	10 × 6 s at 80–90 revolutions per minute (about 150% maximum aerobic power)	54 s recovery: 24 s passive rest and 30 s active recovery at 30% maximum aerobic power	Single session within randomized crossover; visits separated by at least 48 h and completed within 2 weeks; blood sampled before, iso-time, after, and 30 min after
Jakobsson et al. [55]	Supramaximal high-intensity interval training at 80% of maximum mean power output for 6 s	Cycle ergometer; warm-up and cool-down at 30% maximum aerobic power	10 × 6 s at 80–90 revolutions per minute (about 200% maximum aerobic power)	54 s recovery: 24 s passive rest and 30 s active recovery at 30% maximum aerobic power	Single session within randomized crossover; visits separated by at least 48 h and completed within 2 weeks; blood sampled before, iso-time, after, and 30 min after
Kaspar et al. [56]	High-intensity interval training	Cycle ergometer; supervised; morning (08:00–12:00)	6 × 30 s all-out cycling after 2 min warm-up (<50 watts)	4 min recovery: rest or cycling <30 watts	Single session; randomized order versus endurance training; sessions separated by at least 7 days; blood sampled pre-, 30 min post-, and 2 days post-session
Kon et al. [57]	High-intensity interval training	Cycle ergometer; overnight fasted; morning (07:30–11:30)	4 × 30 s maximal cycling; resistance 7.5% of body weight	4 min passive rest on the ergometer between bouts	Single session; rested 30 min before baseline sample; no food until final sample; blood sampled pre-, immediately post-, and 15 min, 30 min, and 120 min post-session
Kon et al. [58]	Normoxic high-intensity interval exercise	Cycle ergometer; overnight fasted; face mask with room air	4 × 30 s all-out cycling; resistance 7.5% of body weight	4 min passive rest between bouts	Single session within single-blind crossover; trials randomized and at least 1 week apart; blood sampled pre-, immediately post-, and 60 min and 180 min post-session
Kon et al. [58]	Hyperoxic high-intensity interval exercise	Cycle ergometer; overnight fasted; face mask with 60% oxygen	4 × 30 s all-out cycling; resistance 7.5% of body weight	4 min passive rest between bouts	Single session within single-blind crossover; hyperoxia from 10 min pre-exercise until immediately after last bout; trials at least 1 week apart; blood sampled pre-, immediately post-, and 60 min and 180 min post-session
Lira et al. [59]	High-intensity intermittent training	Treadmill running	Repeated 1 min bouts at 100% maximal aerobic speed until 5 km completed	1 min passive recovery between bouts	Warm-up 5 min at 50% maximal aerobic speed; 3 sessions weekly for 5 weeks; acute blood sampled fasting, pre- and immediately, 30 min, and 60 min post-session for first and last training sessions
Meyer et al. [60]	Single maximal test	Cycle ergometer; fasted	1 × 60 s all-out cycling	Not applicable	Single session within randomized crossover against anaerobic training session and control day; no strenuous exercise for 2 days before testing; blood sampled at rest and 15 min, 2 h, and 24 h post-session
Meyer et al. [60]	Anaerobic training session	Cycle ergometer; fasted	1 × 60 s all-out cycling, then 8 × 10 s all-out cycling	10 min after first bout, then 4 min 50 s between 10 s bouts	Single session within randomized crossover against single maximal test and control day; blood sampled at rest and 15 min, 2 h, and 24 h post-session
Minuzzi et al. [61]	High-intensity intermittent exercise	Treadmill running; motorized treadmill at 1% gradient; standardized breakfast	10 × 1 min at 90% maximum aerobic velocity after 5 min warm-up at 40%	1 min passive recovery between bouts	Single session performed once in follicular phase and once in luteal phase, 72 h after phase-specific graded test; blood sampled pre-, immediately post-, and 1 h post-session
Monteiro et al. [62]	High-intensity interval exercise	Treadmill running; afternoon testing	10 × 2 min at 95% peak speed after 5 min warm-up at 5 km per hour	1 min passive rest between bouts	Single session performed 72 h after maximal treadmill test; total 34 min and about 3.3 km; blood sampled at rest and immediately post-exercise
Narciso et al. [63]	High-intensity interval running	Treadmill running; morning; standardized breakfast 1.5 h before exercise	8 × 1 min at 90–100% maximal workload	1 min passive recovery between bouts	Single session within randomized crossover; performed 48–72 h after mode-specific incremental test; blood sampled pre- and 5 min and 60 min post-exercise
Narciso et al. [63]	High-intensity interval cycling	Cycle ergometer; morning; standardized breakfast 1.5 h before exercise	8 × 1 min at 90–100% maximal workload (may drop to 90% if unable to complete at 100%)	1 min passive recovery between bouts	Single session within randomized crossover; performed 48–72 h after mode-specific incremental test; blood sampled pre- and 5 min and 60 min post-exercise
Ottone et al. [64]	High-intensity interval exercise	Cycle ergometer; morning (07:30–08:30); environmental chamber	8 × 60 s at 90% of peak power output	75 s active recovery at 30 watts between bouts	2 min at 30 watts before and after; single session 48 h after maximal test; blood sampled pre- and 30 min and 24 h post-exercise
Panissa et al. [65]	High-intensity intermittent exercise (1 h after breakfast)	Cycle ergometer; standardized breakfast; exercise started 1 h after breakfast	30 s at maximal aerobic power, repeated for 30 min	30 s passive recovery between repetitions	Warm-up 5 min at 40% maximal aerobic power and rest 2 min; session duration 30 min; blood at 1.0, 1.75, 2.5 and 3.25 h after breakfast; lactate 1, 3 and 5 min post-exercise
Panissa et al. [65]	High-intensity intermittent exercise (2.5 h after breakfast)	Cycle ergometer; standardized breakfast; exercise started 2.5 h after breakfast	30 s at maximal aerobic power, repeated for 30 min	30 s passive recovery between repetitions	Warm-up 5 min at 40% maximal aerobic power and rest 2 min; session duration 30 min; blood at 1.0, 1.75, 2.5 and 3.25 h after breakfast; lactate 1, 3 and 5 min post-exercise
Panissa et al. [65]	Steady-state exercise (1 h after breakfast)	Cycle ergometer; standardized breakfast; exercise started 1 h after breakfast	30 min at 50% maximal aerobic power (70–80 revolutions per minute)	Not applicable	Warm-up 5 min at 40% maximal aerobic power and rest 2 min; session duration 30 min; blood at 1.0, 1.75, 2.5 and 3.25 h after breakfast; lactate 1, 3 and 5 min post-exercise
Panissa et al. [65]	Steady-state exercise (2.5 h after breakfast)	Cycle ergometer; standardized breakfast; exercise started 2.5 h after breakfast	30 min at 50% maximal aerobic power (70–80 revolutions per minute)	Not applicable	Warm-up 5 min at 40% maximal aerobic power and rest 2 min; session duration 30 min; blood at 1.0, 1.75, 2.5 and 3.25 h after breakfast; lactate 1, 3 and 5 min post-exercise
Ren et al. [66]	Graves’ hyperthyroidism	Cycle ergometer Wingate test	3 × 30 s all-out cycling at 75 g per kilogram body mass	5 min rest between tests	Single session after 15 min warm-up; blood sampled pre- and immediately post-third test; lactate sampled 1 min after each test
Ren et al. [66]	Healthy controls	Cycle ergometer Wingate test	3 × 30 s all-out cycling at 75 g per kilogram body mass	5 min rest between tests	Single session with identical protocol to Graves’ hyperthyroidism group; blood sampled pre- and immediately post-third test; lactate sampled 1 min after each test
Rohnejad et al. [67]	High-intensity intermittent training	Treadmill running	4 sets × 4 × 30 s at 100% maximal aerobic velocity	30 s active recovery at 50% maximal aerobic velocity; 5 min passive rest between sets	Single acute session after 15 min warm-up; blood sampled before and 1 h, 24 h, and 48 h after exercise
Ruegsegger et al. [68]	Lean normal glucose tolerance	Cycle ergometer; 12 h fast	4 × 4 min at 75% maximal watts after 10 min warm-up	3 min rest at no load between intervals	5 min cool-down; single session; blood sampled 15 min pre-, immediately post-, and 60 min post-exercise (subset)
Ruegsegger et al. [68]	Obese with normal glucose tolerance	Cycle ergometer; 12 h fast	4 × 4 min at 75% maximal watts after 10 min warm-up	3 min rest at no load between intervals	5 min cool-down; single session; blood sampled 15 min pre-, immediately post-, and 60 min post-exercise (subset)
Ruegsegger et al. [68]	Obese with impaired glucose tolerance	Cycle ergometer; 12 h fast	4 × 4 min at 75% maximal watts after 10 min warm-up	3 min rest at no load between intervals	5 min cool-down; single session; blood sampled 15 min pre-, immediately post-, and 60 min post-exercise (subset)
Rusdiawan et al. [69]	Ice compression	Intermittent soccer-simulation; morning field testing	15 s sprinting at 90–95% maximal heart rate (repeated across 7 cycles; 42 min total)	30 s jogging at 60–70% maximal heart rate and 15 s walking at 30–40% maximal heart rate (work-rest ratio 2:1)	15 min localized ice compression (12–15 °C gel packs) applied to quadriceps, hamstrings, and calves; blood sampled at T0 pre-, T1 post-, and T2 post-intervention
Rusdiawan et al. [69]	Sports massage	Intermittent soccer-simulation; morning field testing	15 s sprinting at 90–95% maximal heart rate (repeated across 7 cycles; 42 min total)	30 s jogging at 60–70% maximal heart rate and 15 s walking at 30–40% maximal heart rate (work-rest ratio 2:1)	15 min sports massage by certified therapist using effleurage, petrissage, shaking, tapotement, and walking techniques; moderate pressure 2–3 kg-force per square centimeter; blood sampled T0 pre-, T1 post-, T2 post-intervention
Rusdiawan et al. [69]	Passive recovery	Intermittent soccer-simulation; morning field testing	15 s sprinting at 90–95% maximal heart rate (repeated across 7 cycles; 42 min total)	30 s jogging at 60–70% maximal heart rate and 15 s walking at 30–40% maximal heart rate (work-rest ratio 2:1)	15 min seated passive recovery; blood sampled T0 pre-, T1 post-, T2 post-intervention
Sasimontonkul et al. [70]	High-intensity interval training	Treadmill; supervised laboratory sessions	1 min running at 80–90% heart rate reserve, repeated for 40 min	2 min walking at 50–60% heart rate reserve between running bouts	3 sessions per week for 16 weeks; acute blood sampled pre- and immediately post-session on first and last training days; control group maintained usual activities
Siasos et al. [71]	High-intensity interval aerobic exercise	Cycle ergometer; fasted morning testing (09:00)	30 × 30 s at 100% maximal aerobic work after 3 min warm-up	30 s rest between intervals	Single session within randomized crossover; blood sampled pre- and 10 min post-session; sessions separated by 1 week
Sim et al. [72]	High-intensity interval running	Treadmill running; laboratory conditions	8 × 3 min at 85% peak running velocity (after 8 min warm-up at 50% peak)	1.5 min rest between intervals (2:1 work-rest ratio); 8 min cool-down at 40% peak	40 min total; single session within randomized crossover; trials separated by at least 7 days; blood sampled at baseline and immediately post- and 3 h post-session
Sim et al. [72]	High-intensity interval cycling	Cycle ergometer; laboratory conditions	8 × 3 min at 85% peak cycling power output (after 8 min warm-up at 50% peak)	1.5 min rest between intervals (2:1 work-rest ratio); 8 min cool-down at 40% peak	40 min total; single session within randomized crossover; trials separated by at least 7 days; blood sampled at baseline and immediately post- and 3 h post-session
Uchida et al. [73]	Interval exercise	Cycle ergometer; fasted morning testing	Five sets of 2 min at 90% maximal oxygen uptake (20 min total)	2 min at 50% maximal oxygen uptake between high-intensity bouts	Single session within randomized crossover; washout at least 3 days; saliva and blood sampled pre-, immediately post-, 30 min post-, and 24 h post-session
Vardar et al. [74]	Physically active men	Cycle ergometer	4 repeats of a Wingate test at load 0.050 kg per kilogram body weight	Not reported	Single session; blood sampled pre-exercise, 5 min post-exercise, and 24 h post-exercise
Vardar et al. [74]	Physically inactive men	Cycle ergometer	4 repeats of a Wingate test at load 0.050 kg per kilogram body weight	Not reported	Single session; blood sampled pre-exercise, 5 min post-exercise, and 24 h post-exercise
Verbickas et al. [75]	Sprint interval cycling exercise	Cycle ergometer; morning laboratory testing	12 × 5 s all-out stationary cycling sprints	3 min rest between sprints	Single session; blood sampled pre-exercise and 2 min, 1 h, 12 h, and 24 h post-exercise
Verbickas et al. [75]	Stretch–shortening cycle exercise	Drop jumps from 0.5 m height; morning laboratory testing	200 intermittent drop jumps	30 s between jumps	Single session; blood sampled pre-exercise and 2 min, 1 h, 12 h, and 24 h post-exercise
Wadley et al. [76]	High-intensity interval exercise	Cycle ergometer; morning fasted laboratory testing	10 × 4 min cycling at 85% maximal oxygen uptake	2 min rest between intervals	Single session within randomized crossover; 58 min total; energy-matched to continuous trial; washout at least 1 week; blood sampled pre- and immediately, 30 min, and 60 min post-exercise
Windsor et al. [77]	High-intensity interval cycling	Cycle ergometer; older adults	12 × 1 min at 70% peak power output	1 min active recovery at 10% peak power output	Single session within randomized crossover; 24 min total; work-matched to continuous trial; blood sampled pre- and immediately, 20 min, and 90 min post-exercise
Zwetsloot et al. [78]	High-intensity interval training	Cycle ergometer	60 s cycling at workload equivalent to 100% peak oxygen uptake	75 s active recovery at 50 watts	3 sessions weekly for 2 weeks; sessions 1–2: 8 intervals, sessions 3–4: 10 intervals, sessions 5–6: 12 intervals; 3 min warm-up and cool-down at 50 watts; blood sampled in sessions 1 and 6

**Table 3:** Rob2 assessment for parallel studies.

Study	D1 Randomization Process	D1b Period/Carryover Effects	D2 Deviations from Intended Interventions	D3 Missing Outcome Data	D4 Measurement of the Outcome	D5 Selection of the Reported Result	RoB 2 Overall
Brown et al. [38]	Some concerns	Low	Low	Low	Some concerns	Some concerns	Some concerns
Cabral-Santos et al. [40]	Some concerns	Low	Low	Low	Some concerns	Some concerns	Some concerns
Cabral-Santos et al. [41]	Some concerns	Low	Low	Low	Some concerns	Some concerns	Some concerns
Casuso et al. [42]	Some concerns	Low	Low	Low	Low	Some concerns	Some concerns
Cruz et al. [44]	Some concerns	Low	Low	Some concerns	Low	Some concerns	Some concerns
Cullen et al. [27]	Some concerns	Low	Low	Low	Low	Some concerns	Some concerns
Dorneles et al. [45]	Some concerns	Low	Low	Low	Low	Some concerns	Some concerns
H Herranz-Lopez et al. [52]	Some concerns	Low	Low	Low	Low	Some concerns	Some concerns
Jakobsson et al. [55]	Some concerns	Some concerns	Low	Low	Low	Some concerns	Some concerns
Kaspar et al. [56]	Some concerns	Low	Low	High	Low	Some concerns	High
Kon et al. [58]	Some concerns	Low	Low	Low	Low	Some concerns	Some concerns
Meyer et al. [60]	Some concerns	Low	Low	Low	Low	Some concerns	Some concerns
Narciso et al. [63]	Some concerns	Low	Low	Some concerns	Low	Some concerns	Some concerns
Panissa et al. [65]	Some concerns	Low	Low	Low	Low	Some concerns	Some concerns
Siasos et al. [71]	Some concerns	Low	Low	Low	Low	Some concerns	Some concerns
Sim et al. [72]	Some concerns	Low	Low	Low	Low	Some concerns	Some concerns
Uchida et al. [73]	Some concerns	Low	Low	Low	Low	Some concerns	Some concerns
Wadley et al. [76]	Some concerns	Low	Low	Low	Low	Some concerns	Some concerns
W Windsor et al. [77]	Some concerns	Low	Low	Low	Low	Some concerns	Some concerns

**Table 4:** Rob2 assessment for crossover studies.

Study	D1 Randomization Process	D2 Deviations from Intended Interventions	D3 Missing Outcome Data	D4 Measurement of the Outcome	D5 Selection of the Reported Result	RoB 2 Overall
Collins et al. [43]	Some concerns	Low	Low	Low	Some concerns	Some concerns
D Dzik et al. [47]	Some concerns	Low	Low	Low	Some concerns	Some concerns
Verbickas et al. [75]	Some concerns	Low	Low	Low	Some concerns	Some concerns

**Table 5:** JBI assessment.

Study	Domain: Temporal Precedence	Domain: Selection/Control	Domain: Outcome Measurement	Domain: Retention	Domain: Statistical Conclusion Validity
Abedelmalek et al., [35]	Low concern	High concern	Some concerns	Some concerns	Low concern
Afzalpour et al. [36]	Low concern	High concern	Low concern	Some concerns	Low concern
Andersson et al. [37]	Low concern	High concern	Some concerns	Some concerns	Low concern
Brzezinska et al. [39]	Low concern	High concern	Some concerns	High concern	Low concern
Durrer et al. [46]	Low concern	High concern	Low concern	Low concern	Low concern
Gassner et al. [48]	Low concern	High concern	Low concern	High concern	Low concern
Gerosa-Neto et al. [49]	Low concern	High concern	Low concern	Low concern	Low concern
Gokbel et al. [50]	Low concern	High concern	Low concern	Low concern	Low concern
Hall et al. [51]	Low concern	High concern	Low concern	Low concern	Low concern
Henke et al. [53]	Low concern	High concern	Low concern	Low concern	Low concern
Intan et al. [54]	Low concern	High concern	Low concern	Low concern	Low concern
Kon et al. [57]	Low concern	High concern	Low concern	Low concern	Low concern
Lira et al. [59]	Low concern	High concern	Some concerns	Low concern	Low concern
Minuzzi et al. [61]	Low concern	High concern	Low concern	Low concern	Low concern
Monteiro et al. [62]	Low concern	High concern	Low concern	High concern	Low concern
Ottone et al. [64]	Low concern	High concern	Low concern	Low concern	Low concern
Ren et al. [66]	Low concern	High concern	Low concern	Low concern	Low concern
Ruegsegger et al. [68]	Low concern	High concern	Low concern	Some concerns	Low concern
Rusdiawan et al. [69]	Low concern	High concern	Low concern	Low concern	Low concern
Sasimontonkul et al. [70]	Low concern	High concern	Low concern	Some concerns	Low concern
Vardar et al. [74]	Low concern	High concern	Low concern	Low concern	Low concern
Zwetsloot et al. [78]	Low concern	High concern	Low concern	High concern	Low concern

**Table 6:** Acute (single-session) effects.

Study	Comparison	Main Outcomes	Sampling Time Points (Time Windows)	Main Statistical Result and Main Finding
Abedelmalek et al., [35]	Baseline night versus partial sleep deprivation night (within-participant); repeated brief sprint interval running	Interleukin 6; tumor necrosis factor alpha; growth hormone; cortisol; testosterone	Before exercise; immediately after first 250 m run (immediate); immediately after fourth 250 m run (immediate); 60 min after exercise (early)	Compared with baseline night, partial sleep deprivation night produced higher growth hormone and testosterone 60 min after exercise (*p* value less than 0.05). Interleukin 6 and tumor necrosis factor alpha were higher during exercise (after the first and fourth run) and at 60 min after exercise (*p* value less than 0.05). Cortisol showed no statistically significant difference between sleep conditions.
Andersson et al. [37]	Axial spondyloarthritis group versus healthy control group; before versus one hour after one bout (between-group and within-participant)	Interleukin 6; vascular endothelial growth factor A; bone morphogenetic protein 7; C-reactive protein; serum protein panel	Before exercise; one hour after exercise (early)	Main effect of time for interleukin 6 from baseline to one hour after exercise (*p* value 0.03); post hoc increase in healthy controls (*p* value 0.04) but not in axial spondyloarthritis (*p* value 0.23). Group difference for vascular endothelial growth factor A (*p* value 0.03), lower in axial spondyloarthritis at both time points with no time effect. Main effect of time for bone morphogenetic protein 7 (*p* value less than 0.001), increased in both groups (axial spondyloarthritis *p* value 0.004; healthy controls *p* value 0.02). No statistically significant changes for the remaining proteins.
Cabral-Santos et al. [40]	High-intensity intermittent 5 km treadmill run versus steady-state 5 km treadmill run (crossover; volume matched)	Interleukin 6; tumor necrosis factor alpha; interleukin 10; cortisol; lactate; non-esterified fatty acids	At rest; immediately after exercise (immediate); 30 min after exercise (immediate); 60 min after exercise (early)	Main effect of time for cortisol (*p* value less than 0.001), with higher concentrations immediately, 30 min, and 60 min after exercise than at rest. Interleukin 6 was higher after high-intensity intermittent exercise than after steady-state exercise (condition effect *p* value 0.012) and increased immediately after exercise (time effect *p* value less than 0.001). Tumor necrosis factor alpha was lower after high-intensity intermittent exercise than after steady-state exercise (condition effect *p* value 0.012) and increased immediately after exercise (time effect *p* value 0.050). Interleukin 10 increased over time (*p* value 0.002) and the interleukin 10 to tumor necrosis factor alpha ratio increased (time effect *p* value 0.015) with a condition-by-time interaction (*p* value 0.002). Non-esterified fatty acids were higher immediately after steady-state exercise than after high-intensity intermittent exercise (interaction *p* value 0.044).
Cabral-Santos et al. [41]	Shorter versus longer high-intensity intermittent treadmill session (1.25 km versus 2.5 km; crossover)	Interleukin 6; interleukin 10; brain-derived neurotrophic factor; monocyte chemoattractant protein 1; glucose; lactate	At rest; immediately after exercise (immediate); 60 min after exercise (early)	Both volumes increased lactate and interleukin 6 immediately after exercise (lactate *p* value less than 0.0001). Interleukin 6 percent increase from rest to immediately after exercise was larger after the 2.5 km session than after the 1.25 km session (*p* value 0.014), and interleukin 6 remained higher at 60 min after the 2.5 km session (*p* value 0.019). Only the 2.5 km session increased interleukin 10 (*p* value 0.023), and interleukin 10 percent increase from immediately after to 60 min after exercise was larger after the 2.5 km session than after the 1.25 km session (*p* value 0.012). Glucose was higher at 60 min after the 1.25 km session than at rest (*p* value 0.007). Brain-derived neurotrophic factor increased immediately after both sessions. Monocyte chemoattractant protein 1 did not change.
Casuso et al. [42]	Sprint interval swimming versus sprint interval running (crossover)	Interleukin 6; interleukin 10; tumor necrosis factor alpha; cortisol; sodium; potassium; lactate dehydrogenase; creatine kinase myocardial band isoform	Before exercise; 3 min after exercise (immediate); 2 h after exercise (intermediate)	Main effect of time for interleukin 6 (*p* value less than 0.001) with a time-by-condition interaction (*p* value 0.012): both protocols increased interleukin 6 3 min after exercise, but only running remained elevated at 2 h; interleukin 6 was lower after swimming than after running at 2 h (*p* value 0.032). Main effect of time for interleukin 10 (*p* value 0.033) and tumor necrosis factor alpha (*p* value less than 0.001), both increased 3 min after exercise. Main effect of time for cortisol (*p* value less than 0.001). Potassium showed a time-by-condition interaction (*p* value less than 0.001) with lower potassium 3 min after swimming than after running (*p* value less than 0.001).
Brown et al. [38]	High-intensity intermittent walking versus continuous moderate walking (randomized crossover)	Interleukin 6; tumor necrosis factor alpha; endothelin 1; lipid hydroperoxides; hydrogen peroxide; ascorbyl radical; alpha-tocopherol; lycopene	Before exercise; immediately after exercise (immediate); 2 h after exercise (intermediate); 4 h after exercise (intermediate); 24 h after exercise (late); 48 h after exercise (very late)	Main effect of time for interleukin 6 (*p* value less than 0.05), increased immediately, 2 h, and 4 h after both conditions and returned to baseline by 24 h; no time-by-condition interaction. Main effect of time for tumor necrosis factor alpha (*p* value less than 0.05), increased from baseline during follow-up with no time-by-condition interaction. Oxidative stress markers (lipid hydroperoxides, hydrogen peroxide, and ascorbyl radical) showed no statistically significant change. Alpha-tocopherol increased at 2 and 4 h (*p* value less than 0.05). Lycopene showed a time-by-condition interaction (*p* value less than 0.05), decreasing at 2 h only after high-intensity intermittent walking.
Collins et al. [43]	High-intensity interval cycling versus moderate-intensity continuous cycling (randomized parallel groups)	Interleukin 1 receptor antagonist; interleukin 6; tumor necrosis factor alpha; glucose; insulin; sleep fragmentation (wake after sleep onset)	Before exercise; immediately after exercise (immediate); 30 min after exercise (immediate); 60 min after exercise (early); sleep assessed the following night	Both exercise conditions increased interleukin 1 receptor antagonist immediately and 30 min after exercise (*p* value less than 0.016). No statistically significant changes were observed for interleukin 6 or tumor necrosis factor alpha (*p* value greater than 0.05), and no statistically significant changes were observed for insulin sensitivity indices (*p* value greater than 0.05). Wake after sleep onset decreased after moderate-intensity continuous cycling (*p* value less than 0.05) but not after high-intensity interval cycling.
Cruz et al. [44]	High-intensity interval cycling in filtered air versus high-intensity interval cycling in traffic-related air pollution (randomized crossover)	Interleukin 6; interleukin 10; tumor necrosis factor alpha; interleukin 10 to tumor necrosis factor alpha ratio; serum metabolome; systolic blood pressure; diastolic blood pressure	Baseline before exercise; 10 min after exercise (immediate); 60 min after exercise (early)	In filtered air, interleukin 10 increased and the interleukin 10 to tumor necrosis factor alpha ratio increased at 60 min after exercise (*p* value less than 0.01). In traffic-related air pollution, interleukin 6 increased 60 min after exercise (*p* value less than 0.01) and there was no increase in the interleukin 10 to tumor necrosis factor alpha ratio. Metabolomics enrichment showed incomplete fatty acid metabolism 10 min after exercise (*p* value less than 0.05) and increased ketone body metabolism 10 min and 60 min after exercise (*p* value less than 0.05) in traffic-related air pollution. The exercise-induced reduction in systolic blood pressure observed in filtered air was not observed 10 min after exercise and was attenuated 60 min after exercise in traffic-related air pollution (*p* value less than 0.05).
Cullen et al. [27]	Three cycling sessions with fixed duration and different intensity and volume (within-participant): low-intensity, moderate-intensity, and high-intensity interval exercise	Interleukin 6 (plasma); interleukin 10 (plasma); interleukin 6 gene expression; interleukin 10 gene expression; interleukin 4 receptor gene expression	Before exercise; immediately after exercise (immediate)	Plasma interleukin 6 increased after exercise, with a larger fold change in high-intensity interval exercise than in low-intensity exercise (*p* value 0.04). The plasma interleukin 6 response was positively correlated with mean exercise intensity (correlation *p* value less than 0.01). No statistically significant changes were observed for plasma interleukin 10, or for interleukin 10 and interleukin 4 receptor gene expression, indicating no detectable systemic anti-inflammatory response after these short sessions.
Dorneles et al. [45]	High-intensity interval running versus moderate-intensity interval running (randomized crossover within-participant; analyzed separately in lean and overweight–obese groups)	Interleukin 1 receptor antagonist; interleukin 6; interleukin 8; interleukin 10; interleukin 17A; C-C motif chemokine ligand 2; leukocyte counts	Before exercise; immediately after exercise (immediate); 30 min after exercise (immediate)	Moderate-intensity interval exercise produced no statistically significant cytokine changes in either group. High-intensity interval exercise decreased interleukin 8 at 30 min after exercise in both lean and overweight–obese men and increased interleukin 10 immediately and 30 min after exercise in both groups (*p* value less than 0.05). In overweight–obese men, interleukin 6 increased immediately and 30 min after high-intensity interval exercise, whereas in lean men interleukin 6 increased only at 30 min. Leukocyte counts increased after exercise, with patterns differing by group and protocol.
Durrer et al. [46]	Before versus after one bout of high-intensity interval cycling (within-participant), compared between type 2 diabetes and healthy control groups	Toll-like receptor 2 expression on monocyte subsets; lipopolysaccharide-stimulated tumor necrosis factor alpha in whole blood; plasma tumor necrosis factor alpha	Before exercise; immediately after exercise (immediate); one hour after exercise (early)	Toll-like receptor 2 surface expression on classical and CD16-positive monocytes decreased immediately after exercise and one hour after exercise compared with before exercise (*p* value less than 0.05). Lipopolysaccharide-stimulated tumor necrosis factor alpha release decreased one hour after exercise (*p* value less than 0.05), and plasma tumor necrosis factor alpha decreased one hour after exercise (*p* value less than 0.05). A group-by-time interaction was reported for plasma tumor necrosis factor alpha, with a larger decrease in healthy controls than in adults with type 2 diabetes (*p* value less than 0.05).
Gassner et al. [48]	Before versus after one resistance-circuit high-intensity interval training session (single group); includes two-hour seated rest control period	Reactive oxygen species; interleukin 6	Baseline after overnight fasting; after two-hour seated rest (before exercise); immediately after exercise (immediate); 15 min after exercise (immediate)	During the two-hour seated rest, reactive oxygen species decreased by 8.5% (*p* value less than 0.001) and interleukin 6 decreased by 12.3% (*p* value less than 0.05). From before exercise to immediately after exercise, reactive oxygen species increased by 12.3% (*p* value less than 0.001) and interleukin 6 increased by 48.1% (*p* value less than 0.05). From immediately after to 15 min after exercise, reactive oxygen species decreased by 6.9% (*p* value less than 0.05) and interleukin 6 decreased by 20.4% (*p* value less than 0.05), indicating partial recovery within 15 min.
Gokbel et al. [50]	Before versus after five repeated supramaximal cycling tests (single group)	Adiponectin; interleukin 6; tumor necrosis factor alpha; myoglobin	Before exercise; immediately after the fifth cycling test (immediate); 15 min after exercise (immediate); 60 min after exercise (early)	Adiponectin decreased 60 min after exercise compared with before exercise and immediately after exercise (*p* value less than 0.05). Interleukin 6 increased immediately, 15 min, and 60 min after exercise compared with before exercise (*p* value less than 0.05), and increased further at 60 min compared with 15 min (*p* value less than 0.05). Tumor necrosis factor alpha showed no statistically significant change (*p* value greater than 0.05). Myoglobin showed no statistically significant change (*p* value greater than 0.05).
Hall et al. [51]	Before versus after one high-intensity interval exercise session (within-participant), compared between type 1 diabetes and healthy control groups	Blood glucose; hypoxia-inducible factor 1 alpha; vascular endothelial growth factor; tumor necrosis factor alpha	At rest before exercise; immediately after exercise (immediate); 24 h after exercise (late)	Glycemia showed a group effect (*p* value 0.01), with higher resting blood glucose in type 1 diabetes than in healthy controls (*p* value 0.04). In type 1 diabetes, blood glucose decreased from before to immediately after exercise (*p* value 0.03), with no statistically significant difference at 24 h compared with before exercise (*p* value 0.59). Hypoxia-inducible factor 1 alpha showed a group effect (*p* value 0.01) and a group-by-time interaction (*p* value 0.01); resting concentrations were higher in type 1 diabetes than in healthy controls (*p* value 0.01), with no statistically significant within-group changes after exercise (*p* value greater than 0.05). Tumor necrosis factor alpha showed a group-by-time interaction (*p* value 0.04) but no statistically significant post hoc differences. Vascular endothelial growth factor showed a group effect (*p* value 0.02) and a group-by-time interaction (*p* value 0.01); concentrations immediately after exercise were lower in type 1 diabetes than in healthy controls (*p* value 0.02), with no statistically significant within-group changes after exercise.
Herranz-Lopez et al. [52]	Aerobic cycling session versus high-intensity interval training session (randomized order crossover); pre- versus 20 min post-exercise for each session	Interleukin 1 beta; interleukin 2; interleukin 4; interleukin 6; interleukin 7; interleukin 8; interleukin 10; interleukin 17 A; interleukin 22; tumor necrosis factor alpha; interferon gamma; protein carbonyls	20 min before exercise; 20 min after exercise (immediate)	After the aerobic session, interleukin 2 increased in participants with high habitual physical activity (*p* value less than 0.05), with no statistically significant pre-to-post changes in the remaining cytokines or protein carbonyls (*p* value greater than 0.05). After the high-intensity interval training session, interleukin 2 increased in participants with high habitual physical activity (*p* value less than 0.05), with no statistically significant pre-to-post changes in the remaining cytokines or protein carbonyls (*p* value greater than 0.05). Interleukin 22 was not detected before or after the single sessions.
Dzik et al. [47]	Maximal oxygen uptake test to exhaustion versus three repeated 30 s Wingate anaerobic tests (between-participant randomized)	25-hydroxyvitamin D3; parathyroid hormone; interleukin 6; lactate; non-esterified fatty acids; glycerol	Before exercise; 15 min after exercise (immediate); one hour after exercise (early). Additional lactate sampling: 3 min after first and second Wingate anaerobic tests	25-hydroxyvitamin D3 increased in all boys 15 min after exercise (*p* value 0.016) and one hour after exercise (*p* value 0.011). In pubertal boys performing repeated Wingate anaerobic tests, 25-hydroxyvitamin D3 increased 15 min after exercise (*p* value 0.032). Interleukin 6 increased 15 min after both exercise tests (*p* value 0.003) and remained elevated one hour after exercise only after repeated Wingate anaerobic tests (*p* value 0.015). Parathyroid hormone changed over time in pubertal boys after repeated Wingate anaerobic tests, with a decrease one hour after exercise compared with 15 min after exercise (*p* value 0.030). Glycerol increased 15 min after repeated Wingate anaerobic tests (*p* value 0.00001) and decreased by one hour after exercise (*p* value 0.00008).
Jakobsson et al. [55]	Supramaximal interval cycling versus moderate-intensity continuous cycling (randomized crossover for supramaximal interval cycling at 60% of maximum mean power output for 6 s and moderate-intensity continuous cycling); additional supramaximal interval cycling at 80% of maximum mean power output for 6 s	Plasma brain-derived neurotrophic factor; clusterin; hepatocyte growth factor; interleukin 6; lactate; dyspnoea	Before exercise; during exercise at iso-time (end of supramaximal interval cycling compared with 10 min of moderate-intensity continuous cycling); immediately after exercise (immediate); 30 min after exercise (immediate)	Plasma brain-derived neurotrophic factor increased during supramaximal interval cycling and moderate-intensity continuous cycling in both groups, with no statistically significant difference between exercise modalities and no statistically significant difference between groups. The mean relative increase was 59% (range 30% to 87%). When normalized per minute of exercise at target power, supramaximal interval cycling produced a 5-fold to 10-fold greater increase than moderate-intensity continuous cycling, but this difference was not consistently statistically significant. Responses of clusterin, hepatocyte growth factor, lactate, and interleukin 6 varied across sessions, with no consistent evidence of superior responses in one exercise modality.
Kon et al. [57]	Before versus after one all-out high-intensity interval cycling session (single group)	C1q and tumor necrosis factor-related protein 1; C1q and tumor necrosis factor-related protein 9; high-molecular-weight adiponectin; tumor necrosis factor alpha; glucose; free fatty acids	Before exercise; immediately after exercise (immediate); 15 min after exercise (immediate); 30 min after exercise (immediate); 120 min after exercise (early)	C1q and tumor necrosis factor-related protein 9 increased immediately after exercise (*p* value less than 0.05). C1q and tumor necrosis factor-related protein 1 increased 120 min after exercise compared with before exercise (*p* value less than 0.01). High-molecular-weight adiponectin showed no statistically significant change. Tumor necrosis factor alpha increased immediately after exercise and 15 min after exercise (*p* value less than 0.01). Glucose increased immediately after exercise and 15 min after exercise (*p* value less than 0.01). Free fatty acids decreased 30 min after exercise and increased 120 min after exercise compared with before exercise (*p* value less than 0.01).
Kon et al. [58]	All-out high-intensity interval cycling under 60% oxygen versus room air (single-blind crossover)	Derivatives of reactive oxygen metabolites; lipid peroxide; biological antioxidant potential; heat shock protein 27; interleukin 6; tumor necrosis factor alpha	Before exercise; immediately after exercise (immediate); 60 min after exercise (early); 180 min after exercise (intermediate)	Derivatives of reactive oxygen metabolites and lipid peroxide increased immediately after exercise in both trials (*p* value less than 0.01), with smaller percentage increases under 60% oxygen than under room air (*p* value less than 0.05). Heat shock protein 27 increased immediately after exercise in both trials (*p* value less than 0.05) and the exercise-induced increase was smaller under 60% oxygen than under room air (*p* value less than 0.05). Biological antioxidant potential increased immediately after exercise in both trials (*p* value less than 0.01) with no statistically significant difference between oxygen conditions. Interleukin 6 increased 60 min and 180 min after exercise (*p* value less than 0.01) and tumor necrosis factor alpha increased immediately after exercise (*p* value less than 0.01), with no statistically significant differences between oxygen conditions for these cytokines.
Kaspar et al. [56]	Endurance cycling session versus high-intensity interval cycling session (randomized order crossover; at least 7 days apart)	C-reactive protein; interleukin 1 beta; interleukin 6; interleukin 10; monocyte chemoattractant protein 1; insulin-like growth factor 1; interleukin 6 to interleukin 10 ratio	Before exercise; 30 min after exercise (immediate); 2 days after exercise (very late)	Wilcoxon signed-rank tests showed no statistically significant changes in C-reactive protein, interleukin 1 beta, interleukin 6, interleukin 10, or insulin-like growth factor 1 after either session (*p* value greater than 0.05). After endurance cycling, the interleukin 6 to interleukin 10 ratio decreased at 30 min after exercise compared with before exercise (*p* value 0.047), and monocyte chemoattractant protein 1 decreased at 2 days after exercise compared with before exercise (*p* value 0.03). Between-session comparisons at 30 min showed only trends: C-reactive protein was higher after endurance cycling than after high-intensity interval cycling (*p* value 0.1), and interleukin 6 was lower after endurance cycling than after high-intensity interval cycling (*p* value 0.09).
Meyer et al. [60]	Single 60 s all-out cycling test versus anaerobic training session with one 60 s all-out cycling test plus eight 10 s all-out cycling tests; compared with a no-exercise control day (randomized order crossover)	Neutrophils; CD16-positive monocytes (premacrophages); interleukin 6; interleukin 8; C-reactive protein; cortisol	At rest; 15 min after exercise (immediate); 2 h after exercise (intermediate); 24 h after exercise (late)	Wilcoxon tests showed a larger neutrophil increase 2 h after the anaerobic training session than after the single 60 s test (*p* value less than 0.01). CD16-positive monocytes increased earlier after the single 60 s test (15 min after exercise; *p* value less than 0.01 versus control day) and later after the anaerobic training session (2 h after exercise; *p* value less than 0.05 versus control day). Interleukin 6 increased markedly 15 min after the anaerobic training session (*p* value 0.002 versus single 60 s test and control day) and remained elevated 2 h after the anaerobic training session (*p* value 0.004 versus control day; *p* value 0.009 versus single 60 s test); interleukin 8 showed no statistically significant change. C-reactive protein increased 24 h after the anaerobic training session (*p* value 0.02) with no such increase after the single 60 s test. Cortisol increased after the anaerobic training session (*p* value 0.003 versus rest; *p* value 0.002 versus control day) and showed only a small increase after the single 60 s test versus control day (*p* value 0.002).
Minuzzi et al. [61]	High-intensity intermittent treadmill exercise performed in the follicular phase versus luteal phase (within-participant repeated sessions); before versus after one bout in each phase	Tumor necrosis factor alpha; interleukin 6; interleukin 10; interleukin 17; tumor necrosis factor alpha to interleukin 10 ratio; interleukin 10 to tumor necrosis factor alpha ratio	Before exercise; immediately after exercise (immediate); one hour after exercise (early)	Baseline tumor necrosis factor alpha and interleukin 10 were higher in the luteal phase than in the follicular phase (*p* value less than 0.01). Mixed-model analysis showed a main effect of time for interleukin 6 (*p* value less than 0.0001), with lower concentrations one hour after exercise than before and immediately after exercise in both phases. Tumor necrosis factor alpha showed a main effect of time (*p* value less than 0.001), with a decrease one hour after exercise versus before exercise in the luteal phase (*p* value less than 0.05) and no phase-by-time interaction. Interleukin 10 showed a phase-by-time interaction (*p* value less than 0.01): interleukin 10 decreased from before to immediately after exercise and from before to one hour after exercise in the luteal phase (*p* value less than 0.01), and interleukin 10 was lower one hour after exercise than immediately after exercise in the follicular phase (*p* value less than 0.05). Interleukin 17 showed no statistically significant change.
Monteiro et al. [62]	Before versus immediately after one treadmill high-intensity interval exercise session (single group), compared between girls and boys	Interleukin 6; interleukin 10; tumor necrosis factor alpha; cortisol; triacylglycerol; glucose; non-esterified fatty acids; plasminogen activator inhibitor 1; lipoprotein profile	At rest; immediately after exercise (immediate)	Two-way analysis of variance showed a main effect of time for cortisol (F 9.018; *p* value 0.008), triacylglycerol (F 25.189; *p* value less than 0.0001), and interleukin 6 (F 6.543; *p* value 0.020). Interleukin 6 increased from rest to immediately after exercise in girls (*p* value 0.040) but not in boys (*p* value 0.615). No statistically significant time, group, or interaction effects were observed for interleukin 10, tumor necrosis factor alpha, cholesterol, glucose, non-esterified fatty acids, or plasminogen activator inhibitor 1 (*p* value greater than 0.05).
Narciso et al. [63]	High-intensity interval running versus high-intensity interval cycling (randomized order crossover)	Interleukin 6; interleukin 10; tumor necrosis factor alpha; tumor necrosis factor alpha to interleukin 10 ratio; leptin; adiponectin	Before exercise; 5 min after exercise (immediate); 60 min after exercise (early)	Time effect for interleukin 6, with higher concentrations at 5 min (*p* value 0.032) and 60 min after exercise (*p* value 0.001) than before exercise in both exercise modes. Time effect for interleukin 10, with higher concentrations at 5 min (*p* value 0.025) and 60 min after exercise (*p* value less than 0.001) than before exercise in both exercise modes. Tumor necrosis factor alpha showed an exercise mode by time interaction (*p* value less than 0.001), with a peak at 5 min after running but not after cycling. Tumor necrosis factor alpha-to-interleukin 10 ratio decreased 60 min after exercise (*p* value 0.001). Leptin decreased 60 min after exercise (*p* value 0.004) and adiponectin showed no statistically significant change after adjustment for multiple comparisons (adjusted *p* value 0.082).
Ottone et al. [64]	Before versus after one high-intensity interval cycling session (single group)	Neutrophil phagocytic capacity; neutrophil reactive oxygen species generation; cluster of differentiation 11b; cluster of differentiation 16; reduced glutathione in neutrophils; superoxide dismutase activity in neutrophils; plasma interleukin 8; plasma lactate dehydrogenase	Before exercise; 30 min after exercise (immediate); 24 h after exercise (late)	Neutrophil reactive oxygen species generation in response to yeast increased 24 h after exercise (*p* value 0.03), and neutrophil phagocytic capacity increased 24 h after exercise (*p* value 0.006). Cluster of differentiation 11b and cluster of differentiation 16 expression showed no statistically significant change (*p* value 0.8 and *p* value 0.2, respectively). Reduced glutathione in neutrophils decreased 24 h after exercise (*p* value 0.02) and superoxide dismutase activity increased 24 h after exercise (*p* value 0.002). Plasma interleukin 8 increased 24 h after exercise (*p* value 0.01). Other measured plasma cytokines showed no statistically significant change.
Panissa et al. [65]	High-intensity intermittent exercise and steady-state exercise performed at two post-breakfast timings (1 h or 2.5 h) versus no-exercise control (randomized crossover)	Absolute energy intake; relative energy intake; hunger; insulin; interleukin 6; blood lactate	Blood at 1.0, 1.75, 2.5, and 3.25 h after breakfast; exercise started at 1.0 or 2.5 h after breakfast and lasted about 37 min. Post-exercise sampling occurred about 8 min after exercise (immediate) and up to about 98 min after exercise (early). Ad libitum meal at 3.5 h after breakfast	Effect of condition for absolute energy intake (F4, 49.3 = 3.90; *p* value 0.007), with higher energy intake in the no-exercise control condition than after high-intensity intermittent exercise performed 2.5 h after breakfast (*p* value 0.008). Effect of condition for relative energy intake (F4, 49.3 = 10.84; *p* value less than 0.001), with higher relative energy intake in the no-exercise control condition than after steady-state exercise performed 2.5 h after breakfast (*p* value 0.048), high-intensity intermittent exercise performed 1 h after breakfast (*p* value 0.014), and high-intensity intermittent exercise performed 2.5 h after breakfast (*p* value less than 0.001). Effect of condition for hunger (F4, 39.4 = 3.76; *p* value 0.005), with higher hunger in the no-exercise control condition than after high-intensity intermittent exercise performed 2.5 h after breakfast (*p* value 0.007). Condition-by-time interaction for insulin (F12, 247 = 3.37; *p* value less than 0.001) and for interleukin 6 (F12, 57.1 = 2.53; *p* value 0.009). Blood lactate increased after exercise in all exercise conditions (*p* value less than 0.001) and was higher after high-intensity intermittent exercise than after steady-state exercise (*p* value less than 0.001).
Ren et al. [66]	Graves’ hyperthyroidism group versus healthy control group; before versus after three repeated 30 s Wingate tests (between-group and within-participant)	Peak power; mean power; blood lactate; blood glucose; leptin; irisin; creatine kinase; interleukin 6; interleukin 15; tumor necrosis factor alpha	Before exercise; 1 min after Wingate test 1, test 2, and test 3 for blood lactate (immediate); immediately after the third Wingate test for blood glucose, leptin, irisin, creatine kinase, interleukin 6, interleukin 15, and tumor necrosis factor alpha (immediate)	Peak power decreased across the three tests (time effect: F = 410.698; *p* value less than 0.001) with no group effect (*p* value 0.212) and no time-by-group interaction (*p* value 0.278). Mean power decreased across the three tests (time effect: F = 381.635; *p* value less than 0.001) with a time-by-group interaction (F = 15.024; *p* value less than 0.001), indicating different fatigue patterns between groups. Blood lactate increased across tests (time effect: F = 923.837; *p* value less than 0.001) with no time-by-group interaction (*p* value 0.171). Blood glucose increased from before to after exercise (time effect: F = 129.664; *p* value less than 0.001) with no time-by-group interaction (*p* value = 0.416). Leptin increased from before to after exercise (time effect: F = 145.293; *p* value less than 0.001) with a time-by-group interaction (F = 6.181; *p* value 0.016). Irisin increased from before to after exercise (time effect: F = 57.247; *p* value less than 0.001) and creatine kinase increased from before to after exercise (time effect: F = 18.729; *p* value less than 0.001), with no time-by-group interaction for either outcome (*p* value 0.797 and *p* value 0.496, respectively). Interleukin 6 increased from before to after exercise (time effect: F = 44.448; *p* value less than 0.001) with a group effect (F = 21.435; *p* value less than 0.001) and a time-by-group interaction (F = 6.155; *p* value 0.017). Interleukin 15 and tumor necrosis factor alpha showed group effects (F = 6.600; *p* value 0.013 and F = 11.808; *p* value 0.001) but no statistically significant time effects (*p* value 0.054 and *p* value 0.977, respectively) and no time-by-group interactions (*p* value 0.166 and *p* value 0.184, respectively).
Rohnejad et al. [67]	Training group versus control group; before versus one hour, 24 h, and 48 h after one high-intensity intermittent treadmill session (parallel groups)	Cortisol; interleukin 6; C-reactive protein; creatine phosphokinase; lactate dehydrogenase; alanine aminotransferase; aspartate aminotransferase	Before exercise; one hour after exercise (early); 24 h after exercise (late); 48 h after exercise (very late)	Group-by-time interaction was statistically significant for interleukin 6 (*p* value 0.01), C-reactive protein (*p* value 0.015), creatine phosphokinase (*p* value 0.001), lactate dehydrogenase (*p* value 0.020), alanine aminotransferase (*p* value 0.002), and aspartate aminotransferase (*p* value 0.004), but not for cortisol (*p* value 0.26). Within the training group, cortisol (*p* value 0.001), interleukin 6 (*p* value 0.001), creatine phosphokinase (*p* value 0.005), lactate dehydrogenase (*p* value 0.01), alanine aminotransferase (*p* value 0.003), and aspartate aminotransferase (*p* value 0.001) increased at one hour after exercise; C-reactive protein did not increase (*p* value 0.2). At 24 h after exercise, interleukin 6 (*p* value 0.001) and creatine phosphokinase (*p* value 0.001) remained elevated; at 48 h after exercise, only creatine phosphokinase remained elevated (*p* value 0.001). Between groups, interleukin 6, creatine phosphokinase, alanine aminotransferase, and aspartate aminotransferase were higher in the training group than the control group at one hour after exercise (*p* value 0.01, 0.01, 0.04, and 0.04, respectively).
Ruegsegger et al. [68]	Lean adults with normal glucose tolerance versus obese adults with impaired glucose tolerance; before versus after one high-intensity interval cycling session	Composite cognitive function score; interleukin 6; C-reactive protein; insulin; glucose	Cognitive testing: 30 min before exercise and 60 min after exercise (early). Blood biomarkers (subset): 15 min before exercise; immediately after exercise (immediate); 60 min after exercise (early)	Executive function and working memory improved after exercise in lean adults and obese adults with normal glucose tolerance (*p* value less than 0.05) but not in obese adults with impaired glucose tolerance. The change in composite cognitive score differed by group, with larger improvements in lean adults and obese adults with normal glucose tolerance than in obese adults with impaired glucose tolerance (*p* value less than 0.01). After adjustment for body size and body composition, two-hour glucose during an oral glucose tolerance test was negatively associated with the change in composite cognitive score (partial correlation rp equals minus 0.398; *p* value 0.007). Interleukin 6 and C-reactive protein increased immediately after exercise in all groups, then returned to pre-exercise levels at 60 min after exercise in lean adults and obese adults with normal glucose tolerance but remained elevated at 60 min after exercise in obese adults with impaired glucose tolerance; 60 min changes in interleukin 6 and C-reactive protein were greater in obese adults with impaired glucose tolerance than in the other groups (*p* value less than 0.05). Insulin decreased immediately after exercise in all groups and did not differ from pre-exercise at 60 min; insulin at 60 min after exercise was negatively associated with the change in composite cognitive score (correlation r equals minus 0.60; *p* value less than 0.01).
Rusdiawan et al. [69]	Ice compression versus sports massage versus passive recovery during a 15 min recovery period after standardized intermittent exercise (parallel groups)	Blood lactate; interleukin 6	Before intermittent exercise; immediately after intermittent exercise (immediate); 15 min after intermittent exercise following the recovery intervention (immediate)	Mixed-model analysis of variance showed a statistically significant effect of intervention for blood lactate and interleukin 6 (*p* value less than 0.001). Post-intervention comparisons: interleukin 6 was lower after ice compression than after passive recovery (*p* value 0.001) and lower after sports massage than after passive recovery (*p* value less than 0.001), with no statistically significant difference between ice compression and sports massage (*p* value 0.898). Blood lactate was lower after ice compression than after passive recovery (*p* value less than 0.001) and lower after sports massage than after passive recovery (*p* value 0.001), and blood lactate was lower after ice compression than after sports massage (*p* value 0.023).
Siasos et al. [71]	Continuous moderate-intensity aerobic cycling versus high-intensity interval aerobic cycling (randomized order crossover)	Augmentation index of the aortic pressure waveform; interleukin 17	Before exercise; 10 min after exercise (immediate)	Augmentation index improved after continuous moderate-intensity aerobic exercise (*p* value 0.04) but did not change after high-intensity interval aerobic exercise (*p* value 0.65). Interleukin 17 increased after continuous moderate-intensity aerobic exercise (*p* value 0.042) but did not change after high-intensity interval aerobic exercise (*p* value 0.47). The increase in interleukin 17 was inversely associated with the improvement in augmentation index after continuous moderate-intensity aerobic exercise (*p* value 0.05).
Sim et al. [72]	Low-intensity continuous running versus low-intensity continuous cycling versus high-intensity interval running versus high-intensity interval cycling (randomized crossover)	Interleukin 6; hepcidin; serum iron; serum ferritin	Baseline before exercise; immediately after exercise (immediate); 3 h after exercise (intermediate)	Main effect of time for serum interleukin 6, with increases immediately after exercise in all trials (*p* value less than 0.05). Post-exercise interleukin 6 was higher after high-intensity interval running than after low-intensity continuous running (*p* value less than 0.05). Main effect of time for hepcidin, with increases 3 h after exercise in all trials (*p* value less than 0.05) and no trial effect (*p* value greater than 0.05). Serum iron increased immediately and 3 h after exercise in all trials except low-intensity continuous cycling (*p* value less than 0.05). Serum ferritin increased immediately and 3 h after exercise in low-intensity continuous running and high-intensity interval running (*p* value less than 0.05).
Uchida et al. [73]	Continuous cycling at 70% maximal oxygen uptake versus interval cycling with alternating 50% and 90% maximal oxygen uptake (randomized crossover)	Salivary human herpesvirus 6 deoxyribonucleic acid expression; salivary human herpesvirus 7 deoxyribonucleic acid expression; serum interleukin 6; blood lactate; maximal voluntary contraction	Before exercise; immediately after exercise (immediate); 30 min after exercise (immediate); 24 h after exercise (late)	Salivary human herpesvirus 6 increased immediately after exercise (*p* value less than 0.001) and 30 min after exercise (*p* value 0.002) after interval cycling, and was higher after interval cycling than after continuous cycling at both time points (*p* value 0.002 and *p* value 0.048). Salivary human herpesvirus 7 showed no statistically significant between-trial change. Changes in serum interleukin 6 and blood lactate were higher after interval cycling than after continuous cycling immediately after exercise (*p* value less than 0.001). The sum of time-dependent changes in maximal voluntary contraction from before exercise to 24 h after exercise was lower after interval cycling than after continuous cycling (*p* value 0.016). The change in salivary human herpesvirus 6 from before exercise to 24 h after exercise was negatively correlated with the change in maximal voluntary contraction (Spearman correlation coefficient minus 0.349; *p* value 0.047).
Vardar et al. [74]	Physically active group versus inactive group; before versus after one high-intensity interval exercise session (between-group and within-participant)	Interleukin 6 messenger ribonucleic acid; tumor necrosis factor alpha messenger ribonucleic acid; heat shock protein 60 messenger ribonucleic acid; heat shock protein 70 messenger ribonucleic acid; B cell lymphoma 2 messenger ribonucleic acid; Bcl-2-associated X protein messenger ribonucleic acid	Before exercise; 5 min after exercise (immediate); 24 h after exercise (late)	At 5 min after exercise, interleukin 6 messenger ribonucleic acid and tumor necrosis factor alpha messenger ribonucleic acid were higher in inactive men than in physically active men (*p* value 0.003 and *p* value 0.007). Heat shock protein 60 messenger ribonucleic acid was higher in inactive men than in physically active men 5 min after exercise (*p* value 0.027). Heat shock protein 70 messenger ribonucleic acid increased only in physically active men (*p* value 0.024). The increases in B cell lymphoma 2 and Bcl-2-associated X protein messenger ribonucleic acid were higher in inactive men than in physically active men 5 min after exercise (*p* value 0.047 and *p* value 0.024).
Verbickas et al. [75]	Sprint interval cycling versus stretch–shortening cycle exercise (parallel groups)	Brain-derived neurotrophic factor; cortisol; norepinephrine; interleukin 6; interleukin 10; maximal voluntary contraction torque	Before exercise; 2 min after exercise (immediate); one hour after exercise (early); 12 h after exercise (late); 24 h after exercise (late)	Brain-derived neurotrophic factor, cortisol, norepinephrine, and interleukin 6 increased more at 2 min after sprint interval cycling than after stretch–shortening cycle exercise (*p* value less than 0.05). Brain-derived neurotrophic factor and cortisol decreased at 24 h after both protocols (*p* value less than 0.05), with a larger decrease after stretch–shortening cycle exercise than after sprint interval cycling (*p* value less than 0.05). Interleukin 6 increased at 2 min after stretch–shortening cycle exercise and one hour after sprint interval cycling (*p* value less than 0.05), and remained higher at 12 h after both protocols (*p* value less than 0.05). Interleukin 10 showed no statistically significant change after either protocol.
Wadley et al. [76]	Moderate-intensity continuous cycling versus high-intensity interval cycling (energy and time matched; randomized crossover)	Peroxiredoxin 2; peroxiredoxin 4; superoxide dismutase 3; thioredoxin 1; thioredoxin reductase; interleukin 6	Before exercise; immediately after exercise (immediate); 30 min after exercise (immediate); 60 min after exercise (early)	Trial-by-time interaction for superoxide dismutase 3 (F(3,1) = 5.3; *p* value 0.028): superoxide dismutase 3 increased after high-intensity interval exercise, peaking immediately and 30 min after exercise, and was higher than after moderate-intensity exercise at 30 min after exercise (*p* value less than 0.05). Peroxiredoxin 4 increased after high-intensity interval exercise at 30 min (*p* value 0.015) and 60 min after exercise (*p* value 0.008), and was higher than after moderate-intensity exercise at all post-exercise time points (*p* value 0.038). Thioredoxin reductase decreased after high-intensity interval exercise immediately after exercise (*p* value 0.021) and decreased after moderate-intensity exercise at 60 min after exercise (*p* value 0.038). No statistically significant time effects were observed for peroxiredoxin 2 or thioredoxin 1. Interleukin 6 increased after both trials (time effect: F(3) = 15.5; *p* value 0.0001) and the increase was larger after high-intensity interval exercise than after moderate-intensity exercise (trial-by-time interaction: F(3) = 7.0; *p* value 0.001).
Windsor et al. [77]	Moderate-intensity continuous cycling versus higher-intensity interval cycling versus seated control (randomized crossover); compared between higher-fit and lower-fit groups	Interleukin 6; interleukin 10; tumor necrosis factor alpha	Before protocol; immediately after protocol (immediate); 20 min after protocol (immediate); 90 min after protocol (early)	Baseline interleukin 6 was higher in fitter participants than in less fit participants (group effect: *p* value 0.02). Interleukin 6 increased immediately after all protocols (time effect: *p* value 0.02) and interleukin 10 increased immediately after all protocols (time effect: *p* value less than 0.01), with no statistically significant protocol-by-time or group-by-time interactions for either cytokine (*p* value greater than 0.05), indicating no measurable exercise-specific cytokine response beyond seated control. Tumor necrosis factor alpha showed no statistically significant changes over time and no statistically significant protocol effects (*p* value greater than 0.05).

**Table 7:** Training intervention effects (pre–post). Studies with multi-week training.

Study	Training Arms	Main Outcomes	Assessment Points	Main Statistical Result and Main Finding
Afzalpour et al. [36]	High-intensity interval training plus ginger (3 g daily, 10 weeks, 3 sessions per week); high-intensity interval training plus placebo (10 weeks, 3 sessions per week); ginger only (3 g daily, no training)	Maximum oxygen consumption; percent body fat; serum intercellular adhesion molecule 1; serum monocyte chemotactic protein 1; serum interleukin 10	Pre-training and post-training: venous blood immediately before and immediately after an acute high-intensity interval cycling protocol (4 × 30 s all-out cycling with 4 min active rest). Body composition and maximum oxygen consumption assessed pre-training and post-training	Maximum oxygen consumption increased after training in both training groups (within-group *p* value 0.002 for high-intensity interval training plus ginger; *p* value less than 0.001 for high-intensity interval training plus placebo), with a between-group difference in change (one-way analysis of variance *p* value less than 0.001). Percent body fat decreased only in the high-intensity interval training-plus-ginger group (within-group *p* value 0.04), with a between-group difference (one-way analysis of variance *p* value 0.005). For inflammatory markers, the acute exercise-related increase in intercellular adhesion molecule 1 after training was higher in the high-intensity interval training-plus-placebo group than in the high-intensity interval training-plus-ginger group (between-group *p* value 0.02), consistent with modest attenuation by ginger with training. Monocyte chemotactic protein 1 and interleukin 10 responses showed no statistically significant between-group differences (*p* value greater than 0.05).
Brzezinska et al. [39]	Ischemic preconditioning (14 consecutive days; 4 cycles of 5 min thigh occlusion at 220 mm of mercury with 5 min reperfusion, daily) versus sham-controlled (same schedule; 20 mm of mercury)	Ferritin; hepcidin; erythroferrone; serum iron; growth differentiation factor 15; interleukin 15; follistatin-like protein 1; insulin-like growth factor 1; soluble amyloid precursor protein alpha; brain-derived neurotrophic factor; anaerobic performance	Pre-intervention and post-intervention: blood at rest (before exercise), immediately after a double Wingate anaerobic test, and 2 h after exercise. Resting biomarkers also compared pre-intervention versus post-intervention	Compared with sham-controlled group, ischemic preconditioning increased resting ferritin (about 9%; *p* value less than 0.05), hepcidin (about 12%; *p* value less than 0.05), and erythroferrone (about 10%; *p* value less than 0.05), with significant group-by-time interactions for these markers. Anaerobic performance showed no statistically significant change (*p* value greater than 0.05). After intervention, ischemic preconditioning altered acute post-exercise responses: greater immediate increases in growth differentiation factor 15 and interleukin 15 (*p* value less than 0.05 versus sham-controlled), faster normalization of follistatin-like protein 1 by 2 h (*p* value less than 0.05), higher post-exercise insulin-like growth factor 1 release (about 8%; *p* value 0.03) and higher soluble amyloid precursor protein alpha release (about 10%; *p* value 0.04). Brain-derived neurotrophic factor was lower 2 h after exercise in ischemic preconditioning group than in sham-controlled group (*p* value less than 0.05).
Gerosa-Neto et al. [49]	High-intensity interval training (6 weeks, 3 sessions per week; 10 × 1 min at 100% maximal aerobic velocity with 1 min passive recovery; about 300 kilocalories per session) versus moderate-intensity continuous training (6 weeks, 3 sessions per week; 65% maximal aerobic velocity for energy-matched duration)	Serum interleukin 6; serum interleukin 10; serum tumor necrosis factor alpha; serum macrophage inflammatory protein 1 alpha; glucose; insulin; homeostatic model assessment of insulin resistance; oral glucose tolerance test; lipopolysaccharide-stimulated whole blood interleukin 10 and tumor necrosis factor alpha	Pre-training and post-training fasting blood. Acute sessions in the first and last training sessions: fasting; pre-exercise (90 min after standardized breakfast); immediately after exercise; 30 min after exercise; 60 min after exercise. Lipopolysaccharide-stimulated whole blood assayed at rest, immediately after exercise, 30 min after exercise, and 60 min after exercise	Fasting metabolic and inflammatory variables showed no statistically significant pre-training-to-post-training change (*p* value greater than 0.05). During acute sessions, repeated-measures analysis showed a main effect of time for serum interleukin 6 (F = 9.300; *p* value less than 0.001) and serum interleukin 10 (F = 6.231; *p* value 0.001), with interleukin 6 remaining elevated up to 60 min and interleukin 10 higher at 30 min and 60 min than at rest (*p* value 0.001). Area under the curve for serum interleukin 10 differed by training arm (F = 6.112; *p* value 0.023), with higher values after moderate-intensity continuous training than after high-intensity interval training. Lipopolysaccharide-stimulated whole-blood interleukin 10 showed a time effect with higher secretion immediately after exercise (F = 6.229; *p* value 0.003), with no condition effects for tumor necrosis factor alpha. Homeostatic model assessment of insulin resistance decreased only after exclusion of two least responsive participants (*p* value 0.020).
Henke et al. [53]	High-intensity interval training on a cycle ergometer (4 weeks, 2 sessions per week; 60 s at 85% to 90% maximal heart rate with 75 s at 40% maximal heart rate; progressed from 10 to 14 work bouts)	Interleukin 1 beta; interleukin 1 receptor antagonist; interleukin 6; interleukin 10; monocyte chemoattractant protein 1; thiobarbituric acid reactive substances; nitrites; advanced oxidation protein products; peak oxygen consumption	Pre-training and post-training resting blood (before exercise). Acute responses assessed before and immediately after the first and eighth training sessions. Peak oxygen consumption assessed pre-training and post-training	After 4 weeks of training, resting interleukin 6 was lower (*p* value less than 0.001) and resting interleukin 10 and interleukin 1 receptor antagonist were higher (*p* value less than 0.001 and *p* value 0.03, respectively). In the first session, monocyte chemoattractant protein 1, interleukin 6, and interleukin 10 increased immediately after exercise (*p* value less than 0.001), with no statistically significant acute change in interleukin 1 beta or interleukin 1 receptor antagonist (*p* value greater than 0.05). In the eighth session, interleukin 1 receptor antagonist, interleukin 6, and interleukin 10 increased immediately after exercise (*p* value 0.02, *p* value 0.01, and *p* value 0.001, respectively), with no statistically significant acute change in monocyte chemoattractant protein 1 or interleukin 1 beta (*p* value greater than 0.05). For oxidative stress markers, thiobarbituric acid reactive substances and advanced oxidation protein products increased after the first session (*p* value 0.009 and *p* value 0.03) but after 4 weeks only advanced oxidation protein products increased after exercise (*p* value 0.042) and no statistically significant resting changes were observed.
Intan et al. [54]	High-intensity interval training (6 weeks, 3 sessions per week; interval running with two sets of 4 to 6 × 30 s at 80% to 95% maximum heart rate with recovery at 60% to 70% maximum heart rate; total 20 to 25 min) versus moderate-intensity continuous training (6 weeks, 3 sessions per week; continuous running 40 to 60 min at 60% to 75% maximum heart rate)	Maximum oxygen consumption (estimated); plasma interleukin 6 response to exercise	Maximum oxygen consumption measured pre-training and post-training (two days after the last training session). Venous blood collected before and immediately after exercise in the first training session and in the last training session (end of week 6)	Maximum oxygen consumption increased after training in both groups (paired-comparison *p* value less than 0.001 for both), with no statistically significant between-group difference in change (*p* value 0.292). The acute pre-to-post-exercise change in plasma interleukin 6 was not statistically significant in the first training session (high-intensity interval training *p* value 0.845; moderate-intensity continuous training *p* value 0.846; between-group *p* value 0.912) and was not statistically significant in the last training session (high-intensity interval training *p* value 0.178; moderate-intensity continuous training *p* value 0.704; between-group *p* value 0.255). Overall, training improved maximum oxygen consumption without clear evidence of a change in the acute interleukin 6 response.
Lira et al. [59]	High-intensity intermittent training (5 weeks, 3 sessions per week; 5 km treadmill running as 1 min at 100% maximal aerobic speed with 1 min passive recovery) versus steady-state training (5 weeks, 3 sessions per week; 5 km treadmill running at 70% maximal aerobic speed) versus control (no training intervention)	Glucose; non-esterified fatty acids; interleukin 6; interleukin 10; tumor necrosis factor alpha	Fasting blood pre-training and post-training. Acute sessions in the first and last training sessions: overnight fasting; pre-exercise after standardized breakfast; immediately after exercise; 30 min after exercise (interleukin 10 only); 60 min after exercise	For fasting values, a group effect was reported for glucose (F = 5.29; *p* value 0.012), with higher concentrations in the control group than in the two training groups. In acute sessions, tumor necrosis factor alpha and glucose were higher immediately after exercise than pre-exercise (time effect), independent of training period and exercise intensity. Interleukin 6 showed a group-by-time interaction: increases occurred immediately after and 60 min after high-intensity intermittent exercise, whereas in steady-state exercise the increase was observed only at 60 min, independent of training period. Interleukin 10 showed a training-period-by-time interaction, with a different recovery profile after training and higher immediate post-exercise interleukin 10 after training than before training, independent of exercise intensity, indicating that 5 weeks of training modified the acute interleukin 10 response.
Sasimontonkul et al. [70]	High-intensity interval training (16 weeks, 3 sessions per week; 40 min treadmill alternation of 1 min running at 80% to 90% heart rate reserve with 2 min walking at 50% to 60% heart rate reserve) versus control (routine daily activities)	Adiponectin; leptin; interleukin 6; N-terminal propeptide of type 1 procollagen; cross-linked C-terminal telopeptide of type I collagen; bone mineral density (tibia, femur neck, lumbar spine); high-sensitivity C-reactive protein	Resting assessments pre-intervention and post-intervention: fasting blood and bone mineral density. Acute assessments: first and last training-day bouts with blood collected before and immediately after the 40 min session; adiponectin, leptin, and interleukin 6 assessed only on the last day	Acute effects in the exercise group: N-terminal propeptide of type 1 procollagen increased immediately after the first bout (*p* value 0.001) and the last bout (*p* value 0.039), while cross-linked C-terminal telopeptide of type I collagen did not change (*p* value 0.137 and *p* value 0.598). On the last day, interleukin 6 increased immediately after exercise (*p* value less than 0.001), while adiponectin and leptin did not change (*p* value 0.824 and *p* value 0.423). Training effects: Resting adiponectin increased in the exercise group (*p* value 0.048) and bone mineral density did not change in the exercise group (*p* value greater than 0.05). In the control group, tibial bone mineral density decreased (*p* value 0.020) with an increase in resting cross-linked C-terminal telopeptide of type I collagen (*p* value 0.010).
Zwetsloot et al. [78]	High-intensity interval training (2 weeks, 3 sessions per week; 60 s cycling intervals at workload equivalent to 100% maximal oxygen uptake with 75 s active recovery at 50 watts; progressed from 8 to 12 intervals per session)	Interleukin 6; interleukin 8; interleukin 10; tumor necrosis factor alpha; monocyte chemotactic protein 1; interleukin 1 beta; interferon gamma; granulocyte macrophage colony-stimulating factor; maximal oxygen uptake; peak cycling power	Pre-training and post-training maximal graded exercise tests. Acute inflammatory response assessed during training session 1 and training session 6 with serum collected at rest and immediately, 15, 30, and 45 min after exercise	Acute effects: Interleukin 6, interleukin 8, tumor necrosis factor alpha, monocyte chemotactic protein 1, and interleukin 10 increased after high-intensity interval training sessions compared with rest (time effects *p* value less than or equal to 0.002), while interleukin 1 beta, interferon gamma, and granulocyte macrophage colony-stimulating factor did not change. Training did not alter the acute inflammatory response (training effects *p* value 0.694 to 0.833; interaction effects *p* value 0.614 to 0.880). After 2 weeks, peak power increased by 4.6% (*p* value 0.007), while maximal oxygen uptake did not change (*p* value 0.481). Heart rate and rating of perceived exertion during the eighth interval were lower in session 6 than in session 1 (*p* value 0.014 and *p* value 0.028).

**Table 8:** Moderator and main synthesis for interpretation of acute cytokine responses.

Moderator Domain	Dominance in Current Synthesis	How It Resolves Conflicting Findings	Main Limitation
Sampling window	Very high	Immediate and early sampling most often detects IL-6 responses; later inflammatory, redox, acute-phase, and cellular-functional responses may be missed when studies stop at ≤30–60 min.	Intermediate, late, and very late windows were sparsely sampled.
Exercise dose and internal load	Very high	Differences in interval intensity, total high-intensity work, work-to-rest ratio, recruited muscle mass, lactate accumulation, glycogen depletion, sympathetic activation, and recovery status can produce subthreshold, adaptive, or stress-dominant cytokine patterns.	Protocols were too heterogeneous for formal nonlinear meta-regression.
Metabolic phenotype and substrate availability	High	Fasting/fed state, carbohydrate availability, glycogen status, adiposity, insulin sensitivity, diabetes status, glucose tolerance, lactate, insulin, glucose, and non-esterified fatty acids can change both baseline inflammatory tone and the interpretation of IL-6 as an immunometabolic mediator rather than a purely pro-inflammatory cytokine. Exercise-induced IL-6 production is linked to contracting skeletal muscle and is amplified under low-glycogen conditions.	Direct muscle-glycogen and substrate-kinetic measures were rare.
Training status and baseline inflammatory burden	High	Trained participants may require a larger relative or novel stimulus to generate a measurable systemic cytokine signal, whereas sedentary, obese, older, or clinical cohorts may show different baseline inflammation, immune-cell responsiveness, and recovery kinetics.	Most studies were underpowered for interaction testing.
Sex and hormonal context	Moderate	Female-only studies and menstrual-cycle-controlled studies were sparse, limiting interpretation of sex-specific or ovarian-hormone-dependent cytokine kinetics.	Male-only cohorts predominated, and menstrual-cycle or contraceptive status was often incompletely reported.
Circadian, sleep, and time-of-day context	Moderate to high	Clock time, sleep duration, chronotype, shift-work context, cortisol rhythm, catecholamine state, and leukocyte trafficking can modify baseline cytokine concentrations and post-exercise responsiveness. Human studies show diurnal variation in circulating IL-6 and circadian neuroendocrine regulation of leukocyte subsets.	Few studies directly compared morning versus evening exercise or measured circadian phase.
Blood matrix, assay platform, and plasma-volume correction	High for small effects	Serum/plasma differences, multiplex versus ELISA platforms, detection limits, handling of non-detects, and plasma-volume correction can determine whether small cytokine changes are classified as increase, decrease, or no clear change.	Assay precision, detection limits, and matrix assignment were inconsistently reported.
Cellular source, receptor context, and immune-cell activation state	Moderate to high mechanistic relevance	Circulating cytokine abundance cannot determine whether the signal originates predominantly from skeletal muscle, leukocytes, endothelium, adipose tissue, or hepatic sources, nor whether target cells activate classical IL-6 signaling, trans-signaling, STAT3, MAPK/ERK, NF-κB, or other pathways. IL-6 biology depends on membrane IL-6 receptor, soluble IL-6 receptor, gp130, and soluble gp130 context.	Paired receptor, phospho-signaling, single-cell, and functional immune assays were uncommon.

**Table 9:** Summary of findings and certainty of evidence by outcome.

Outcome	Studies Contributing to the Evidence	Tendency of the Finding	Main Reasons for Rating	Certainty
Interleukin 6	38	Most consistent mediator-level signal; increases were concentrated in immediate and early recovery windows, although not universal across clinical and older cohorts.	Risk of bias and imprecision downgraded; inconsistency not serious enough to negate direction of effect.	Moderate
Tumor necrosis factor alpha	20	Responses were heterogeneous: increases occurred in some sprint or high-intensity protocols, but many studies reported no clear change.	Downgraded for inconsistency, risk of bias, and imprecision.	Low
Interleukin 10	19	Responses were variable and appeared more dependent on protocol volume, timing, and context than on interleukin 6.	Downgraded for inconsistency and imprecision; indirectness minor because outcomes were directly measured.	Low
Interleukin 8	4	Rapid increases were observed in selected protocols, but the evidence base was sparse.	Downgraded for imprecision and limited consistency assessment.	Very low
Monocyte chemoattractant protein 1	6	No consistent acute directional pattern across populations and protocols.	Downgraded for inconsistency and imprecision.	Very low
Interleukin 1 beta and interleukin 1 receptor antagonist	5 and 3	Evidence suggested context-dependent or absent acute responses; later sampling was limited.	Downgraded for sparse data and inconsistency.	Very low
C-reactive protein	5	Most informative when sampling extended beyond immediate recovery, but late windows were uncommon.	Downgraded for indirectness of immediate sampling and imprecision.	Very low
Redox and oxidative-stress markers	4	Marker-dependent findings; high-intensity cycling increased selected extracellular redox enzymes in one study, while other markers were inconsistent.	Downgraded for inconsistency, sparse outcome-specific replication, and figure-only values in some studies.	Very low

## Data Availability

No new data were created or analyzed in this study. Data sharing is not applicable to this article.

## Abbreviations

The following abbreviations are used in this manuscript:

HIIT	High-intensity interval training
CRP	C-reactive protein
